# Computational Analysis of Structure – Activity Relationships in Highly Active Homogeneous Ruthenium-based Water Oxidation Catalysts

**DOI:** 10.3390/catal12080863

**Published:** 2022-08-05

**Authors:** Gabriel Bury, Yulia Pushkar

**Affiliations:** 1 Department of Physics and Astronomy, Purdue University, West Lafayette, Indiana 47907

**Keywords:** Density Functional Theory, Water Oxidation, Homogeneous Catalysis, Reactive intermediates, Ruthenium, Volcano Plot, Scaling Relationships, Sabatier Principle

## Abstract

Linear free energy scaling relationships (LFESRs) and regression analysis may predict the catalytic performance of heterogeneous and recently, homogenous water oxidation catalysts (WOCs). This study analyses twelve homogeneous Ru-based catalysts – some, the most active catalysts studied: the Ru(tpy-R)(QC) and Ru(tpy-R)(4-pic)_2_ catalysts, where tpy is 2,2:6,2-terpyridine, QC is 8-quinolinecarboxylate and 4-pic is 4-picoline. Typical relationships studied among heterogenous and solid-state catalysts cannot be broadly applied to homogeneous catalysts. This subset of structurally similar catalysts with impressive catalytic activity deserves closer computational and statistical analysis of energetics correlating with measured catalytic activity. We report general methods of LFESR analysis yield insufficiently robust relationships between descriptor variables. However, volcano plot-based analysis grounded in Sabatier’s principle reveals ranges of ideal relative energies of the Ru^IV^=O and Ru^IV^-OH intermediates and optimal changes in free energies of water nucleophilic attack on Ru^V^=O. A narrow range of Ru^IV^-OH to Ru^V^=O redox potentials corresponding with the highest catalytic activities suggests facile access to the catalytically competent high-valent Ru^V^=O state, often inaccessible from Ru^IV^=O. Our work introduces experimental oxygen evolution rates into approaches of LFESR and Sabatier principle-based analysis, identifying a narrow yet fertile energetic landscape to bountiful oxygen-evolution activity, leading future rational design.

## 1. Introduction

The harvesting of sunlight offers a gateway into a sustainable energy future by providing a clean means to satiate the world’s growing hunger for energy while providing a solution to the developing global climate change concerns [1]. The current challenge is in the harvesting and utilization of solar energy efficiently. Generation of solar fuels via artificial photosynthetic processes is an attractive method for harvesting this sunlight [2–6]. Numerous conceptual schemes have been proposed in which sunlight drives the flow of electrons and protons, leading to O-O bond formation and the evolution of molecular oxygen, O_2_; the coupling of this proton and electron movement to water oxidation catalysts (WOCs) encourages the breaking of bonds in water and leads to formation of H_2_ and O_2_ [7,8]. The most significant bottleneck the entire catalytic process of light-driven water oxidation is oxygen-evolving reaction (OER) [9]:

(1) $$2{\text{H}}_{2}\text{O}\to {\text{O}}_{2}+4{\text{H}}^{+}+4{\text{e}}^{-}\;\left({\text{E}}^{0}=1.23\text{V versus NHE at pH }0\right).$$

Current catalysts do not satisfy the need for cost efficiency, activity, and stability for this endergonic reaction; currently studied catalysts suffer from limited catalytic activity due to significant overpotentials (≥400mV). As such, research into the development and design of sufficiently stable and efficient catalysts capable of facilitating solar-driven water oxidation has grown significantly in recent years [10–16].

**Figure 1.**
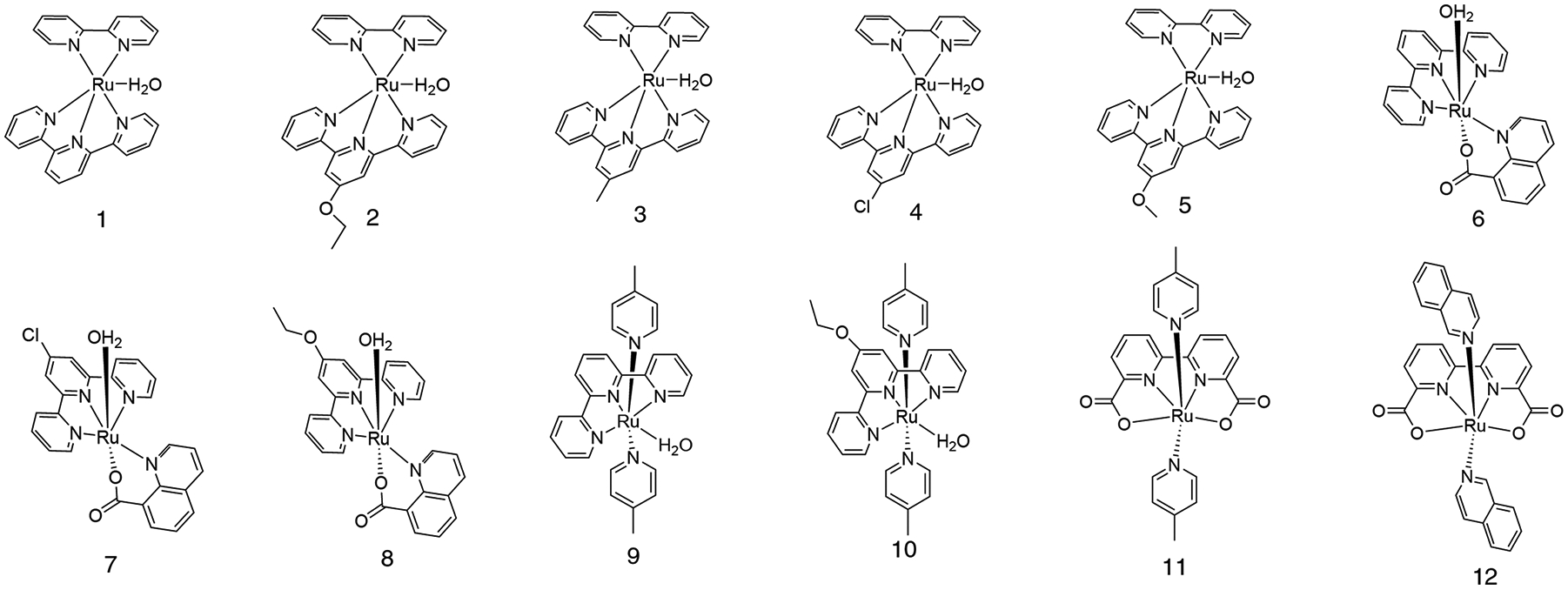
A schematic representation of the complexes considered in this study. **1)** Ru(tpy)(bpy) **2)** Ru(tpyEtO)(bpy) **3)** Ru(tpyMe)(bpy) **4)** Ru(tpy-Cl)(bpy) **5)** Ru(tpyMeO)(bpy) **6) *trans-***Ru(tpy)(QC) **7) *trans-***Ru(tpy-Cl)(QC) **8) *trans-***Ru(EtOtpy)(QC) **9)** Ru(tpy)(4-pic)_2_ **10)** Ru(EtOtpy)(4-pic)_2_ **11)** Ru(bda)(4-pic)_2_ **12)** Ru(bda)(isoq)_2_.

Historically, many catalysts were discovered through trial and error through synthesis and experimentation, though a more sophisticated approach adopts the mentality of rational design – development and design of novel catalysts with consideration of known catalytic theory to increase the likelihood of synthesizing an effective catalyst [17,18]. If computational theory and modelling may be employed to study and successfully characterize candidate catalysts and predict their catalytic competence, significant time can be spared on synthesizing and experimentally characterizing ineffective options. With the rapid improvement in the speed and capabilities of computational software and technologies, such an approach is becoming very alluring. Work has been done in the computational characterization of broad ranges of catalytic families and systems [19–21]. Popular are scaling relationships, employed to describe metal-organic frameworks [22,23], single-atom catalysts [24,25], and other heterogenous [26,27] and homogenous [28–30] systems. These scaling relationships relate parameters of a catalyst or its mechanism with predictors of strong catalytic activity, either oxidation rate, (low) overpotentials, turnover frequencies, or reduced energetic barriers in key mechanistic steps. The use of linear scaling relationships serves to reduce some of the systemic error [31–33] inherent to some computational methods, such as density functional theory (DFT), which may differ in their treatment of static correlation and electron localization. Since scaling relationships predict the relative activity of the catalysts, these systemic errors prove less significant in most cases. Despite the demonstrated need [34] for specifically chosen scaling relationships in homogenous catalysis based on ligand motifs, recent proposals still describe universal [35] scaling relationships for WOCs. Such reports are absent for the Ru(NNN)(NN)-based and similarly structured family of catalysts, Figure 1, home to some very highly active catalysts. Our computational analysis includes numerous Ru(NNN)(NN)-based catalysts, as well as Ru(bda)(N)(N) and several Ru(NNN)(QC) catalysts – some of the most active for Ru-based water oxidation catalysis. Analyzed complexes contain neutral polypyridine ligands alongside negatively charged QC (−1) and bda (−2) ligands, Figure 1.

**Figure 2.**
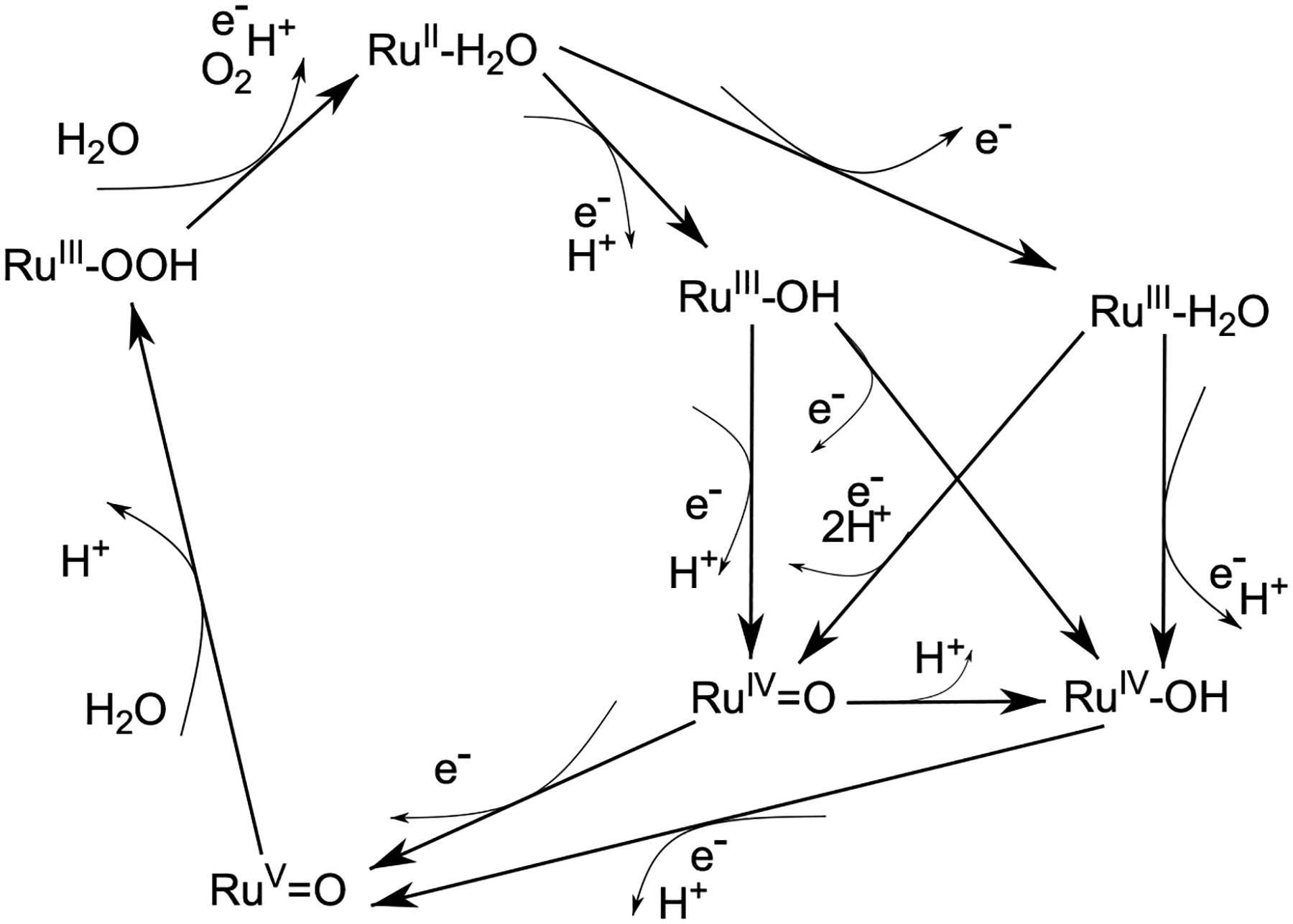
Possible pathways for oxidation and proton coupled electron transfer of a WOC forming O-O bond via water nucleophilic attack on the Ru^V^=O state.

The OER process occurs via a series of redox steps involving various reaction intermediates. The metal center coordinates water and is successively oxidized. Later, an O-O bond is formed, and molecular oxygen is ultimately released. Two primary paths have been proposed for O-O bond formation: water nucleophilic attack (WNA) and the interaction of two metal-oxo moieties (I2M) [17,36,37]; below, Figure 2 outlines a mechanism for a generic WOC, forming an O-O bond through a WNA process on the high-valent Ru^V^=O state.

Both methods of O-O bond formation are preceded by proton coupled oxidation, which comprises of three electron transfer events (ET) and two proton transfer events. Often, ET events are coupled to the transfer of a proton at the same time, in which case this proton-coupled electron transfer (PCET) allows for the reduction of energetic barriers during charge transfer. In the case of WNA, a metal-oxo species undergoes the attack of a solvent water molecule with a proton transfer, succeeded by further electron transfer prior to, or in concert with, release of molecular oxygen (details of these later steps were not investigated); I2M mechanisms simply involve coupling of two metal-oxo groups, resulting the O-O bond requisite for O_2_ evolution. A substantial history of work on Ru-based WOCs [38–42] suggest that both WNA and I2M processes occur more easily upon reaching the high-valent Ru^V^=O state. Such studies describe the catalytic cycles leading to O_2_ evolution in roughly four steps, each characterized by the removal of one electron:

(2) $${\text{Ru}}^{\text{II}}{\text{-H}}_{2}\text{O}\to {\text{Ru}}^{\text{III}}\text{-OH}+{\text{H}}^{+}+{\text{e}}^{-},$$

(3) $${\text{Ru}}^{\text{III}}\text{-OH}\to {\text{Ru}}^{\text{IV}}=\text{O}+{\text{H}}^{+}+{\text{e}}^{-},$$

(4) $${\text{Ru}}^{\text{IV}}=\text{O}+{\text{H}}_{2}\text{O}\to {\text{Ru}}^{\text{III}}\text{-OOH}+{\text{H}}^{+}+{\text{e}}^{-}\text{, and}$$

(5) $${\text{Ru}}^{\text{III}}\text{-OOH}\to {\text{Ru}}^{\text{II}}+{\text{O}}_{2}+{\text{H}}^{+}+{\text{e}}^{-}.$$

From the relative Gibbs energies in the reactions above, changes in free energies for each reaction step, ΔG_i_, leads to the determination of the most thermodynamically difficult step and by extension, the theoretical overpotential [17]:

(6) $$\eta_{\text{th}}=\text{Max}\left[{\Delta \text{G}}_{\text{i}}\right]-1.23\text{V}.$$

This implies that the ideal catalyst, with η_th_= 0, would have distributed overall change in free energy equally across each step in the reaction process; ΔG_i_ = 1.23V for all i. For most Ru-based catalysts, O-O bond formation from Ru^IV^ intermediates via WNA or I2M processes is difficult; access to the high-valent Ru^V^=O intermediate lowers barriers sufficiently to allow for O-O bond formation. However, in many Ru-based WOCs, direct oxidation from Ru^IV^=O to Ru^V^=O occurs at high potentials (≥1.7V) [41,43,44]. At the same time some Ru-based WOCs boast very high rates of oxygen evolution. This suggests that the Ru^V^=O state might be achievable in some WOCs. Figure 2 posits an alternate pathway to this single-electron oxidation step from Ru^IV^=O. For Ru^IV^-OH species produced either by an ET step from Ru^III^ or via protonation of the Ru^IV^=O species, a PCET step at a much more accessible potential (~1.4–1.6V) can be driven by sacrificial oxidants such as cerium(IV) ammonium nitrate (CAN) or electrochemically [45]. Numerous high-valent Ru^V^=O species of catalytically competent WOCs have been observed through use of electron paramagnetic resonance (EPR) [43,46–49]. Ru^V^ EPR features are rhombic and have g-tensors near (g_xx_ ~2.1, g_yy_ ~2.0, g_zz_ ~1.9) [41], though one recent study report a significantly different, highly anisotropic EPR signal assigned to Ru^V^ [50] which we suggest is better describe as a complex of Ru^III^ with modified ligand.

Based on known mechanisms of WOC action, we tailor the generalized conventions of LFESR analysis – broadly applied to heterogeneous and solid-state catalysts – to a subset of highly active Ru-based, homogenous catalysts with Ru(NNN)(NN) or similar structures. Consideration of an additional ET/PCET event prior to O-O bond formation – necessary to yield the Ru^V^ states is added in the analysis. These modifications reveal insights into the ideal range of relative energies of the Ru^IV^=O and Ru^IV^-OH intermediates, as well as a narrow region of optimal change in free energy of WNA on Ru^V^ reactions: ΔG ~> −0.1eV. Finally, a narrow range of Ru^IV^-OH to Ru^V^=O redox potentials (~1.28V) corresponds to highest reported oxygen-evolution activity. These findings are based on empirical data – oxygen evolution rates and cyclic voltammetry redox couples – absent in prior Sabatier-principle based works on similar families of catalysts.

## 2. Results

A schematic representation of the complexes considered in this study are shown below in Figure 1. Included in the set of model catalysts are those with neutral and negatively charged ligands. Each catalyst studied may be considered a deviation of another; either by substitution of an R group or similar modification can one catalyst be related to at least one other. This allows for comparisons of the precise effect of the ligand structure on catalytic activity and predictor relationships; these catalysts are chosen to enable both broad study of the Ru(NNN)(NN), Ru(NNN)(NO), and similarly structured families, as well as more narrow-scoped considerations of electronic structure effects. Table S1 shows the oxygen evolution rates as determined for each of the catalysts studied. The oxygen-evolution rate of the bda-type complexes is not considered for our analysis, as Ru(bda)(4-pic)_2_ and Ru(bda)(isoq)_2_ have second-order rate with catalyst concentration [51], unlike the other catalysts studied – first-order with catalyst concentration.

The twelve catalysts were modelled in each of the seven states of a typical WNA mechanism outlined in Figure 2. Each geometry was optimized, and free energies of each state were determined for each catalyst; see Methods section for computational details. The results of the DFT optimization and energy computation are reported in Tables S2–S4: the free energies of each intermediate, computed redox potentials, and changes in free energy of WNA processes. With such a large set of catalysts and species in our data set, we must validate our choice of basis set and computational methodology. This is done via comparison of computed and experimental redox potentials, Table 1. Due to systematic error inherent to DFT methods, agreement with experimental redox couples is ideally near/with ~0.2V agreement, the generally accepted margin of error for computed disagreement using similar basis set and computational methods [13,39,41,49,52]. However, due to the large number of catalysts and experimental evidence, some computed couples may have slightly larger differences; prominent examples of this include the Ru^II^/Ru^III^ couples of the structurally similar Ru(EtOtpy)(bpy) and Ru(tpy)(bpy) catalysts. Both are predicted to advance to Ru^III^ via a PCET reaction. Computation assigns a redox potential nearly 0.3V lower for these couples. However, the computed ET redox reaction for Ru(EtOtpy)(bpy) predicts a 1.21V potential for Ru^II^/Ru^III^, in closer agreement with experiment – 0.98V. Computational prediction is equally dissimilar to the reported 1.04V potential for Ru(tpy)(bpy) – both are nearly 0.3V different (Tables 1 and S3).

Table 1 cites experimentally reported redox potentials alongside computed redox couples for ET and PCET paths. Most of the computed redox potentials are within ~0.2V of the reported redox couples. This serves to validate the choice of basis set and the use of the generalized gradient approximation methodology, B3LYP, used in this study. Table 1 reports computational assignment of the specific redox couple at pH=0 for each catalyst’s redox mechanism. Differences in the redox mechanism within catalyst of (NNN)(NN) structure suggest that specific ligand modifications sufficiently alter the electronic structure to overcome the effect of the inner coordination sphere’s influence. Note that some computed redox couples, such as the Ru^IV^/Ru^V^ of Ru(bda)(isoq)_2_, differ from proposed mechanism in identifying PCET/ET steps (Table 1 and S3) [49].

**Figure 3.**
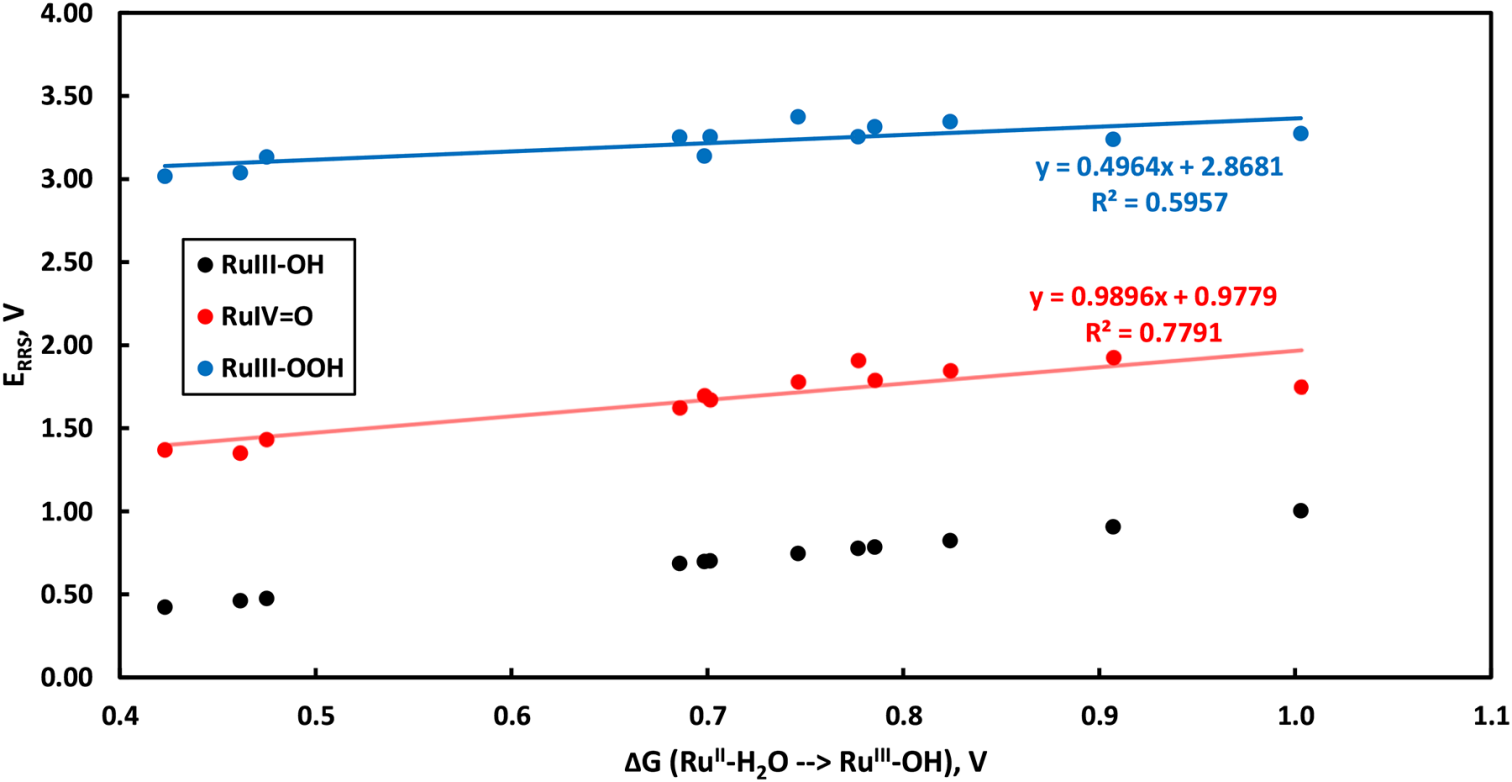
Free energy scaling relationships among the E_RRS_ of Ru^III^-OH, Ru^IV^=O, and Ru^III^-OOH for the twelve molecular catalysts considered in this study.

Having validated our choice of basis set and computational methodology, we proceeded with our adaption of conventional LFESR analysis to our set of homogenous, Ru-based catalysts outlined in Figure 1. Following prior methodologies for LFESR analysis [62], computed energies of the Ru^III^-OH, Ru^IV^=O, and Ru^III^-OOH intermediates (Table S5) relative to the stable Ru^II^ species for each catalyst; these energies relative to the rest state (E_RRS_) are plotted against the representative parameter of the redox potential of the PCET Ru^II^-H_2_O to Ru^III^-OH reaction in Figure 3.

Essential in the construction of volcano plots is establishing LFESRs, relating energetics of different catalytic intermediates onto a single variable [34]. In principle, different intermediates’ stabilities are interrelated and cannot be significantly altered independently. Should a set of LFESRs be established for a particular intermediate, mathematical relationships derived therein may describe the entire energetic landscape in terms of a single descriptor. Plots of the (negative) change in free energy along the potential-determining step of a mechanism against a “descriptor” variable – for which the existence of LFESRs contain information on each catalyst’s entire energetic landscape – indicate “ideal” catalysts; this is a volcano plot [34].

The free energy scaling relationships between the redox reaction of Ru^II^-H_2_O to Ru^III^-OH are moderately linear with the E_RRS_ of Ru^IV^=O but do not have a strong linear relationship with the E_RRS_ of the Ru^III^-OOH species resulting from WNA on Ru-oxo species (Figure 3). Reports of LFESR-based analysis on catalytic systems often produce volcano plots using regressions with a high value for r^2^, usually above or near 0.90 [62,63]. Few analyses consider LFESR methods below r^2^ of 0.8 [64]– which we will treat as a cutoff for sufficiently linear of a correlation. Reselection of the descriptor (x-axis variable in Figure 3) does not result in sufficiently strong correlations required for LFESR analysis; Tables S6 and S7 show the complete set of correlations of each descriptor against each E_RRS_. Though some descriptors, such as the Ru^III^-H_2_O to Ru^IV^-OH PCET reaction and the Ru^IV^=O to Ru^V^=O ET reaction, correlate well with early stages in the mechanism, they correlate more poorly with the WNA step. This E_RRS_ for the Ru^III^-OOH intermediate does not correlate strongly with any descriptor studied (Tables S6 and S7). This step tends to have the weakest correlations with the descriptor variables, and its strongest correlation is with the Ru^II^-H_2_O PCET step, already dismissed above (Figure 3). As such, these results indicate that this approach to LFESR analysis is inappropriate for the set of catalysts chosen; however, we will continue our study with Sabatier-principle and volcano-plot based analysis using empirical data of oxygen evolution rates – an approach which, to the best of our knowledge, is unpublished.

**Figure 4.**
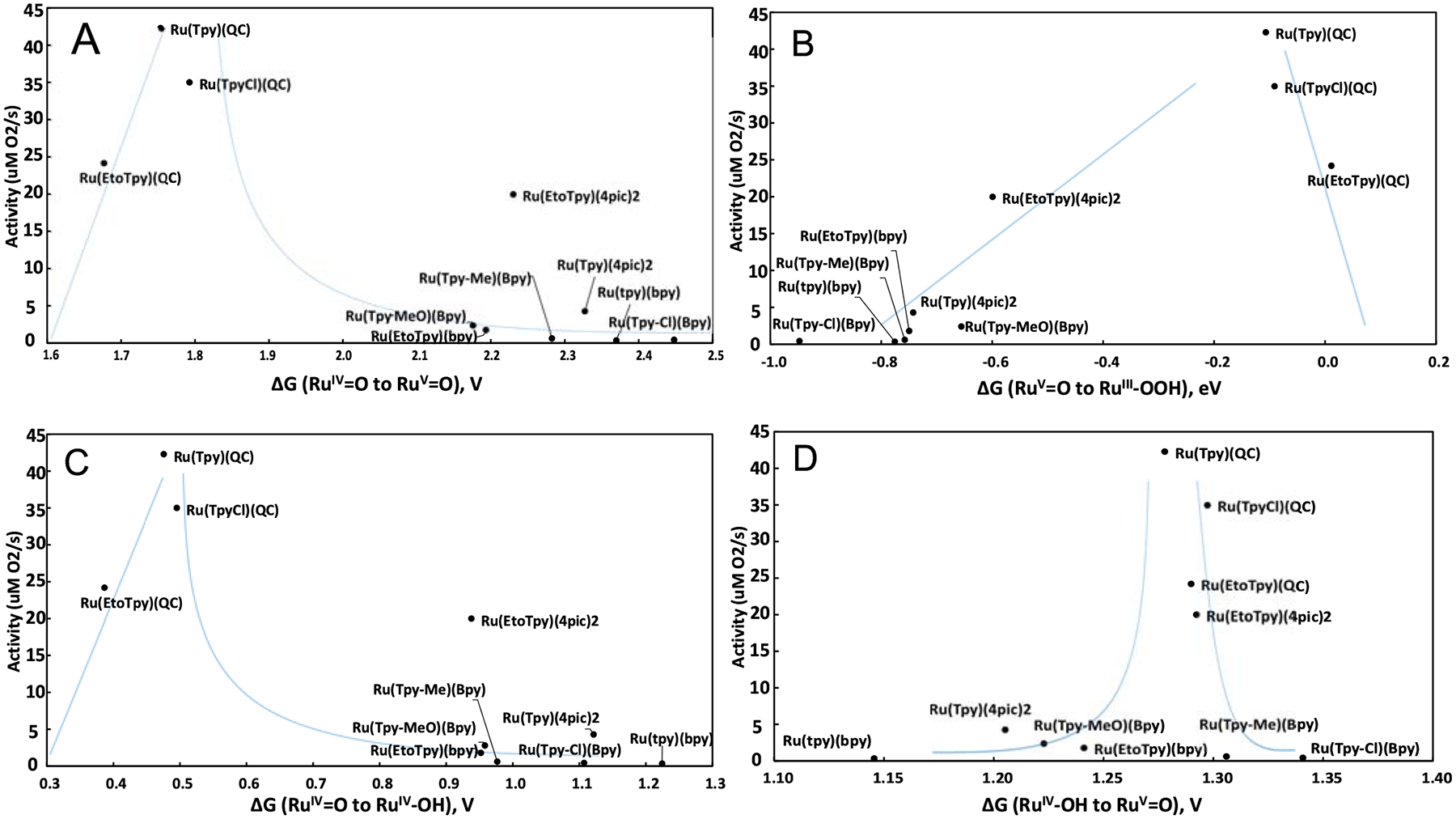
Plots of activity over computed A) potential of conventional Ru^IV^=O to Ru^V^=O ET redox reaction, B) change in free energy of WNA on Ru^V^=O, C) change in free energy of the Ru^IV^=O to Ru^IV^-OH protonation reaction, and D) redox potential of the PCET Ru^IV^-OH to Ru^V^=O reaction.

To continue our analysis, we consider alternative volcano plot / Sabatier principle-based analysis, using known oxygen-evolution rates as an experimentally derived measure of the quality of each catalyst. This approach to analysis continues in parallel with conventional LFESR-based analysis yielding volcano plots, using η_th_ as the sole, theoretical analogue of catalytic effectiveness and quality. Considering still the utility for Ru-based WOCs to access the high-valent Ru^V^=O intermediate prior to WNA and O-O bond formation, our volcano plot and Sabatier principle-based analysis focuses on four late-stage steps along the WNA cycle: Ru^IV^-OH to Ru^V^=O, Ru^IV^=O to Ru^V^=O, Ru^IV^=O to Ru^IV^-OH, WNA on and Ru^V^=O. Figure 4 shows the volcano-like plots produced from this approach.

Each graph in Figure 4 identifies a region of ideal catalytic activity based on a band of relative energies of intermediates in the WNA mechanism. Figure 4A explores a Sabatier-based relationship between the Ru^IV^=O to Ru^V^=O ET reaction often cited as needed for O-O bond formation – and oxygen evolution activity; easier access to the high-valent Ru^V^ intermediate suggests high activity in O_2_ evolution. Figure 4B supports the necessity of reaching Ru^V^=O prior to WNA reaction allowing for negative ΔG for the O-O bond formation. However, the catalysts with highest oxygen evolution rates are observed to experience a minimally downhill reaction in forming O-O bonds – ΔG ~> −0.1eV. Figure 4C suggests that ideal Ru-based catalysts have only a slightly thermodynamically uphill protonation from Ru^IV^=O to Ru^IV^-OH; unsurprisingly, this is similar to what is seen in Figure 4A. Figure 4D identifies a very narrow range of energies of the PCET Ru^IV^-OH to Ru^V^=O reaction, centered ~1.28V, where the four most active catalysts reside, surrounded by lower activity catalysts; the catalysts with lowest activity are located farthest from this region.

Having considered LFESR and Sabatier principle-based analysis for relationships with empirical oxygen evolution activity, our last approach attempts to identify linear regressions of various descriptors against oxygen evolution activity. The predictors in this approach consist of parameters specific to the species late in the WNA cycle – Ru^IV^, Ru^V^, and peroxo species. Table S8 reports the correlations of numerous computed descriptor variables against the experimentally reported oxygen-evolution activity and the theoretical overpotential of the catalytic system, Equation 6. Tables S9–S13 report the values for each of the parameters reported in Table S8. Charge and spin densities refer to the Milliken charge and spin densities as calculated by Gaussian16 [65]. Note that while computation of the Mulliken spin and density populations highly dependent upon basis set selection [66], the basis set is kept constant in this work. As such, the systematic bias inherent to basis set selection should not impact regression analysis. The four redox potentials / changes in free energies (Ru^IV^-OH to Ru^V^=O, Ru^IV^=O to Ru^V^=O, Ru^IV^=O to Ru^IV^-OH, WNA on Ru^V^=O) are discussed in greater depth later, as they suggest a Sabatier-like relationship with activity rather than with a linear regression-based model. The theoretical overpotential for most catalysts was taken as the very demanding ET reaction of Ru^IV^=O to Ru^V^=O proposed by most works. Thus, we consider only the strongest correlated descriptors of the catalytic mechanisms, the charge densities of the atoms most central to the Ru atom in the peroxide Ru^III^-OOH state. The charge densities on Ru and the adjacent oxygen atom are strongly inversely correlated with oxygen evolution activity and, suitably, by theoretical overpotential. Though some recent work [67,68] has been done on using linear regression-based methods to find suitable correlators with catalytic activity, a substantially greater amount of work uses Sabatier principle-motivated methods. Since most of the strongest correlators with activity are also discussed at length in the Sabatier-principle-based analysis, said analysis will compose the bulk of the discussion.

## 3. Discussion

Related to the employment of linear scaling relationships to find correlations and predictors of catalytic activity is the use of volcano plots, themselves rooted in Sabatier’s principle. Sabatier’s principle states that a catalyst should bind a substrate (water, or oxo group) neither too strongly nor too weakly. Volcano plots may predict catalytic performance – via overpotential or other thermodynamic/kinetic considerations – by consideration of a descriptor variable and linear free energy scaling relationships. Herein, this descriptor is related to estimate relative energies of other intermediates and states and, with processing, may lead to a “volcano” curve identifying predicted catalytic performance, with ideal catalyst candidates at the peak of the curve [17,26,28].

In 2018, Busch *et al*. [62] demonstrate the use of linear free energy scaling relationships to assess the viability of solid state and molecular OER catalysts, using a variety DFT-based functionals. The work included specifically use of generalized gradient approximation functionals (GGAs) and meta-GGA functionals. Using eight model catalysts, composed of a corrole ligand or a porphyrin equivalent, centered about a transition metal atom, Busch et al. were able to produce LFESRs among the E_RRS_ of (* represents transition metal atom) *-OH, *=O, and *-OOH for various levels of theory for these model catalysts. From these LFESRs, Busch *et al*. were able to describe the thermodynamics of the WNA mechanism in relation to the overpotential of the potential-determining step; thus, they were able to determine a minimal systematic overpotential in terms of a representative parameter of these systems ---the E_RRS_ energies of *-OH. The work did not consider mechanisms involving metal center in the formal oxidation state of (V) such as Ru^V^ or Fe^V^ intermediates.

We extended this LFESR-based approach to this broader group of catalysts with less ligand symmetry and, by extension, more ligand variability. The E_RRS_ of the Ru^III^-OH, Ru^IV^=O, and Ru^III^-OOH intermediates are plotted against the representative parameter of the redox potential of the PCET Ru^II^-H_2_O to Ru^III^-OH reaction in Figure 3. These are based on the computationally derived energetics (Tables S3–S5). Regression against the Ru^II^ PCET redox potential yielded r^2^ of 0.78 and 0.60 for the E_RRS_ of Ru^IV^=O and Ru^III^-OOH relative to the initial Ru^II^-H_2_O state. Other descriptor variables were considered to determine the existence of any sufficiently robust LFESRs (Tables S6 and S7), using the cutoff of r^2^ near/above 0.80 as a lower bound of correlation. While some modest correlations were discovered, no mechanism could be constructed from pathways adequately linearly correlated against any individual predictor. As such, conventional LFESR analysis against E_RRS_ is inappropriate for a wholly Ru-based system with even somewhat variable ligand structure. One report which employed such a technique in the past considered ligand structures exclusively a perfluoroporphyrin and corrole ligands centered on a transition metal atom [62]. This suggests that ligand differences are more highly impactful to the energetics of different intermediates; such differences are enough to change the catalytic activity by over two orders of magnitude, from Ru(tpy-Cl)(bpy) to Ru(tpy)(QC) and a 5x difference with substitution of an electron-donating EtO group in the even more structurally similar Ru(tpy)(4-pic)_2_ to Ru(EtOtpy)(4-pic)_2_.

In 2019, Craig *et al*. [17] expand upon the groundwork established by Busch *et al*. prior. Craig and coworkers consider seventeen molecular OER catalysts based on a variety of transition metals (Ru, Mn, Fe, Co, Ni, and Cu); ruthenium acts at the metal center in nine of these seventeen catalysts. They find that unmodified use of conventional scaling relations for heterogeneous systems does not accurately predict the catalytic activity of a set of homogenous catalysts; typical OER scaling relations addressing the OER for heterogenous systems neglects the ET reaction from Ru^IV^=O (or the PCET reaction from Ru^IV^-OH) yielding Ru^V^=O prior to WNA and O-O bond formation – Craig *et al*. briefly explore this by exploring the Ru^IV^=O to Ru^V^=O descriptor, discovering approximately half (five of nine) of their Ru-based catalysts – including Ru(bda)(4-pic)_2_ and Ru(bda)(isoq)_2_ – undergo ET for this reaction and have η_th_ defined by this potential-limiting step. As mentioned previously, many Ru-based WOCs require access to Ru^V^ prior to formation of an O-O bond since WNA processes on Ru^IV^=O tend to be thermodynamically unfavorable. Numerous catalytic reports indicate catalytic systems based on other elements, are predicted to form O-O bonds via WNA on Metal^IV^=O species [69–71]. As such, that our work focuses more on high-valent redox and WNA reactions is appropriate given our exclusive focus on the Ru metal center.

Figure 4C shows the relationships between the oxygen-evolving activity and the protonation energy of Ru^IV^=O, often necessary for formation of the Ru^IV^-OH needed for PCET to Ru^V^. Most of the highly active catalysts show a similar trend, where a lower protonation potential to Ru^IV^-OH relates to higher oxygen-evolving activity. An exception to this trend is the Ru(EtO-tpy)(4-pic)_2_ catalyst, found at 0.94V. This exception to the Sabatier-like trend suggests that catalytic dependence on this process is not as strong as the other relationships tested. Note that the Ru(tpy)(4-pic)_2_ catalyst – most structurally like Ru(EtO-tpy)(4-pic)_2_ – has a greater protonation activity alongside a lower oxygen evolution rate. A similar trend exists for the Ru(tpy-R)(bpy) family of catalysts as well. Taken as a whole, these data indicate that should Ru^IV^-OH be sufficiently accessible, oxygen evolution activity is not inhibited.

Figure 4D indicates that a PCET redox potential of approximately 1.28V between Ru^IV^/Ru^V^ is achievable and ideal for oxygen evolving activity. This region is surrounded by immediate and steep binding slopes lead to overly strong or weak catalyst-substrate interactions, inhibiting oxygen evolution activity. That Ru(tpy-Cl)(bpy) is weakly bound in Ru^IV^-OH is supported by energetics shown in Tables S3 and S5. This ideal binding region indicates that catalysts of the Ru(NNN)(NN) sand similar ligand structures may have facile access to the catalytically active Ru^V^ species with a monotonically increasing series of redox potentials: Ru^II^/Ru^III^, Ru^III^/Ru^IV^, and Ru^IV^/Ru^V^. Figure 4B shows a similar result; oxygen-evolution activity may be inhibited by Ru^V^ states which are too weakly bound. This characterizes most of the catalysts studied, excepting those with the highest catalytic activity, the Ru(tpy-R)(QC) family of catalysts. Ru(EtO-tpy)(4-pic)_2_ offers only slightly weaker oxygen evolution activity and can be located partway along this “weakly binding slope” between the high and low activity regimes. Simply, in the absence of transition state energy considerations, the most catalytically active catalysts have minutely downhill energetics via WNA processes: ΔG ~> −0.1eV, implying a lower-energy, more accessible Ru^V^=O intermediate.

## 5. Materials and Methods

### General Information:

All chemicals and solvents were purchased from Sigma Aldrich, AK Scientific and TCI America and used as received. Aqueous solutions were prepared using ultrapure (Type 1) water (resistivity 18.2 MΩ·cm at 25 °C) from a Q-POD unit of Milli-Q integral water purification system (Millipore, Billerica, MA, USA). Solvents and chemicals were purchased from Sigma-Aldrich (St. Louis, MO, USA) and were used without further purification.

### O_2_ Evolution:

Oxygen evolution was measured with a Clark-type polarographic oxygen electrode with an Oxygraph System (Hansatech Instruments Ltd., King’s Lynn, Norfolk, UK). Calibration was performed by measuring signal in O_2_-saturated deionized water and then adding sodium dithionite (an oxygen-depleting agent). The drop in the signal was set equal to the solubility of oxygen in water at room temperature (262 μmol/L). In Chemical catalysis the borosilicate vessel was filled with 500 μL solutions of the complex at pH = 1 (in 0.1 M nitric acid) and was constantly stirred. 20 mM. of Ce^IV^ dissolved in nitric acid at pH = 1 was added to the chamber and oxygen concentration was recorded as a function of time.

### DFT Calculation:

Density functional theory calculations were performed at the B3LYP level of theory, with the DGDZVP basis set for the Ru and Cl atoms, and the 6–31G* basis set was used for light (C, H, N, O) atoms. All molecules were modelled in water using the Conductor Polarized Continuum Model (CPCM) solvation model. All redox potentials were calculated using the DFT-calculated free energies of the products minus the reactants. From this value, 4.44 V was subtracted to account for the NHE voltage. The free energy of solvation for H+ was taken to be −11.64 eV. Geometries of intermediates are optimized, then electronic/thermal energies and vibrational frequencies are calculated as single-point calculations upon the optimized states. Optimization of some geometries required use of quadratic convergence criteria. Implicit consideration of water molecule in WNA methods with use of free energy of water molecule computed in 6–31G* at the B3LYP level of theory.

### Pearson Coefficient:

The linear relationship between the two variables on a scatter plot is strongest when the points are closer to lying on a straight line. The Pearson coefficient *r* quantifies the strength of this linear relationship. For two variables *x* and *y* and data taken in *n* pairs,

(7) $$\left\{\left[{\text{x}}_{1},{\text{y}}_{1}\right],\left[{\text{x}}_{2},{\text{y}}_{2}\right],\ldots \left[{\text{x}}_{\text{n}},{\text{y}}_{\text{n}}\right]\right\},$$

Othe Pearson correlation coefficient is given by: Pearson correlation coefficient is given by:

(8) $$r=\frac{\sum_{i=1}^{n}\left(x_{i}-\bar{x}\right)\left(y_{i}-\bar{y}\right)}{\sqrt{\sum_{i=1}^{n}{\left(x_{i}-\bar{x}\right)}^{2}\sum_{i=1}^{n}{\left(y_{i}-\bar{y}\right)}^{2}}},$$

wherwhere $\bar{x}$ and $\bar{y}$ represent the means of the *x* and *y* variables, respectively. The strongest correlations have magnitudes of *r* closer to 1, and the value of the correlation coefficient is always between −1 and +1. Positive values indicate positive linear relationships; negative values represent negative linear relationships.

## Supplementary Material

DFT Coordinates

Supporting Information

## Figures and Tables

**Table 1.** Computed Redox potentials for pH=0 for each of the twelve catalysts ET as well as PCET paths are considered. Experimental redox potentials are located beneath computed redox potentials. “No Data” signifies that no experimentally derived couples have been reported.

	RuII/RuIII		RuIII/RuIV				RuIV/RuV	
	*II-H2O / III-H2O*	*II-H2O / III-OH*	*III-H2O / IV-OH*	*III-H2O / IV=O*	*III-OH / IV-OH*	*III-OH / IV=O*	*IV-OH / V=O*	*IV=O / V=O*
**Ru(EtoTpy)(4pic)2**	1.30	0.69	1.26	0.32	1.87	0.94	1.29	2.23
	0.97V [41]							
**Ru(Tpy)(4pic)2**	1.38	0.70	1.42	0.30	2.09	0.97	1.21	2.33
	1.00V [41]							
**Ru(tpy)(bpy)**	1.32	0.75	1.68	0.46	2.26	1.03	1.15	2.37
	1.04V [53]		1.23V [53], 1.39V [54]				1.60V [55], 1.73V [56], 1.80V [57]	
**Ru(EtoTpy)(bpy)**	1.21	0.70	1.44	0.48	1.95	1.00	1.24	2.19
	0.98V [58]		1.24V [58]					
**Ru(TpyCl)(QC)**	0.68	0.47	1.25	0.75	1.45	0.96	1.30	1.79
	0.61V [59]							
**Ru(EtoTpy)(QC)**	0.59	0.46	1.14	0.76	1.27	0.89	1.29	1.68
	0.63V [59]		1.19V [59]				1.73V [59]	
**Ru(Tpy)(QC)**	0.62	0.42	1.22	0.75	1.42	0.95	1.28	1.75
	0.67V [60], 0.82V [59]		1.20V [60], 1.36V [59]				1.62 [60], 1.75V [59]	
**Ru(Tpy-MeO)(Bpy)**	1.23	1.00	1.48	0.52	1.70	0.74	1.22	2.18
	No Data							
**Ru(Tpy-Me)(Bpy)**	1.26	0.79	1.50	0.53	1.98	1.00	1.31	2.28
	No Data							
**Ru(Tpy-Cl)(Bpy)**	1.40	0.82	1.56	0.45	2.13	1.02	1.34	2.45
	No Data							
**Ru(bda)(isoq)2**	0.19	0.91	2.08	1.74	1.36	1.02	1.03	1.38
	0.63V [51]		1.09V [51]				1.27V [51]	
**Ru(bda)(4pic)2**	0.09	0.78	0.98	1.82	0.30	1.13	2.12	1.28
	0.66V [61]		1.15V [61]				1.35V [61]	

## Data Availability

Refer to Supplementary Materials to access data supporting results.

## References

[1] Lewis NS Research Opportunities to Advance Solar Energy Utilization. Science (80-.) 2016, 351 (6271), aad1920. 10.1126/science.aad1920 26798020

[2] Gust D; Moore TA; Moore AL Solar Fuels via Artificial Photosynthesis. Acc. Chem. Res 2009, 42 (12), 1890–1898. 19902921 10.1021/ar900209b

[3] Song W; Chen Z; Brennaman MK; Concepcion JJ; Patrocinio AOT; Iha NYM; Meyer TJ Making Solar Fuels by Artificial Photosynthesis. Pure Appl. Chem 2011, 83 (4), 749–768.

[4] Tachibana Y; Vayssieres L; Durrant JR Artificial Photosynthesis for Solar Water-Splitting. Nat. Photonics 2012, 6 (8), 511–518.

[5] Lewis NS; Nocera DG Powering the Planet: Chemical Challenges in Solar Energy Utilization. Proc. Natl. Acad. Sci 2006, 103 (43), 15729–15735. 17043226 10.1073/pnas.0603395103 PMC1635072

[6] Nocera DG Solar Fuels and Solar Chemicals Industry. Acc. Chem. Res 2017, 50 (3), 616–619. 28945407 10.1021/acs.accounts.6b00615

[7] Nocera DG The Artificial Leaf. Acc. Chem. Res 2012, 45 (5), 767–776. 10.1021/ar2003013. 22475039

[8] Kim W; Edri E; Frei H Hierarchical Inorganic Assemblies for Artificial Photosynthesis. Acc. Chem. Res 2016, 49 (9), 1634–1645. 27575376 10.1021/acs.accounts.6b00182

[9] Young KJ; Martini LA; Milot RL; Snoeberger III RC; Batista VS; Schmuttenmaer CA; Crabtree RH; Brudvig GW Light-Driven Water Oxidation for Solar Fuels. Coord. Chem. Rev 2012, 256 (21–22), 2503–2520. 25364029 10.1016/j.ccr.2012.03.031 PMC4214930

[10] Karkas MD; Verho O; Johnston EV; Åkermark B Artificial Photosynthesis: Molecular Systems for Catalytic Water Oxidation. Chem. Rev 2014, 114 (24), 11863–12001. 25354019 10.1021/cr400572f

[11] Blakemore JD; Crabtree RH; Brudvig GW Molecular Catalysts for Water Oxidation. Chem. Rev 2015, 115 (23), 12974–13005. 26151088 10.1021/acs.chemrev.5b00122

[12] Sivula K; Van De Krol R Semiconducting Materials for Photoelectrochemical Energy Conversion. Nat. Rev. Mater 2016, 1 (2), 1–16.

[13] Ravari AK; Zhu G; Ezhov R; Pineda-Galvan Y; Page A; Weinschenk W; Yan L; Pushkar Y Unraveling the Mechanism of Catalytic Water Oxidation via de Novo Synthesis of Reactive Intermediate. J. Am. Chem. Soc 2019, 142 (2), 884–893. 10.1021/jacs.9b10265 31865704

[14] Sheridan M; Sherman BD; Xie Y; Wang Y Heterogeneous Water Oxidation Catalysts for Molecular Anodes and Photoanodes. Sol. RRL 2021, 5 (6), 2000565.

[15] Ye S; Ding C; Liu M; Wang A; Huang Q; Li C Water Oxidation Catalysts for Artificial Photosynthesis. Adv. Mater 2019, 31 (50), 1902069. 10.1002/adma.201902069 31495962

[16] Thalluri SM; Bai L; Lv C; Huang Z; Hu X; Liu L Strategies for Semiconductor/Electrocatalyst Coupling toward Solar-driven Water Splitting. Adv. Sci 2020, 7 (6), 1902102. 10.1002/advs.201902102 PMC7080548 32195077

[17] Craig MJ; Coulter G; Dolan E; Soriano-López J; Mates-Torres E; Schmitt W; García-Melchor M Universal Scaling Relations for the Rational Design of Molecular Water Oxidation Catalysts with Near-Zero Overpotential. Nat. Commun 2019, 10 (1), 1–9. 31704927 10.1038/s41467-019-12994-w PMC6841662

[18] Hessels J; Detz RJ; Koper MTM; Reek JNH Rational Design Rules for Molecular Water Oxidation Catalysts Based on Scaling Relationships. Chem. Eur. J 2017, 23 (65), 16413–16418. 28836700 10.1002/chem.201702850

[19] Schilling M; Böhler M; Luber S Towards the Rational Design of the Py5-Ligand Framework for Ruthenium-Based Water Oxidation Catalysts. Dalt. Trans 2018, 47 (31), 10480–10490. 10.1039/c8dt01209a 29780997

[20] Huang J; Zhang Y; Ding Y Rationally Designed/Constructed CoO x/WO3 Anode for Efficient Photoelectrochemical Water Oxidation. Acs Catal. 2017, 7 (3), 1841–1845.

[21] Gao J; Huang X; Cai W; Wang Q; Jia C; Liu B Rational Design of an Iridium–Tungsten Composite with an Iridium-Rich Surface for Acidic Water Oxidation. ACS Appl. Mater. Interfaces 2020, 12 (23), 25991–26001. 32428393 10.1021/acsami.0c05906

[22] Latimer AA; Kulkarni AR; Aljama H; Montoya JH; Yoo JS; Tsai C; Abild-Pedersen F; Studt F; Nørskov JK Understanding Trends in C–H Bond Activation in Heterogeneous Catalysis. Nat. Mater 2017, 16 (2), 225–229. 27723737 10.1038/nmat4760

[23] Liao P; Getman RB; Snurr RQ Optimizing Open Iron Sites in Metal–Organic Frameworks for Ethane Oxidation: A First-Principles Study. ACS Appl. Mater. Interfaces 2017, 9 (39), 33484–33492. 28394564 10.1021/acsami.7b02195

[24] Yang X-F; Wang A; Qiao B; Li J; Liu J; Zhang T Single-Atom Catalysts: A New Frontier in Heterogeneous Catalysis. Acc. Chem. Res 2013, 46 (8), 1740–1748. 23815772 10.1021/ar300361m

[25] Flytzani-Stephanopoulos M Gold Atoms Stabilized on Various Supports Catalyze the Water–Gas Shift Reaction. Acc. Chem. Res 2014, 47 (3), 783–792. 24266870 10.1021/ar4001845

[26] Abild-Pedersen F; Greeley J; Studt F; Rossmeisl J; Munter TR; Moses PG; Skulason E; Bligaard T; Nørskov JK Scaling Properties of Adsorption Energies for Hydrogen-Containing Molecules on Transition-Metal Surfaces. Phys. Rev. Lett 2007, 99 (1), 16105. 10.1103/PhysRevLett.99.016105 17678168

[27] Nørskov JK; Bligaard T; Rossmeisl J; Christensen CH Towards the Computational Design of Solid Catalysts. Nat. Chem 2009, 1 (1), 37–46. 21378799 10.1038/nchem.121

[28] Wodrich MD; Sawatlon B; Busch M; Corminboeuf C The Genesis of Molecular Volcano Plots. Acc. Chem. Res 2021, 54 (5), 1107–1117. 33570407 10.1021/acs.accounts.0c00857

[29] Cordova M; Wodrich MD; Meyer B; Sawatlon B; Corminboeuf C Data-Driven Advancement of Homogeneous Nickel Catalyst Activity for Aryl Ether Cleavage. Acs Catal. 2020, 10 (13), 7021–7031.

[30] Busch M; Wodrich MD; Corminboeuf C Linear Scaling Relationships and Volcano Plots in Homogeneous Catalysis–Revisiting the Suzuki Reaction. Chem. Sci 2015, 6 (12), 6754–6761. 28757966 10.1039/c5sc02910d PMC5508671

[31] Mori-Sánchez P; Cohen AJ; Yang W Many-Electron Self-Interaction Error in Approximate Density Functionals. J. Chem. Phys 2006, 125 (20), 201102. 17144681 10.1063/1.2403848

[32] Ruzsinszky A; Perdew JP; Csonka GI; Vydrov OA; Scuseria GE Density Functionals That Are One-and Two-Are Not Always Many-Electron Self-Interaction-Free, as Shown for H 2+, He 2+, Li H+, and Ne 2+. J. Chem. Phys 2007, 126 (10), 104102. 17362056 10.1063/1.2566637

[33] Haunschild R; Henderson TM; Jimenez-Hoyos CA; Scuseria GE Many-Electron Self-Interaction and Spin Polarization Errors in Local Hybrid Density Functionals. J. Chem. Phys 2010, 133 (13), 134116. 20942532 10.1063/1.3478534

[34] Wodrich MD; Busch M; Corminboeuf C Accessing and Predicting the Kinetic Profiles of Homogeneous Catalysts from Volcano Plots. Chem. Sci 2016, 7 (9), 5723–5735. 30034712 10.1039/c6sc01660j PMC6022257

[35] Craig MJ; García-Melchor M High-Throughput Screening and Rational Design to Drive Discovery in Molecular Water Oxidation Catalysis. Cell Reports Phys. Sci 2021, 2 (7), 100492.

[36] Galán-Mascarós JR Water Oxidation at Electrodes Modified with Earth-abundant Transition-metal Catalysts. ChemElectroChem 2015, 2 (1), 37–50.

[37] Smith PF; Kaplan C; Sheats JE; Robinson DM; McCool NS; Mezle N; Dismukes GC What Determines Catalyst Functionality in Molecular Water Oxidation? Dependence on Ligands and Metal Nuclearity in Cobalt Clusters. Inorg. Chem 2014, 53 (4), 2113–2121. 24498959 10.1021/ic402720p

[38] Moonshiram D; Alperovich I; Concepcion JJ; Meyer TJ; Pushkar Y Experimental Demonstration of Radicaloid Character in a RuV= O Intermediate in Catalytic Water Oxidation. Proc. Natl. Acad. Sci 2013, 110 (10), 3765–3770. 23417296 10.1073/pnas.1222102110 PMC3593827

[39] Moonshiram D; Pineda-Galvan Y; Erdman D; Palenik M; Zong R; Thummel R; Pushkar Y Uncovering the Role of Oxygen Atom Transfer in Ru-Based Catalytic Water Oxidation. J. Am. Chem. Soc 2016, 138 (48), 15605–15616. 10.1021/jacs.6b08409. 27802032

[40] Ertem MZ; Concepcion JJ Oxygen Atom Transfer as an Alternative Pathway for Oxygen–Oxygen Bond Formation. Inorg. Chem 2020, 59 (9), 5966–5974. 10.1021/acs.inorgchem.9b03751. 32314576

[41] Patel J; Bury G; Ravari AK; Ezhov R; Pushkar Y Systematic Influence of Electronic Modification of Ligands on the Catalytic Rate of Water Oxidation by a Single-Site Ru-Based Catalyst. ChemSusChem 2022. 10.1002/cssc.202101657 PMC10063387 34905663

[42] Matheu R; Garrido-Barros P; Gil-Sepulcre M; Ertem MZ; Sala X; Gimbert-Suriñach C; Llobet A The Development of Molecular Water Oxidation Catalysts. Nat. Rev. Chem 2019, 3 (5), 331–341.

[43] Erdman D; Pineda-Galvan Y; Pushkar Y Mechanistic Analysis of Water Oxidation Catalyst Cis-[Ru (Bpy) 2 (H2O) 2] 2+: Effect of Dimerization. Catalysts 2017, 7 (2), 39.

[44] Ezhov R; Karbakhsh Ravari A; Page A; Pushkar Y Water Oxidation Catalyst Cis-[Ru (Bpy)(5, 5′-Dcbpy)(H2O) 2] 2+ and Its Stabilization in Metal–Organic Framework. ACS Catal. 2020, 10 (9), 5299–5308.

[45] Bucci A; Menendez Rodriguez G; Bellachioma G; Zuccaccia C; Poater A; Cavallo L; Macchioni A An Alternative Reaction Pathway for Iridium-Catalyzed Water Oxidation Driven by Cerium Ammonium Nitrate (Can). ACS Catal. 2016, 6 (7), 4559–4563.

[46] Dengel AC; Griffith WP Studies on Transition-Metal Oxo and Nitrido Complexes. 12. Synthesis, Spectroscopic Properties, and Reactions of Stable Ruthenium (V) and Osmium (V) Oxo Complexes Containing. Alpha.-Hydroxy Carboxylate and. Alpha.-Amino Carboxylate Ligands. Inorg. Chem 1991, 30 (4), 869–871.

[47] Planas N; Vigara L; Cady C; Miró P; Huang P; Hammarstrom L; Styring S; Leidel N; Dau H; Haumann M Electronic Structure of Oxidized Complexes Derived from Cis-[RuII (Bpy) 2 (H2O) 2] 2+ and Its Photoisomerization Mechanism. Inorg. Chem 2011, 50 (21), 11134–11142. 21992177 10.1021/ic201686c

[48] Pineda-Galvan Y; Ravari AK; Shmakov S; Lifshits L; Kaveevivitchai N; Thummel R; Pushkar Y Detection of the Site Protected 7-Coordinate RuV= O Species and Its Chemical Reactivity to Enable Catalytic Water Oxidation. J. Catal 2019, 375, 1–7.

[49] Lebedev D; Pineda-Galvan Y; Tokimaru Y; Fedorov A; Kaeffer N; Copéret C; Pushkar Y The Key RuV=O Intermediate of Site-Isolated Mononuclear Water Oxidation Catalyst Detected by in Situ X-Ray Absorption Spectroscopy. J. Am. Chem. Soc 2018, 140 (1), 451–458. 10.1021/jacs.7b11388. 29219306

[50] Levin N; Casadevall C; Cutsail III GE; Lloret-Fillol J; DeBeer S; Rüdiger O XAS and EPR in Situ Observation of Ru (V) Oxo Intermediate in a Ru Water Oxidation Complex. ChemElectroChem 2022, 9 (3), e202101271. 35874044 10.1002/celc.202101271 PMC9302654

[51] Duan L; Bozoglian F; Mandal S; Stewart B; Privalov T; Llobet A; Sun L A Molecular Ruthenium Catalyst with Water-Oxidation Activity Comparable to That of Photosystem II. Nat. Chem 2012, 4 (5), 418–423. 22522263 10.1038/nchem.1301

[52] Pushkar Y; Moonshiram D; Purohit V; Yan L; Alperovich I Spectroscopic Analysis of Catalytic Water Oxidation by [RuII (Bpy)(Tpy) H2O] 2+ Suggests That RuV= O Is Not a Rate-Limiting Intermediate. J. Am. Chem. Soc 2014, 136 (34), 11938–11945. 25130482 10.1021/ja506586b

[53] Wasylenko DJ; Ganesamoorthy C; Henderson MA; Koivisto BD; Osthoff HD; Berlinguette CP Electronic Modification of the [RuII (Tpy)(Bpy)(OH2)] 2+ Scaffold: Effects on Catalytic Water Oxidation. J. Am. Chem. Soc 2010, 132 (45), 16094–16106. 20977265 10.1021/ja106108y

[54] Takeuchi KJ; Thompson MS; Pipes DW; Meyer TJ Redox and Spectral Properties of Monooxo Polypyridyl Complexes of Ruthenium and Osmium in Aqueous Media. Inorg. Chem 1984, 23 (13), 1845–1851.

[55] Concepcion JJ; Jurss JW; Norris MR; Chen Z; Templeton JL; Meyer TJ Catalytic Water Oxidation by Single-Site Ruthenium Catalysts. Inorg. Chem 2010, 49 (4), 1277–1279. 20058918 10.1021/ic901437e

[56] Wasylenko DJ; Ganesamoorthy C; Koivisto BD; Henderson MA; Berlinguette CP Insight into Water Oxidation by Mononuclear Polypyridyl Ru Catalysts. Inorg. Chem 2010, 49 (5), 2202–2209. 20131861 10.1021/ic902024s

[57] Wasylenko DJ; Ganesamoorthy C; Henderson MA; Berlinguette CP Unraveling the Roles of the Acid Medium, Experimental Probes, and Terminal Oxidant,(NH4) 2 [Ce (NO3) 6], in the Study of a Homogeneous Water Oxidation Catalyst. Inorg. Chem 2011, 50 (8), 3662–3672. 21413748 10.1021/ic2000188

[58] Yagi M; Tajima S; Komi M; Yamazaki H Highly Active and Tunable Catalysts for O 2 Evolution from Water Based on Mononuclear Ruthenium (II) Monoaquo Complexes. Dalt. Trans 2011, 40 (15), 3802–3804. 10.1039/c0dt01826k 21283862

[59] Patel J; Bury G; Pushkar Y Computational and Spectroscopic Study of Ru(Tpy-R)(QC) Complexes. 2022.

[60] Hoque MA; Chowdhury AD; Maji S; Benet-Buchholz J; Ertem MZ; Gimbert-Suriñach C; Lahiri GK; Llobet A Synthesis, Characterization, and Water Oxidation Activity of Isomeric Ru Complexes. Inorg. Chem 2021, 60 (8), 5791–5803. 33829771 10.1021/acs.inorgchem.1c00112

[61] Timmer BJJ; Kravchenko O; Liu T; Zhang B; Sun L Off-Set Interactions of Ruthenium–Bda Type Catalysts for Promoting Water-Splitting Performance. Angew. Chemie 2021, 133 (26), 14625–14632. 10.1002/anie.202101931 PMC8251529 33861495

[62] Busch M; Fabrizio A; Luber S; Hutter J; Corminboeuf C Exploring the Limitation of Molecular Water Oxidation Catalysts. J. Phys. Chem. C 2018, 122 (23), 12404–12412.

[63] Xu X; Xu H; Cheng D Design of High-Performance MoS 2 Edge Supported Single-Metal Atom Bifunctional Catalysts for Overall Water Splitting via a Simple Equation. Nanoscale 2019, 11 (42), 20228–20237. 31621737 10.1039/c9nr06083a

[64] Piqué O; Illas F; Calle-Vallejo F Designing Water Splitting Catalysts Using Rules of Thumb: Advantages, Dangers and Alternatives. Phys. Chem. Chem. Phys 2020, 22 (13), 6797–6803. 32167118 10.1039/d0cp00896f

[65] Frisch MJ; Trucks GW; Schlegel HB; Scuseria GE; Robb MA; Cheeseman JR; Scalmani G; Barone V; Petersson GA; Nakatsuji H Gaussian 16. Gaussian, Inc. Wallingford, CT 2016.

[66] Govindasamy P; Gunasekaran S; Srinivasan S Molecular Geometry, Conformational, Vibrational Spectroscopic, Molecular Orbital and Mulliken Charge Analysis of 2-Acetoxybenzoic Acid. Spectrochim. Acta Part A Mol. Biomol. Spectrosc 2014, 130, 329–336. 10.1016/j.saa.2014.03.056 24793483

[67] Du X; Zhang X; Yang Z; Gong Y Water Oxidation Catalysis Beginning with CuCo2S4: Investigation of the True Electrochemically Driven Catalyst. Chem. Asian J 2018, 13 (3), 266–270. 29314624 10.1002/asia.201701684

[68] Shi J; Guo Y; Xie F; Chen Q; Zhang M Redox-Active Ligand Assisted Catalytic Water Oxidation by a RuIV= O Intermediate. Angew. Chemie 2020, 132 (10), 4029–4037. 10.1002/anie.201910614 31880387

[69] Ezhov R; Ravari A; Bury G; Smith P; Pushkar Y Formation of CoIV= O Intermediate at the Boundary of the “Oxo-Wall” Induces Water Oxidation. 2020.

[70] Siegbahn PEM Theoretical Studies of O− O Bond Formation in Photosystem II. Inorg. Chem 2008, 47 (6), 1779–1786. 18330969 10.1021/ic7012057

[71] Coggins MK; Zhang M; Chen Z; Song N; Meyer TJ Single-Site Copper (II) Water Oxidation Electrocatalysis: Rate Enhancements with HPO42− as a Proton Acceptor at PH 8. Angew. Chemie Int. Ed 2014, 53 (45), 12226–12230. 10.1002/anie.201407131 25243584

