# Supplementary material for: Computational Analysis of Structure – Activity Relationships in Highly Active Homogeneous Ruthenium-based Water Oxidation Catalysts
Source: Catalysts. Author manuscript; Available in PMC 2023 Jun 12. (PMC10260203; doi:10.3390/catal12080863)
Supplement: DFT Coordinates [file NIHMS1854369-supplement-DFT_Coordinates.pdf]

$Ru^{II}(H_2O)(Bda)(Isoq)_2$

Ru	-0.08397600	0.22270900	-0.36831700
O	-0.56936400	2.11917800	-1.26923400
O	0.85627900	-3.01479100	-1.11907100
O	-0.60855700	-4.29294900	0.03131800
O	-1.03449200	4.23842200	-0.65062300
N	2.03301100	0.51857500	-0.71510600
N	-2.19758800	-0.09772900	-0.20674300
N	0.38244800	-1.20862000	1.21508200
N	-0.03511700	1.40216800	1.21920900
C	6.41903500	-1.38701100	-1.46091600
C	6.98183500	-0.08715000	-1.55141300
C	5.07024900	-1.54760600	-1.23448300
C	-6.48495500	-0.05953200	-2.45101000
C	-7.12735600	-0.47266400	-1.25480400
C	-6.40306800	-0.68896200	-0.10172300
C	-5.12168400	0.13262700	-2.47919900
C	-0.69021600	3.07936500	-0.40075900
C	-0.33963500	2.70384200	1.03388300
C	6.19359700	1.03598100	-1.41609600
C	4.23482600	-0.40704900	-1.08989400
C	2.84401800	-0.52808200	-0.85174600
C	2.57707000	1.77531000	-0.81435000
C	3.91175900	1.99798000	-1.03635000
C	4.80039400	0.90152700	-1.18248200
C	-4.35094500	-0.08383000	-1.30516600
C	-2.94805600	0.10231700	-1.28487900
C	-2.82008100	-0.49339600	0.95079700
C	-4.17327200	-0.69678100	1.03873200

C	-4.99684400	-0.49939100	-0.09999400
C	0.19435700	-3.35239400	-0.08169500
C	0.45643900	-2.55823700	1.20350200
C	0.74281600	-3.29466100	2.36116700
C	1.00283700	-2.63653300	3.55443000
C	0.89821700	-1.24776600	3.58305000
C	0.56227800	-0.55851900	2.41839800
C	0.32452800	0.90119100	2.42211900
C	0.39503300	1.76199100	3.52491100
C	0.08353500	3.11162300	3.35841300
C	-0.29319600	3.59320800	2.10016300
H	8.04792700	0.01894900	-1.73032400
H	4.63011800	-2.53841200	-1.16368400
H	7.06067800	-2.25593900	-1.57176700
H	-7.07706900	0.10454200	-3.34627900
H	-8.20361600	-0.61888500	-1.25268100
H	-6.89645000	-1.00493600	0.81314700
H	-4.62196300	0.44871300	-3.39070200
H	6.62611200	2.03004900	-1.48705300
H	2.38040200	-1.51058400	-0.79172900
H	1.88256700	2.59800900	-0.71870800
H	4.28279900	3.01604300	-1.10409500
H	-2.42875200	0.42297900	-2.18047900
H	-2.17595200	-0.64259200	1.80732400
H	-4.60974000	-1.01011500	1.98201300
H	0.75806400	-4.37606000	2.29426500
H	1.25851700	-3.18757100	4.45380500
H	1.06062800	-0.70815800	4.50811900
H	0.68297100	1.39069900	4.50110300

H	0.13391300	3.78340500	4.20895700
H	-0.54385000	4.63407400	1.93150200
O	-0.16718900	-0.95831200	-2.24538700
H	0.23889300	-1.83620000	-1.95030100
H	0.45494400	-0.57475200	-2.88526700

$Ru^{III}(H_2O)(Bda)(Isoq)_2$

Ru	-0.04741300	-0.08758500	-0.26100300
O	-0.42428300	0.88226400	-2.12967900
O	0.08539200	-2.17518100	0.35150900
O	0.31387000	-3.54948300	2.12488400
O	-0.91070000	2.86363100	-3.08837000
N	2.09540300	-0.02345100	-0.63356500
N	-2.17536500	-0.25197300	-0.03942300
N	0.28743700	-0.04436200	1.78268800
N	-0.10377100	1.91388000	0.20479900
C	6.37430900	-2.27591600	-0.65862200
C	6.99493500	-1.11326700	-1.18592700
C	5.02281900	-2.28716300	-0.39721600
C	-6.29937500	-1.94339300	-1.90221900
C	-7.02822800	-1.38135200	-0.82175900
C	-6.38692100	-0.68381800	0.17958500
C	-4.93177100	-1.80083600	-1.96930500
C	-0.61096500	2.17297900	-2.12068100
C	-0.39557400	2.80088000	-0.75891200
C	6.26271400	0.02509100	-1.44760100
C	4.24510400	-1.12665500	-0.65670300
C	2.85473100	-1.09012700	-0.40283800
C	2.69372900	1.09873000	-1.14999000

C	4.03417400	1.16502500	-1.42888300
C	4.86826700	0.04382600	-1.18880000
C	-4.24563000	-1.08637000	-0.95055300
C	-2.84221700	-0.91407400	-0.97927200
C	-2.87504100	0.29202200	1.00728200
C	-4.23631200	0.18030100	1.12336300
C	-4.97845100	-0.51999200	0.13811400
C	0.26020200	-2.43446200	1.61965700
C	0.41624100	-1.19053800	2.46599100
C	0.66735000	-1.18349300	3.83432300
C	0.78686200	0.04481000	4.48501600
C	0.63757700	1.23102800	3.76371400
C	0.37925800	1.16488500	2.39385100
C	0.13819300	2.29605900	1.48450600
C	0.10021100	3.65137400	1.81693100
C	-0.19818100	4.58710900	0.82475400
C	-0.45476600	4.16174500	-0.47993900
H	8.06252000	-1.12585900	-1.38461600
H	4.53936600	-3.17206300	0.00654900
H	6.97445600	-3.15892900	-0.46213900
H	-6.82902200	-2.48804200	-2.67770900
H	-8.10660100	-1.50534200	-0.78775200
H	-6.94717700	-0.25499700	1.00529100
H	-4.36585700	-2.22754100	-2.79235900
H	6.73981400	0.91322700	-1.85146000
H	2.35548300	-1.96901200	-0.01285200
H	2.04988600	1.94637800	-1.33325000
H	4.45012100	2.08100900	-1.83594300
H	-2.25202600	-1.33285900	-1.78723700

H	-2.29649000	0.82020300	1.75274600
H	-4.73943300	0.63065300	1.97289200
H	0.76243100	-2.12898400	4.35518600
H	0.98768400	0.08426400	5.55006400
H	0.71595700	2.19094600	4.26001300
H	0.29455400	3.97060100	2.83390500
H	-0.23188200	5.64219100	1.07365400
H	-0.69366400	4.85211400	-1.28034200
O	-0.12044000	-1.80766200	-2.21213900
H	0.24006900	-2.54457500	-1.68768100
H	0.57987300	-1.54333600	-2.83117700

$Ru^{III}(OH)(Bda)(Isoq)_2$

Ru	-0.10930800	0.13576200	-0.45432500
O	-0.56345500	1.97632300	-1.39700600
O	1.17095500	-3.18314000	-0.88306900
O	-0.71720900	-4.02311300	0.05228200
O	-1.03655600	4.13892200	-0.97858700
N	2.01567500	0.41409200	-0.83664900
N	-2.22973600	-0.11244500	-0.19649500
N	0.39251700	-1.11072000	1.25682400
N	-0.01785000	1.48523100	1.10623000
C	6.48737800	-1.40047500	-1.14260000
C	7.00618100	-0.10823300	-1.41730700
C	5.13756400	-1.57929700	-0.93707500
C	-6.53330200	-0.48288000	-2.36358400
C	-7.17449800	-0.54808700	-1.09902800
C	-6.44472000	-0.49119300	0.06919500
C	-5.16433200	-0.36089400	-2.44484300

C	-0.68482700	3.01958300	-0.61584600
C	-0.31830100	2.76475500	0.83572100
C	6.17467800	0.98958000	-1.48559700
C	4.25878200	-0.46444500	-0.99921100
C	2.86569200	-0.60761800	-0.78763600
C	2.51372100	1.66173300	-1.12245200
C	3.84591600	1.90155700	-1.33906800
C	4.77964100	0.83535600	-1.27854400
C	-4.38803400	-0.29965600	-1.25637900
C	-2.97934400	-0.17343800	-1.29002200
C	-2.84473400	-0.17760100	1.02682400
C	-4.20311700	-0.30144900	1.16728400
C	-5.03234500	-0.36537200	0.01780900
C	0.29175900	-3.29324300	0.01776600
C	0.51466900	-2.45416500	1.28507700
C	0.83034800	-3.12750400	2.47579200
C	1.05912000	-2.41095500	3.63913100
C	0.92228800	-1.02217000	3.61104300
C	0.57272800	-0.39529400	2.41996300
C	0.33727000	1.06210700	2.33374700
C	0.41220000	1.99088500	3.37933600
C	0.10727500	3.32793900	3.12025800
C	-0.27043700	3.72920900	1.83511900
H	8.07387300	0.01226300	-1.57618200
H	4.72912200	-2.56356000	-0.72608500
H	7.16347800	-2.24871800	-1.09559100
H	-7.13114200	-0.53015900	-3.26861900
H	-8.25547200	-0.64440500	-1.05633000
H	-6.93791200	-0.54198300	1.03568000

H	-4.66464500	-0.31034100	-3.40808700
H	6.57400500	1.97731400	-1.69763000
H	2.44011500	-1.59322500	-0.60751200
H	1.78618800	2.45721400	-1.18915300
H	4.17927200	2.91041400	-1.56138800
H	-2.45359000	-0.13887700	-2.23723000
H	-2.19656000	-0.13021600	1.89167900
H	-4.63821100	-0.35074800	2.16034800
H	0.89152900	-4.20987900	2.45933300
H	1.32454400	-2.91571700	4.56238000
H	1.07491700	-0.43870600	4.51056400
H	0.69715000	1.68362100	4.37809900
H	0.16213800	4.05466900	3.92397400
H	-0.52016200	4.75696700	1.59939900
O	-0.29746300	-1.10067600	-1.96534900
H	0.46117800	-1.72150900	-1.97215800

$Ru^{III}(OOH)(Bda)(Isoq)_2$

Ru	0.00642400	-0.47360000	0.15295400
O	-0.11316800	-2.41029100	0.40522700
O	0.45981800	-0.45408300	2.20769400
O	-1.24164400	-1.00221300	-3.96230900
O	-0.42147100	-1.16861400	-1.87329100
O	1.33106400	0.52569900	4.02985500
N	-2.10062900	-0.46210100	0.61134500
N	2.10755600	-0.54197500	-0.27120800
N	-0.49018100	1.37705300	-1.48339800
N	0.37487700	1.79603800	0.87987900
C	-6.48168200	-2.38142800	-0.09209600

C	-7.04103300	-1.47013900	0.84109300
C	-5.13367300	-2.35224200	-0.37164300
C	6.51374700	-2.11721000	0.93940200
C	7.06201700	-1.49803700	-0.21400200
C	6.26000200	-0.79988000	-1.09149600
C	5.16481000	-2.03011100	1.20164500
C	0.90798800	0.56671900	2.87272100
C	0.83293300	1.88048100	2.13342100
C	-6.25088900	-0.54136200	1.48426700
C	-4.29632800	-1.40528400	0.27703300
C	-2.90542200	-1.33736700	0.02199500
C	-2.63704200	0.41835900	1.51472500
C	-3.97103700	0.43419800	1.83186000
C	-4.85825900	-0.48709200	1.21705300
C	4.31545700	-1.31678500	0.31366900
C	2.92321800	-1.20168600	0.54040200
C	2.63264600	0.05900500	-1.38558300
C	3.96729400	0.00543000	-1.69601800
C	4.86607200	-0.69248300	-0.84805600
C	-0.88401300	-0.50928800	-2.89084600
C	-0.92123800	0.98596000	-2.68096200
C	-1.31262500	1.89858400	-3.66192500
C	-1.22729900	3.25834800	-3.36084900
C	-0.74381500	3.66208900	-2.11475200
C	-0.37625900	2.67799200	-1.18889800
C	0.17282700	2.91882600	0.16448700
C	0.46854600	4.17922200	0.69543600
C	0.97714500	4.26508900	1.99124100
C	1.15777100	3.09812000	2.73138800

H	-8.10656200	-1.50889800	1.04810100
H	-4.69706200	-3.04593000	-1.08472200
H	-7.12524700	-3.10353000	-0.58533200
H	7.16657500	-2.66141700	1.61508300
H	8.12846300	-1.57823800	-0.40370100
H	6.68195600	-0.32698500	-1.97378300
H	4.73603900	-2.50101700	2.08187800
H	-6.68139100	0.15496200	2.19832800
H	-2.44138800	-2.01743300	-0.68290400
H	-1.94096300	1.11003300	1.96875800
H	-4.34309100	1.15347400	2.55472100
H	2.46911300	-1.66252200	1.41016000
H	1.92498600	0.58383700	-2.01267500
H	4.33133700	0.49904500	-2.59166000
H	-1.66007500	1.53910100	-4.62340300
H	-1.52306100	4.00306600	-4.09301500
H	-0.65749700	4.71618900	-1.87704700
H	0.30688900	5.07684800	0.10987800
H	1.21792400	5.23350000	2.41801300
H	1.52882600	3.10613000	3.74933300
O	0.69974100	-3.15851200	-0.53068500
H	0.35433500	-2.77898100	-1.37252100

$Ru^{IV}(O)(Bda)(Isoq)_2$

Ru	0.09947700	-0.13169100	-0.48652100
O	0.09610500	0.83590900	-2.00967700
O	0.64981500	-2.02683200	-1.26750000
O	-1.60246600	3.37816300	-0.94876700
O	0.58841700	3.74758700	-0.46963400

O	1.26464400	-4.11002700	-0.67937500
N	-2.01241900	-0.56008600	-0.74457700
N	2.21980200	0.23353100	-0.25511500
N	-0.44596800	1.21383000	1.15442900
N	0.14315900	-1.36360700	1.22184400
C	-6.48980000	1.12757000	-1.38934800
C	-6.99587100	-0.19838300	-1.41818900
C	-5.14250100	1.35417600	-1.21946600
C	6.58788800	0.03860100	-2.30887000
C	7.17664900	0.55056200	-1.12306700
C	6.40474800	0.85623400	-0.02254200
C	5.22802600	-0.16255500	-2.38069100
C	0.85942100	-2.98586100	-0.39959400
C	0.53198500	-2.63219600	1.04381800
C	-6.15587700	-1.28285800	-1.27756100
C	-4.25535900	0.25400700	-1.07177700
C	-2.86558800	0.44924800	-0.89104700
C	-2.49185000	-1.84441900	-0.78220400
C	-3.82156600	-2.13220700	-0.95161100
C	-4.76281300	-1.08097700	-1.10058700
C	4.40913700	0.14428300	-1.26076100
C	3.00861300	-0.04701900	-1.28586500
C	2.78216200	0.73031000	0.89191100
C	4.13007500	0.94790300	1.01689700
C	5.00035900	0.65998900	-0.06563900
C	-0.55209500	3.27893100	-0.26750300
C	-0.71229300	2.53166900	1.06362700
C	-1.13457300	3.26234600	2.18922100
C	-1.27261100	2.63248100	3.41306000

C	-0.97792800	1.26925300	3.50783800
C	-0.56636100	0.58050300	2.37440400
C	-0.20484800	-0.85584200	2.41476500
C	-0.17009500	-1.68878500	3.54074700
C	0.23202300	-3.01660500	3.38218500
C	0.59375500	-3.50669500	2.12268800
H	-8.06194200	-0.35615700	-1.55326400
H	-4.73965300	2.36261500	-1.19326400
H	-7.17401300	1.96297800	-1.50193200
H	7.21890300	-0.19396700	-3.16109900
H	8.25148400	0.70192700	-1.08728300
H	6.85808600	1.24796900	0.88334600
H	4.76822300	-0.55408300	-3.28352100
H	-6.54759500	-2.29568700	-1.30033500
H	-2.45896100	1.46028600	-0.88060300
H	-1.75768600	-2.63089200	-0.68354100
H	-4.14656900	-3.16752300	-0.97452500
H	2.52745700	-0.44109600	-2.17375100
H	2.10111800	0.95144000	1.70198200
H	4.52493400	1.34343700	1.94714300
H	-1.34676400	4.31991800	2.07515000
H	-1.59948400	3.18200800	4.28992600
H	-1.07022600	0.75546300	4.45655000
H	-0.44200800	-1.31951900	4.52190800
H	0.26467800	-3.66968500	4.24801200
H	0.91203700	-4.53109200	1.97051400

$Ru^{IV}(OH)(Bda)(Isoq)_2$

Ru	0.11564900	-0.15555300	-0.43727200

O	0.07786400	0.71968800	-2.13621000
O	0.63719600	-2.01699500	-1.17000900
O	-1.49972300	3.39575600	-1.02530300
O	0.73964100	3.46082800	-0.62749000
O	1.23648200	-4.10555700	-0.59204100
N	-1.98075300	-0.57765500	-0.71609200
N	2.20291300	0.25740600	-0.23259000
N	-0.44186300	1.21100700	1.15436700
N	0.11264200	-1.33175300	1.26619900
C	-6.42927200	1.15405900	-1.41265500
C	-6.94688500	-0.16742800	-1.43680400
C	-5.08217900	1.37049600	-1.23213700
C	6.61307800	-0.28116000	-2.11994100
C	7.15653200	0.53282800	-1.09213000
C	6.35150600	1.07568700	-0.11170200
C	5.26324800	-0.54580100	-2.15760600
C	0.83959800	-2.99273100	-0.29854200
C	0.49461600	-2.61240500	1.12307300
C	-6.11967300	-1.26068500	-1.28195200
C	-4.20892000	0.26094500	-1.06816200
C	-2.82282600	0.44353000	-0.87593200
C	-2.46793800	-1.86099100	-0.75693800
C	-3.79882000	-2.13124600	-0.93502700
C	-4.72812800	-1.07081500	-1.09406500
C	4.41131900	0.00123700	-1.15907800
C	3.02377800	-0.24771400	-1.15353800
C	2.71837700	1.05413000	0.76043900
C	4.05486400	1.34016900	0.84169900
C	4.95794800	0.82243000	-0.12271900

C	-0.44851800	3.18849900	-0.37195400
C	-0.68692500	2.52134800	0.99056200
C	-1.15231500	3.29820600	2.06788000
C	-1.33083600	2.71754500	3.31074800
C	-1.05122900	1.35564900	3.47485200
C	-0.61198800	0.61966000	2.38458700
C	-0.26512400	-0.80679400	2.45084100
C	-0.26325900	-1.62735900	3.58405600
C	0.13533900	-2.95779400	3.45759400
C	0.52594200	-3.46723600	2.21314600
H	-0.37304200	0.21342100	-2.84157500
H	-8.01295700	-0.31566600	-1.58057600
H	-4.67019300	2.37502900	-1.20936400
H	-7.10477600	1.99439100	-1.53763500
H	7.27089200	-0.69434400	-2.87790000
H	8.22443600	0.72918600	-1.08127900
H	6.77291200	1.69796900	0.67189000
H	4.83674000	-1.16746300	-2.93909900
H	-6.52150700	-2.26917600	-1.30278000
H	-2.40932900	1.45059500	-0.87855400
H	-1.74586400	-2.65655100	-0.65234400
H	-4.13322300	-3.16317300	-0.95816600
H	2.57739700	-0.87643800	-1.91484100
H	2.01294800	1.45141900	1.47410500
H	4.41549000	1.97111500	1.64722100
H	-1.35675100	4.34974200	1.90107900
H	-1.68145500	3.30447200	4.15292300
H	-1.17997500	0.88155000	4.43971900
H	-0.56144700	-1.23755500	4.54925700

H	0.14047700	-3.59925600	4.33198000
H	0.84055000	-4.49528100	2.07957900

$Ru^V(O)(Bda)(Isoq)_2$

Ru	0.08256300	-0.07843300	-0.50149300
O	0.03886900	1.01590900	-1.83904600
O	0.58339200	-1.87728200	-1.26050900
O	-1.30789200	3.33081900	-0.89402500
O	0.82235900	3.65479500	-0.15420200
O	1.26954500	-3.98751600	-0.94293300
N	-2.02698800	-0.48452400	-0.77362800
N	2.19602500	0.26572400	-0.25607500
N	-0.43088900	1.06743900	1.24486200
N	0.11919000	-1.44675800	1.17852800
C	-6.47971200	1.26442000	-1.38217600
C	-6.99567300	-0.05610300	-1.45276900
C	-5.13214700	1.47701200	-1.20018100
C	6.56952700	-0.00529100	-2.27379700
C	7.14880200	0.55174600	-1.10355800
C	6.36993300	0.89975100	-0.02045100
C	5.21090600	-0.20987500	-2.34976400
C	0.84852200	-2.94211400	-0.49648200
C	0.51966500	-2.70251500	0.94763200
C	-6.16553000	-1.15159700	-1.34200200
C	-4.25508300	0.36513700	-1.08146000
C	-2.86669500	0.54239400	-0.89034800
C	-2.51141300	-1.76559500	-0.85346100
C	-3.84312400	-2.03015900	-1.03594500
C	-4.77280900	-0.96544400	-1.15379400

C	4.38538500	0.13993800	-1.24713400
C	2.98800100	-0.05446400	-1.27585700
C	2.74345900	0.81115200	0.87739000
C	4.09029600	1.03162700	0.99773100
C	4.96696300	0.70185200	-0.06704900
C	-0.33749100	3.20584600	-0.11277500
C	-0.63068400	2.39360900	1.17023700
C	-1.07800800	3.09947800	2.30262200
C	-1.30012300	2.43247600	3.49328500
C	-1.05019300	1.05749100	3.56202800
C	-0.61069900	0.39107900	2.43047900
C	-0.25495200	-1.03180000	2.39822400
C	-0.23743400	-1.93086600	3.47132800
C	0.18359800	-3.24099300	3.24439500
C	0.57619200	-3.64533400	1.96327100
H	-8.06217500	-0.20088200	-1.59693300
H	-4.72475100	2.48199500	-1.14339000
H	-7.15701400	2.10777800	-1.47271100
H	7.20750700	-0.26996400	-3.11105300
H	8.22322200	0.70426400	-1.06579800
H	6.81677800	1.32568300	0.87275000
H	4.75816700	-0.63587000	-3.24008800
H	-6.56460300	-2.15991300	-1.39743600
H	-2.44706700	1.54702300	-0.85073500
H	-1.78907600	-2.56404700	-0.77719000
H	-4.17714200	-3.06087700	-1.09307800
H	2.51810800	-0.48670300	-2.15224100
H	2.05938400	1.06405400	1.67379600
H	4.47833400	1.46298200	1.91446900

H	-1.23598000	4.16841600	2.21647800
H	-1.65006300	2.96579800	4.37061400
H	-1.19175700	0.51671500	4.48931500
H	-0.53790000	-1.61866600	4.46379000
H	0.20617400	-3.94662000	4.06759600
H	0.90866900	-4.65423000	1.74970400

$Ru^{II}(H_2O)(Bda)(pic)_2$

Ru	0.02311300	-0.20688000	-0.59434000
O	0.12670900	-2.22674800	-1.34984600
O	-0.40805400	3.09764800	-1.58033800
O	1.28172900	4.19919300	-0.56556000
O	0.28948400	-4.33891400	-0.57279500
N	-2.12981100	-0.18436100	-0.79333500
N	2.18046600	-0.22052500	-0.58005000
N	-0.11537000	1.40239800	0.88149000
N	-0.10350100	-1.23692800	1.08925200
C	0.15702700	-3.12258800	-0.40734700
C	-0.01773200	-2.58129800	1.00528400
C	-4.15555100	1.04648000	-1.19867100
C	-2.77617800	0.97621500	-1.04328800
C	-2.87754100	-1.30323300	-0.69950200
C	-4.25941400	-1.30497300	-0.83939600
C	-4.94092000	-0.10792100	-1.09218200
C	4.21402100	-0.93444700	-1.65472700
C	2.83082400	-0.91654100	-1.54110000
C	2.93613400	0.46341000	0.30345800
C	4.32510300	0.48387200	0.25537600
C	5.00553000	-0.22282400	-0.74271900

C	0.33652800	3.39367700	-0.58822500
C	0.00554800	2.74401500	0.76096200
C	-0.12117300	3.60170600	1.86243100
C	-0.41618800	3.08330900	3.11476500
C	-0.50352300	1.70034100	3.25593300
C	-0.32379000	0.88123800	2.14204600
C	-0.29861400	-0.59193700	2.26105900
C	-0.41673000	-1.34013400	3.43945400
C	-0.32518200	-2.73071800	3.37822800
C	-0.12060400	-3.36447600	2.14813600
H	-2.14964800	1.85908500	-1.13306700
H	-2.34364100	-2.22646100	-0.51646200
H	-4.79723600	-2.24430100	-0.75407400
H	2.20329400	-1.48524800	-2.21710000
H	2.40298000	1.01400700	1.06806200
H	4.86963300	1.05758700	0.99882400
H	0.01279200	4.66548400	1.70613400
H	-0.55140600	3.73383400	3.97289000
H	-0.69639300	1.26316300	4.22799200
H	-0.57290200	-0.85124600	4.39347300
H	-0.41323000	-3.31573500	4.28779600
H	-0.04682300	-4.44204000	2.05819900
H	-4.61305800	2.00976600	-1.40367800
C	-6.44072800	-0.06313600	-1.22134100
H	-6.90548000	0.06105500	-0.23456700
H	-6.76258600	0.77687200	-1.84407700
H	-6.83160100	-0.99026400	-1.65176600
C	6.50887700	-0.23633200	-0.82774000
H	6.90164400	-1.21856100	-0.53662500

H	6.84645000	-0.04538300	-1.85221100
H	6.95412000	0.51375200	-0.16854300
H	4.66957900	-1.50897500	-2.45560000
O	0.09781400	0.80409500	-2.57404400
H	0.99650000	0.77908000	-2.94262200
H	-0.06824800	1.76876700	-2.31819300

$Ru^{III}(H_2O)(Bda)(pic)_2$

Ru	-0.00186600	-0.07491500	-0.51235100
O	-0.07185600	-2.13262700	-1.07242200
O	-0.04025000	1.77046600	-1.63689100
O	-0.21674700	4.01296100	-1.47597200
O	-0.15281100	-4.24001300	-0.27760200
N	-2.14296500	-0.10660900	-0.60728000
N	2.16757800	-0.10005400	-0.61664000
N	-0.01308200	1.42790800	0.90924600
N	0.02776800	-1.08862800	1.27639800
C	-0.08697100	-3.02676000	-0.11897600
C	-0.00324900	-2.42846100	1.26774200
C	-4.34419500	0.07927000	0.33063300
C	-2.96139900	0.11726000	0.44031900
C	-2.70426900	-0.37918100	-1.80452000
C	-4.07997700	-0.43084900	-1.98370200
C	-4.94283900	-0.20252000	-0.90421800
C	4.17812700	-1.23554200	-1.28415800
C	2.79993700	-1.17843800	-1.12857900
C	2.92805600	0.95270400	-0.25119600
C	4.31007500	0.96267300	-0.37646800
C	4.97731200	-0.15099300	-0.90205600

C	-0.12892100	2.89615000	-0.98118200
C	-0.09377400	2.71012600	0.52092100
C	-0.11424100	3.73893200	1.45640600
C	-0.04325700	3.41012400	2.81132200
C	0.03763100	2.07177500	3.20055600
C	0.04637200	1.07824900	2.21995600
C	0.08028900	-0.37770900	2.43146200
C	0.12268700	-1.05140900	3.65309700
C	0.10355500	-2.44766400	3.66029500
C	0.03341800	-3.14972900	2.45615600
H	-2.49431400	0.33525500	1.39231600
H	-2.01702100	-0.55077600	-2.62452500
H	-4.47284800	-0.64944500	-2.97151300
H	2.16751700	-2.00636400	-1.42310800
H	2.41288900	1.81417900	0.15188900
H	4.85893500	1.84536700	-0.06451000
H	-0.17996900	4.76355400	1.10978700
H	-0.05199900	4.18961100	3.56538000
H	0.08976500	1.80622500	4.24963500
H	0.16470600	-0.49697900	4.58297400
H	0.13655400	-2.98058400	4.60431600
H	0.00488800	-4.23223100	2.41501700
H	-4.94731100	0.27245400	1.21187800
C	-6.43820400	-0.27806500	-1.05549000
H	-6.79263700	-1.28649300	-0.80596900
H	-6.94164900	0.42163900	-0.38167300
H	-6.74664800	-0.06429300	-2.08273900
C	6.47597800	-0.18741000	-1.02939100
H	6.92558800	-0.54406600	-0.09373500

H	6.79155500	-0.86602900	-1.82687300
H	6.88274700	0.80828100	-1.22943700
H	4.62155100	-2.13061100	-1.70851600
O	0.13230000	-0.28629100	-3.20779000
H	0.97197600	-0.70566600	-3.45630800
H	0.30478200	0.67377300	-3.21105800

$Ru^{III}(OH)(Bda)(pic)_2$

Ru	0.04503200	-0.13617900	-0.63774200
O	0.26976700	-2.08309200	-1.42701900
O	-0.98206300	4.02153400	-0.86771400
O	1.10749200	3.14321600	-1.01096600
O	0.55810100	-4.24376600	-0.85812700
N	-2.10746800	-0.23161500	-0.81405000
N	2.19201900	-0.01159700	-0.62939600
N	-0.16126700	1.28951600	0.98584900
N	0.02400300	-1.32821400	1.05043700
C	0.36795500	-3.06582800	-0.56753300
C	0.19981300	-2.64805400	0.88174100
C	-4.18170400	0.91312700	-1.20184100
C	-2.80130600	0.90342500	-1.04404000
C	-2.79856800	-1.38648200	-0.74175700
C	-4.17740000	-1.44928800	-0.89081900
C	-4.91141900	-0.27933800	-1.12473000
C	4.24812200	-0.50069500	-1.77225400
C	2.86494100	-0.56345700	-1.66028400
C	2.90919300	0.62157400	0.32046000
C	4.29326700	0.71471300	0.27745000
C	5.00443300	0.14613300	-0.78757000

C	-0.01852400	3.32555900	-0.47853800
C	-0.24250900	2.63216300	0.87608100
C	-0.52437700	3.42993500	1.99522700
C	-0.67061600	2.84785500	3.24489700
C	-0.55589800	1.46186400	3.35998600
C	-0.31278600	0.70163200	2.22046700
C	-0.16704900	-0.76838700	2.25962400
C	-0.18720800	-1.58680300	3.39597000
C	-0.00375100	-2.96179700	3.24402000
C	0.19460600	-3.50920300	1.97269700
H	-2.22793100	1.82110500	-1.10769500
H	-2.22309900	-2.28651600	-0.56932800
H	-4.67123200	-2.41381300	-0.82689100
H	2.26363900	-1.07344600	-2.40230500
H	2.34980200	1.06952000	1.13048300
H	4.81090900	1.23793800	1.07531000
H	-0.61981800	4.50047000	1.85468800
H	-0.87311400	3.45392400	4.12207200
H	-0.66152400	0.98109400	4.32470000
H	-0.33830100	-1.16523700	4.38223100
H	-0.01486000	-3.60354300	4.11868000
H	0.33957300	-4.57160100	1.81588200
H	-4.68281700	1.85832000	-1.38626000
C	-6.41041200	-0.30219600	-1.26281500
H	-6.88383900	-0.25739000	-0.27350400
H	-6.77088000	0.55382100	-1.84044100
H	-6.75134100	-1.22288300	-1.74615500
C	6.50711500	0.20907800	-0.85502200
H	6.94842100	-0.60162800	-0.26100800

H	6.86574800	0.09929500	-1.88242700
H	6.88391900	1.15281400	-0.44869000
H	4.72884700	-0.96016400	-2.63016200
O	-0.01865200	0.96635700	-2.25490000
H	0.68282700	1.64599100	-2.15711200

$Ru^{III}(OOH)(Bda)(pic)_2$

Ru	-0.03995200	0.08566000	-0.61036400
O	-0.25959000	-0.82885500	-2.32961800
O	-0.17459700	2.02757000	-1.44991500
O	0.93123400	-3.32375000	-1.02639900
O	-1.24435800	-3.83530700	-0.62094200
O	-0.38197000	4.20805500	-0.92722000
N	2.13674100	0.15878800	-0.71383000
N	-2.19826800	0.09529200	-0.59104100
N	0.06820800	-1.30167400	1.06991200
N	0.03405900	1.32150200	1.05258400
C	-0.22887100	3.03097900	-0.61230500
C	-0.05924800	2.64292400	0.84445300
C	4.28473800	-0.89295000	-0.48376300
C	2.89577900	-0.93077000	-0.46864500
C	2.77635200	1.31009100	-1.00606000
C	4.16037200	1.41719300	-1.04256400
C	4.95900100	0.30096700	-0.76569800
C	-4.22387900	0.46817400	-1.82580100
C	-2.83959200	0.40258600	-1.73718600
C	-2.94922400	-0.15345000	0.50050500
C	-4.33648700	-0.10515700	0.48517700
C	-5.01467200	0.21586000	-0.69793100

C	-0.14520700	-3.32800300	-0.34910900
C	-0.02827300	-2.64519100	1.01366300
C	-0.01414500	-3.42763800	2.17649700
C	0.14391200	-2.81902100	3.41242300
C	0.24078100	-1.42804700	3.47213700
C	0.18193300	-0.68824600	2.29496300
C	0.17747600	0.78920700	2.28063700
C	0.26006800	1.63915900	3.39040400
C	0.17536100	3.01810500	3.19486000
C	0.00397100	3.53583700	1.90799500
H	2.36316200	-1.86024000	-0.30241000
H	2.15048100	2.16102400	-1.23799800
H	4.60799600	2.37441700	-1.29120100
H	-2.21079700	0.58782800	-2.59855600
H	-2.41895200	-0.40420400	1.41040600
H	-4.88136500	-0.32100300	1.39874500
H	-0.12009500	-4.50297200	2.08603700
H	0.17816600	-3.40830300	4.32292700
H	0.34307300	-0.92826400	4.42738600
H	0.38359200	1.23941100	4.38934600
H	0.23759200	3.68516400	4.04816300
H	-0.07720100	4.59941300	1.71740800
H	4.83600600	-1.80599300	-0.28034600
C	6.46205300	0.38527300	-0.75959900
H	6.82109500	0.69448300	0.23059600
H	6.91705300	-0.58322700	-0.98688800
H	6.82254000	1.12307700	-1.48284900
C	-6.51652700	0.30649000	-0.74629000
H	-6.84708700	1.30188600	-0.42261600

H	-6.89372600	0.14657100	-1.76036600
H	-6.98055800	-0.42486500	-0.07749800
H	-4.67953600	0.71662000	-2.77910800
O	0.85006800	-1.50711500	-2.92927300
H	0.96093500	-2.26823200	-2.28418100

$Ru^{IV}(O)(Bda)(pic)_2$

Ru	0.04064900	-0.10368800	-0.71501500
O	0.03915600	0.78375500	-2.28641800
O	0.29818900	-2.08867800	-1.42476000
O	-1.20602500	3.57221400	-1.25612000
O	1.04196800	3.67568700	-0.93326600
O	0.69213100	-4.20329200	-0.76342800
N	-2.11956800	-0.28348200	-0.80574800
N	2.19772600	0.00554300	-0.64903900
N	-0.20488600	1.37425300	0.88267200
N	0.05784700	-1.25095800	1.05195300
C	0.44922900	-3.02475800	-0.52099800
C	0.27005500	-2.56604700	0.91903200
C	-4.26180300	0.76508800	-1.03949100
C	-2.87613000	0.82599200	-0.95528800
C	-2.74365100	-1.47689000	-0.74848700
C	-4.12343900	-1.60446300	-0.82338000
C	-4.92604200	-0.46550200	-0.96786800
C	4.26463400	-0.34045900	-1.81570500
C	2.87970600	-0.36523500	-1.75373300
C	2.90203400	0.41073700	0.42398600
C	4.29059200	0.45707600	0.43231400
C	5.01224900	0.07519500	-0.70418700

C	-0.13317800	3.36850700	-0.63771300
C	-0.29594500	2.71075500	0.73898400
C	-0.53247900	3.54182600	1.84935800
C	-0.66561400	2.99209800	3.11176700
C	-0.54888400	1.60693500	3.26037100
C	-0.31667800	0.81900700	2.14075500
C	-0.14100800	-0.64887600	2.23513100
C	-0.13627100	-1.42700300	3.40006800
C	0.08286700	-2.80158600	3.28895400
C	0.29395700	-3.39107100	2.03772600
H	-2.35090700	1.77669600	-1.02255300
H	-2.11435500	-2.35136400	-0.65433800
H	-4.56431900	-2.59494800	-0.77379300
H	2.28253800	-0.68332000	-2.59881500
H	2.33513900	0.71136700	1.29567500
H	4.80073100	0.79496500	1.32841100
H	-0.60676800	4.61253800	1.69262600
H	-0.85154800	3.61973600	3.97734700
H	-0.63652400	1.15220900	4.23927600
H	-0.29359400	-0.98037700	4.37408800
H	0.09066200	-3.41359200	4.18487500
H	0.46890900	-4.45398100	1.92145200
H	-4.81918500	1.68829600	-1.16452100
C	-6.42708100	-0.56020200	-1.02432800
H	-6.84580700	-0.54028800	-0.00985600
H	-6.85903200	0.27998700	-1.57555700
H	-6.74993300	-1.49461600	-1.49315700
C	6.51679000	0.09526800	-0.73502900
H	6.91191700	-0.92813600	-0.73680600

H	6.88385100	0.58414500	-1.64377900
H	6.92868200	0.61572200	0.13350200
H	4.75634200	-0.64608900	-2.73379900

$Ru^{IV}(OH)(Bda)(pic)_2$

Ru	0.00031700	0.01209300	-0.66654000
O	-0.06094300	0.06729900	-2.60764500
O	0.15652800	-2.00512700	-1.30626100
O	-0.85330600	4.12658400	-0.68638900
O	-0.14141900	2.05986800	-1.19861900
O	0.77958700	-4.12137900	-0.88853300
N	-2.13353000	-0.20967700	-0.76103400
N	2.13863800	0.24855100	-0.75661500
N	-0.31303100	1.18173300	1.14854300
N	0.31410600	-1.25897400	1.08815800
C	0.54314600	-2.97741900	-0.52456600
C	0.62722200	-2.54949600	0.90464400
C	-4.19538200	0.38755400	-1.82442100
C	-2.81896800	0.49932600	-1.68078500
C	-2.81763900	-1.05563400	0.03493100
C	-4.19234000	-1.21592900	-0.05795100
C	-4.92208500	-0.48664100	-1.00655400
C	4.25100500	-0.45000000	-1.65347400
C	2.87511200	-0.58017400	-1.52793100
C	2.77649400	1.23607600	-0.09553600
C	4.14914500	1.41934100	-0.17509300
C	4.92811700	0.56865900	-0.97075500
C	-0.58041100	2.97587300	-0.37606000
C	-0.65224700	2.47435800	1.02960300

C	-0.94718800	3.27100300	2.12884300
C	-0.86330000	2.70758400	3.40055100
C	-0.46878200	1.37766200	3.52756700
C	-0.19681900	0.63052600	2.37925300
C	0.22379000	-0.76402300	2.34453300
C	0.50129700	-1.56669800	3.45313900
C	0.86972200	-2.89627200	3.25863000
C	0.92182900	-3.40218500	1.96144600
H	-2.23111200	1.15632400	-2.30801600
H	-2.24466000	-1.61528500	0.76300700
H	-4.68707400	-1.91029500	0.61326300
H	2.33238800	-1.36013700	-2.04751700
H	2.16684100	1.88994300	0.51364600
H	4.60425800	2.22857600	0.38647400
H	-1.21666100	4.30737300	1.96230900
H	-1.08530700	3.29615300	4.28383600
H	-0.37384600	0.92301900	4.50595800
H	0.42890500	-1.15610500	4.45263700
H	1.09513100	-3.52853000	4.11030600
H	1.16851600	-4.43460800	1.74329600
H	-4.69478300	0.98735000	-2.57840900
C	-6.41000600	-0.65474900	-1.15410100
H	-6.87287700	0.24216200	-1.57515500
H	-6.62939000	-1.49020900	-1.83132600
H	-6.88203700	-0.87976600	-0.19315000
C	6.41470300	0.75569900	-1.10476600
H	6.92029400	-0.19593100	-1.29228700
H	6.63516600	1.41878700	-1.95123200
H	6.84119000	1.21375700	-0.20787100

H	4.78837200	-1.14905600	-2.28591900
H	0.73741200	0.54836600	-2.89675200

$Ru^{V}(O)(Bda)(pic)_2$

Ru	0.03573500	-0.05605400	-0.72627400
O	0.05413300	0.95857100	-2.12594700
O	0.22491300	-1.94722900	-1.40126300
O	-0.80912600	3.48080700	-1.26164600
O	1.37608800	3.54455500	-0.62077500
O	0.60640300	-4.11799900	-0.99219100
N	-2.12118600	-0.16318500	-0.85012100
N	2.18586200	-0.00919600	-0.63369900
N	-0.18110100	1.24311000	0.97398100
N	-0.01795300	-1.32504900	1.02952200
C	0.37470900	-2.99840300	-0.58888600
C	0.17580000	-2.63729400	0.85391900
C	-4.21197000	0.97895700	-1.06363900
C	-2.82803500	0.98414400	-0.96196400
C	-2.78508400	-1.33739100	-0.85484200
C	-4.16604200	-1.40405000	-0.95225000
C	-4.92212200	-0.22838300	-1.05347000
C	4.24593000	-0.44367400	-1.77306000
C	2.86114600	-0.45681700	-1.71412500
C	2.88696300	0.46489900	0.41667600
C	4.27280700	0.49866600	0.42085000
C	4.99324100	0.03722300	-0.68911300
C	0.16798300	3.26652100	-0.50909700
C	-0.17484400	2.57862900	0.83234100
C	-0.43946600	3.40193800	1.94272100

C	-0.69439300	2.83875200	3.17966100
C	-0.65408900	1.44673200	3.31693700
C	-0.38850100	0.66429100	2.20579800
C	-0.25191300	-0.79584000	2.23999900
C	-0.30352600	-1.63023900	3.36278000
C	-0.09752200	-2.99926000	3.19347400
C	0.15194900	-3.52410300	1.92020500
H	-2.26454900	1.91385900	-0.99341700
H	-2.19380600	-2.23970000	-0.79281100
H	-4.64407100	-2.37794400	-0.95327900
H	2.26910700	-0.83064300	-2.54008200
H	2.32313700	0.82730800	1.26487600
H	4.78347800	0.89137500	1.29370500
H	-0.43242200	4.47679900	1.80254800
H	-0.90698400	3.46374100	4.04035000
H	-0.82046900	0.98087700	4.28018300
H	-0.49260900	-1.22363800	4.34843200
H	-0.13054600	-3.65658200	4.05530800
H	0.31618900	-4.58155600	1.75065000
H	-4.73054400	1.92757900	-1.15648100
C	-6.42318700	-0.26339400	-1.13149800
H	-6.84967400	-0.30258500	-0.12088400
H	-6.81777100	0.62984100	-1.62332500
H	-6.77230800	-1.14998800	-1.66883800
C	6.49651900	0.03801300	-0.70615300
H	6.87584300	-0.87961000	-0.23830200
H	6.88402500	0.07360800	-1.72791200
H	6.89947300	0.88416000	-0.14209400
H	4.73541600	-0.80944100	-2.66935600

$Ru^{II}(H_2O)(EtOtpy)(pic)_2$

O	-1.05265600	4.74000900	-1.04815000
N	-0.16638500	0.83535300	-0.18901100
N	-0.14701800	-0.35978800	2.14571500
N	0.49260600	-0.99669300	-1.95500400
C	-0.54402200	1.63182300	0.83491400
C	-0.86119200	2.97198700	0.61142700
C	-0.77746700	3.47554100	-0.69656200
C	-0.37935600	2.62241800	-1.74370100
C	-0.07798400	1.29819900	-1.46324600
C	-0.54234500	0.95320800	2.15303300
C	-0.88277800	1.59551600	3.34430100
C	-0.81021400	0.89897200	4.54947000
C	-0.38825700	-0.42803400	4.53708000
C	-0.06780500	-1.01510500	3.31519000
C	0.32551900	0.26954100	-2.45175400
C	0.50994600	0.53729800	-3.80848800
C	0.86484500	-0.49784100	-4.67259600
C	1.02401600	-1.78324900	-4.15991000
C	0.82932200	-1.98772000	-2.79585000
C	-1.47111500	5.68806900	-0.03956800
C	-1.70530800	7.01412800	-0.73687200
H	-1.16147000	3.60680500	1.43256100
H	-0.32347500	3.02513800	-2.74716600
H	-1.19937600	2.63141400	3.33655200
H	-1.07398500	1.39086300	5.47969700
H	-0.30618100	-1.00791600	5.44930900
H	0.26626100	-2.04519200	3.26286900
H	0.37998400	1.54297200	-4.18971200

H	1.01133700	-0.29852300	-5.72885900
H	1.29498500	-2.61891700	-4.79514300
H	0.94169400	-2.96937500	-2.34884900
H	-2.38712700	5.32044900	0.43720700
H	-0.68566900	5.76979200	0.72074100
H	-2.48656500	6.92097200	-1.49751700
H	-2.02414200	7.75992800	-0.00186900
H	-0.78750400	7.36934400	-1.21549400
C	3.24717100	-1.58867800	-0.13534200
C	2.85710900	0.39795300	0.96643400
C	4.62112500	-1.41645100	-0.03936900
H	2.83083900	-2.46880300	-0.60986500
C	4.21873300	0.63735400	1.09739500
H	2.13831000	1.10397200	1.36225600
H	5.27715100	-2.17530700	-0.45408700
H	4.54815900	1.54030100	1.60167000
C	-2.41964400	-2.41091400	0.93017600
C	-2.62881000	-1.24050600	-1.04270200
C	-3.74149900	-2.82861200	0.86999400
H	-1.78919700	-2.70920000	1.75940600
C	-3.96075600	-1.61443700	-1.16663300
H	-2.16615100	-0.61497000	-1.79508300
H	-4.12914200	-3.46376600	1.66022200
H	-4.52602900	-1.26767200	-2.02577500
Ru	0.24017500	-1.07957300	0.15810300
N	-1.84868800	-1.62668600	-0.01154300
N	2.35867600	-0.69912500	0.35935500
C	5.14643200	-0.27828000	0.58594400
C	-4.55699300	-2.42972900	-0.19751100

C	6.62982600	-0.04178400	0.68205100
H	7.18010000	-0.98551300	0.74176900
H	6.98762100	0.49141600	-0.20824100
H	6.88054500	0.56915500	1.55412000
C	-6.00292800	-2.83871400	-0.28242600
H	-6.14717200	-3.86221900	0.07734400
H	-6.62051500	-2.18262600	0.34431600
H	-6.37839700	-2.76894500	-1.30711300
O	0.71256800	-3.27617900	0.37331700
H	-0.05044900	-3.85933600	0.21802400
H	1.10078600	-3.57678600	1.21296100

$Ru^{III}(H_2O)(EtOtpy)(pic)_2$

O	-2.64583000	4.03986000	-1.04383400
N	-0.42465000	0.72316500	-0.18410300
N	0.18231400	-0.26637300	2.12819900
N	0.80707300	-0.76424100	-1.92721400
C	-0.99959100	1.37843400	0.84821200
C	-1.76924600	2.51518300	0.62716600
C	-1.93339600	2.97488000	-0.69288800
C	-1.29598200	2.28632100	-1.74664100
C	-0.54640700	1.15901900	-1.46465200
C	-0.66853400	0.80538300	2.16609500
C	-1.10949500	1.31821000	3.38256400
C	-0.66550000	0.73812000	4.57102100
C	0.22159200	-0.33432900	4.51873900
C	0.62942100	-0.80819000	3.27528500
C	0.17542400	0.33098800	-2.44862000
C	0.23940500	0.59859900	-3.81308000

C	0.95300600	-0.26392400	-4.64596700
C	1.58699700	-1.37749400	-4.09916000
C	1.49795800	-1.59386600	-2.72765800
C	-3.34718400	4.81514600	-0.03394700
C	-4.06925600	5.93653200	-0.75287600
H	-2.21898100	3.03628200	1.45979000
H	-1.40897000	2.65908800	-2.75656100
H	-1.78669000	2.16287200	3.40742800
H	-1.00368100	1.13024500	5.52390300
H	0.59999200	-0.80536900	5.41806400
H	1.31645000	-1.64072300	3.18844900
H	-0.25830200	1.46827000	-4.22356500
H	1.00954800	-0.06357700	-5.71024800
H	2.14475400	-2.07419300	-4.71349200
H	1.96311600	-2.44927700	-2.25301300
H	-4.04647100	4.15433700	0.48910500
H	-2.61393500	5.20188200	0.68165100
H	-4.79042500	5.53733200	-1.47233700
H	-4.60978500	6.54361200	-0.01997600
H	-3.35994700	6.58062000	-1.28121500
C	3.62454100	-0.71638200	-0.10611900
C	2.66122300	1.20363200	0.75116200
C	4.88837900	-0.15063700	-0.05010800
H	3.49255100	-1.72427300	-0.47800000
C	3.89610900	1.82959300	0.83118800
H	1.76618800	1.72414100	1.06283500
H	5.73873700	-0.73776200	-0.38045800
H	3.94366900	2.84376800	1.21334800
C	-1.81186300	-2.68383900	1.13981800

C	-2.06965600	-2.06205700	-1.07526500
C	-3.01237600	-3.37606800	1.12156300
H	-1.21747100	-2.65079700	2.04446000
C	-3.27801100	-2.73672200	-1.15962800
H	-1.67443400	-1.54373500	-1.93794500
H	-3.33624900	-3.88281800	2.02447700
H	-3.81576300	-2.72859500	-2.10176400
Ru	0.57210400	-1.00407400	0.16447900
N	-1.33297600	-2.02683400	0.05705700
N	2.51364800	-0.05766300	0.29209200
C	5.05556900	1.15748400	0.42478400
C	-3.78672100	-3.41667400	-0.04583000
C	6.40782600	1.81246100	0.46987700
H	7.19341100	1.08523300	0.69483400
H	6.64094400	2.25614300	-0.50662800
H	6.43900600	2.61274000	1.21405100
C	-5.10642200	-4.13454200	-0.09160700
H	-5.10319200	-5.01322100	0.55977600
H	-5.90577700	-3.46866900	0.25830800
H	-5.35597200	-4.44575300	-1.10951000
O	1.57636700	-2.94263800	0.25546400
H	0.98691300	-3.71919200	0.21741500
H	2.22918100	-3.13506400	0.95391500

$Ru^{III}(OH)(EtOtpy)(pic)_2$

O	2.18142200	4.35193500	1.09415200
N	0.34339100	0.80541200	0.21967200
N	-0.37728300	-0.12361500	-2.10207100
N	-0.26580500	-1.05714500	1.93668600

C	0.56609500	1.66309100	-0.79575400
C	1.18437900	2.89140800	-0.57161500
C	1.57580700	3.21588800	0.73908800
C	1.32861000	2.30098700	1.78291100
C	0.70684800	1.09784900	1.49159100
C	0.10604400	1.15563800	-2.10851000
C	0.14983700	1.89044600	-3.29207700
C	-0.30567300	1.31235700	-4.47660900
C	-0.79579400	0.00893000	-4.45361000
C	-0.81771800	-0.67845800	-3.24288800
C	0.33931500	0.04730400	2.46686200
C	0.54154700	0.15324100	3.84216100
C	0.11267700	-0.87439400	4.68091500
C	-0.52066700	-1.98513400	4.12782900
C	-0.69350300	-2.03934300	2.74778100
C	2.47805500	5.35513100	0.09125300
C	3.15984300	6.50980300	0.79817500
H	1.36276500	3.57207400	-1.39151900
H	1.63150000	2.56516900	2.78812300
H	0.52967300	2.90472800	-3.29182500
H	-0.27658800	1.87739500	-5.40197800
H	-1.15839000	-0.47821200	-5.35127400
H	-1.18765900	-1.69389600	-3.16600400
H	1.02280400	1.03081200	4.25622500
H	0.26636000	-0.80062600	5.75207100
H	-0.87922200	-2.80141600	4.74398200
H	-1.18179700	-2.87794700	2.26490400
H	3.12829500	4.91127500	-0.67078700
H	1.54082000	5.67041200	-0.38053800

H	4.08972500	6.18141400	1.27239800
H	3.39922100	7.28979300	0.06859400
H	2.50535800	6.93922900	1.56279900
C	-3.50714600	-1.10393400	-0.54567900
C	-2.83998400	0.78877500	0.59863900
C	-4.83791700	-0.71308300	-0.48364400
H	-3.20868300	-2.03261300	-1.01570500
C	-4.14943900	1.23773900	0.69842500
H	-2.02905200	1.37138600	1.01583400
H	-5.59308000	-1.34879100	-0.93503600
H	-4.34547600	2.17862400	1.20248000
C	1.85004100	-2.99103700	0.36327500
C	2.45581100	-1.41750000	-1.21176000
C	3.09084600	-3.60782800	0.28067500
H	1.09504600	-3.36995400	1.04135200
C	3.71633000	-1.98116800	-1.34386900
H	2.18251500	-0.54916600	-1.79593600
H	3.28888500	-4.47478700	0.90240200
H	4.42083400	-1.53355700	-2.03742000
Ru	-0.46313200	-1.06108400	-0.18708200
N	1.51948600	-1.90621900	-0.37056300
N	-2.50894600	-0.36702600	-0.01347600
C	-5.19479500	0.48329900	0.15092500
C	4.06664800	-3.10997600	-0.59110400
C	-6.62948200	0.92413400	0.26003800
H	-7.22253300	0.56111900	-0.58442100
H	-7.07992600	0.52136900	1.17640300
H	-6.70918500	2.01412600	0.30625100
C	5.41478400	-3.76287800	-0.73008300

H	5.66001400	-4.36418500	0.14950100
H	5.42342200	-4.42844200	-1.60272000
H	6.20196200	-3.01782900	-0.88087500
O	-1.21580700	-2.82271400	-0.62379900
H	-0.50899600	-3.45116000	-0.86027900

$Ru^{III}(OOH)(EtOtpy)(pic)_2$

O	1.71854900	4.60423000	1.16725800
N	0.34712500	0.88076800	0.21020900
N	-0.13521300	-0.13276900	-2.15341000
N	-0.26743700	-1.01248300	1.93061800
C	0.60548300	1.72957000	-0.80647000
C	1.07728900	3.01556000	-0.55394000
C	1.27475000	3.40540100	0.78328600
C	0.99572200	2.49469900	1.82350700
C	0.53111800	1.22919000	1.50551500
C	0.34166800	1.15096700	-2.14833300
C	0.54426800	1.83708200	-3.34450200
C	0.25268300	1.20734100	-4.55478300
C	-0.24144000	-0.09417800	-4.54299900
C	-0.42280000	-0.73306900	-3.31787700
C	0.19054300	0.15713900	2.47176200
C	0.32087200	0.29917600	3.85217900
C	-0.01726700	-0.76383700	4.68922700
C	-0.47861100	-1.95041600	4.12621900
C	-0.58923600	-2.03304900	2.74012200
C	2.02873600	5.61313200	0.17360200
C	2.50175500	6.84599500	0.91792600
H	1.28450400	3.69394200	-1.36893800

H	1.15273100	2.81321100	2.84610500
H	0.92425500	2.85132700	-3.33678800
H	0.40915300	1.73295800	-5.49063300
H	-0.48463400	-0.61762500	-5.46041500
H	-0.81519100	-1.74132500	-3.24593600
H	0.68337100	1.22900400	4.27301800
H	0.08083300	-0.66098200	5.76455400
H	-0.75253700	-2.80257600	4.73733000
H	-0.94465200	-2.93526600	2.25519500
H	2.80596400	5.22415400	-0.49346900
H	1.12645200	5.81966500	-0.41255400
H	3.39770100	6.62565900	1.50624600
H	2.74514000	7.63248800	0.19669900
H	1.72138900	7.21937400	1.58798400
C	-3.31231500	-1.14350000	0.48282600
C	-2.83857600	0.84578800	-0.58597400
C	-4.64861400	-0.79691900	0.61641700
H	-2.95039900	-2.09680400	0.84544200
C	-4.16102900	1.25649600	-0.48942300
H	-2.10500700	1.48243500	-1.06220300
H	-5.32607800	-1.49286300	1.10051000
H	-4.44259900	2.22104100	-0.89920600
C	1.90113800	-3.01692400	0.24452500
C	2.74588100	-1.08212200	-0.68564200
C	3.16303500	-3.59201800	0.28778200
H	1.03215000	-3.56461100	0.58759300
C	4.03583000	-1.59471500	-0.67468300
H	2.56024400	-0.08678600	-1.06699300
H	3.27019300	-4.59479700	0.68862300

H	4.84769400	-0.98181900	-1.05265200
Ru	-0.34832400	-0.99700800	-0.21624600
N	1.68045000	-1.77365300	-0.23278100
N	-2.40411200	-0.33836800	-0.10933300
C	-5.10980500	0.43266000	0.12821900
C	4.27665400	-2.88262200	-0.18041900
C	-6.54590900	0.85464600	0.28116500
H	-7.21848200	-0.00782500	0.25694200
H	-6.69083600	1.35736500	1.24610800
H	-6.84120000	1.55652400	-0.50391800
C	5.65616700	-3.48333600	-0.17640000
H	5.77075300	-4.21214200	0.63112400
H	5.84225000	-4.00729100	-1.12279800
H	6.42562300	-2.71310400	-0.07057000
O	-0.88235100	-2.84303600	-0.61760800
O	-1.98501600	-2.97867600	-1.51027400
H	-2.00562400	-3.94843900	-1.63071000

$Ru^{IV}(O)(EtOtpy)(pic)_2$

O	-0.09305300	4.85563000	0.97258900
N	-0.01849600	0.86075900	0.18734700
N	-0.22180800	-0.37302900	-2.12207900
N	0.27134900	-1.05625400	1.96658000
C	-0.21606100	1.69104100	-0.85450400
C	-0.25439900	3.06886700	-0.66149200
C	-0.07913600	3.56444900	0.64578100
C	0.12861500	2.66654200	1.71614600
C	0.15230900	1.30780000	1.45264000
C	-0.34921600	0.98955300	-2.15861500

C	-0.56968700	1.64528500	-3.36802500
C	-0.65625000	0.90531800	-4.54767300
C	-0.51530400	-0.47819900	-4.49466100
C	-0.29900700	-1.08229300	-3.25805900
C	0.32492400	0.22220300	2.45180600
C	0.50984400	0.45403400	3.81312700
C	0.63804000	-0.62732100	4.68481600
C	0.57415900	-1.92187700	4.17691700
C	0.38967900	-2.09537300	2.80735200
C	-0.30358200	5.86393900	-0.04983900
C	-0.28398800	7.21286300	0.64081000
H	-0.40940500	3.74086200	-1.49297000
H	0.25980100	3.07002500	2.71198400
H	-0.67304200	2.72296900	-3.39534000
H	-0.82868800	1.40961000	-5.49235000
H	-0.57015700	-1.09094800	-5.38682400
H	-0.18164400	-2.15495500	-3.15958200
H	0.55136000	1.46760100	4.19252900
H	0.78247500	-0.45444900	5.74578700
H	0.66408300	-2.79055700	4.81857100
H	0.33187300	-3.08031400	2.35939900
H	0.49598500	5.78125000	-0.79393000
H	-1.26645800	5.67529300	-0.53672900
H	0.67841300	7.38484500	1.13219600
H	-0.43958700	8.00095600	-0.10257800
H	-1.08031900	7.27904300	1.38828500
C	-2.76108500	-2.36178500	-0.59746200
C	-2.87061900	-0.46545500	0.71949000
C	-4.13807000	-2.52139000	-0.54327000

H	-2.13500000	-3.06234000	-1.13597300
C	-4.25088300	-0.56774200	0.81686900
H	-2.34815500	0.34428300	1.21072200
H	-4.58969800	-3.35814500	-1.06618000
H	-4.79190500	0.17580400	1.39290400
C	2.91144600	-2.25122200	-0.02245600
C	2.89015800	-0.06299500	-0.76685000
C	4.29460800	-2.30097800	-0.09702600
H	2.33461300	-3.11182900	0.29262600
C	4.27501100	-0.04549900	-0.86753800
H	2.31367700	0.81494600	-1.02545000
H	4.80224100	-3.21856900	0.18241700
H	4.76402900	0.86050700	-1.20980700
Ru	0.04055600	-1.20442200	-0.16135900
N	2.20547200	-1.14581300	-0.34870000
N	-2.12291400	-1.34508700	0.02153400
C	-4.92625700	-1.61504800	0.17753000
C	5.01937400	-1.18120200	-0.52907300
C	-6.41872500	-1.77147600	0.28242300
H	-6.82318900	-2.32265400	-0.57104000
H	-6.67560900	-2.33124300	1.19094500
H	-6.91655100	-0.79929600	0.34531600
C	6.51912600	-1.21184900	-0.64158200
H	6.96764600	-1.72853800	0.21290800
H	6.81935800	-1.75728900	-1.54520500
H	6.93689600	-0.20359700	-0.70378300
O	0.09769300	-2.98486300	-0.46725700

$Ru^{IV}(OH)(EtOtpy)(pic)_2$

O	2.56161900	4.08127500	1.08742900
N	0.39983800	0.74910500	0.21807600
N	-0.42314200	-0.08278000	-2.11029900
N	-0.39677200	-1.03139800	1.93819700
C	0.69968000	1.59241300	-0.80874500
C	1.42464600	2.74699900	-0.58012100
C	1.85972400	3.02422800	0.73705700
C	1.53325600	2.12841700	1.78739700
C	0.80152400	0.99872300	1.50161100
C	0.18333600	1.14247100	-2.11680900
C	0.28564800	1.87263800	-3.29676500
C	-0.24357900	1.34541800	-4.47634200
C	-0.86288900	0.09983700	-4.44995500
C	-0.93764400	-0.59034100	-3.24170200
C	0.32481400	-0.00337200	2.47535800
C	0.52078300	0.07594900	3.85098900
C	-0.04199000	-0.89693700	4.67842100
C	-0.79966200	-1.92051500	4.11538800
C	-0.96023500	-1.95730800	2.73254600
C	2.95050200	5.09064500	0.10384200
C	3.73747400	6.15128000	0.84373400
H	1.65685300	3.41845400	-1.39370000
H	1.86336500	2.36491200	2.79053900
H	0.76513600	2.84352300	-3.30080200
H	-0.16932600	1.90758800	-5.40050300
H	-1.28495200	-0.34644200	-5.34247500
H	-1.39846500	-1.56769100	-3.17276200
H	1.09546900	0.88939100	4.27609300
H	0.10474000	-0.84417200	5.75145400

H	-1.26464200	-2.68645700	4.72447200
H	-1.53460500	-2.73343200	2.24108000
H	3.55148000	4.60031700	-0.66771800
H	2.03840600	5.49636000	-0.34405900
H	4.63507900	5.72405000	1.30011400
H	4.04413900	6.92485100	0.13288900
H	3.12742700	6.61791800	1.62254600
C	-3.54390300	-0.78578500	-0.68896100
C	-2.80652000	0.88639700	0.74478400
C	-4.84328700	-0.30849900	-0.62656500
H	-3.29741200	-1.66661000	-1.26756300
C	-4.08434700	1.41164200	0.84392000
H	-1.98251300	1.34494500	1.27427700
H	-5.61495800	-0.82262900	-1.18950900
H	-4.24135500	2.28770700	1.46394700
C	1.80186900	-2.83376100	0.57794400
C	2.18140500	-1.76134000	-1.44498800
C	3.01451700	-3.49586600	0.49245200
H	1.14895900	-2.99616200	1.42399400
C	3.40763700	-2.38925200	-1.58491600
H	1.82934700	-1.07738900	-2.20304000
H	3.29517200	-4.17445300	1.29073700
H	4.00573100	-2.17304600	-2.46357900
Ru	-0.54126200	-1.00129800	-0.18472600
N	1.37842300	-1.96804800	-0.37479900
N	-2.53120400	-0.19780900	-0.01395000
C	-5.14812800	0.81665500	0.15161900
C	3.85972800	-3.28552700	-0.60661400
C	-6.55084000	1.34397200	0.26050400

H	-7.13427500	1.11177800	-0.63458300
H	-7.05744600	0.87947000	1.11640600
H	-6.55759400	2.42555700	0.42184100
C	5.17099100	-4.00473300	-0.73962400
H	5.61968800	-4.19583000	0.23925400
H	5.01301500	-4.97678000	-1.22484000
H	5.87614700	-3.43850400	-1.35381100
O	-1.33294700	-2.69523400	-0.60019500
H	-0.74351200	-3.41858000	-0.89395900

$Ru^{V}(O)(EtOtpy)(pic)_2$

O	3.02977100	3.83955900	0.98852200
N	0.46800900	0.74408500	0.20120300
N	-0.33913600	-0.11134400	-2.07748600
N	-0.61635800	-0.76313000	1.98249600
C	0.93310200	1.47662200	-0.83543700
C	1.79640800	2.54689500	-0.64962100
C	2.19936900	2.86571000	0.66276000
C	1.68820400	2.10308000	1.73662700
C	0.82928600	1.05597900	1.47752400
C	0.43076300	1.00985800	-2.13182300
C	0.67618000	1.64057000	-3.34872300
C	0.12179600	1.12203700	-4.51656400
C	-0.66907900	-0.02018700	-4.43887700
C	-0.88299600	-0.61741000	-3.20142900
C	0.19469900	0.20709600	2.48767800
C	0.34411700	0.36055200	3.86270600
C	-0.34408000	-0.48926300	4.72687100
C	-1.17520600	-1.47084400	4.19522000

C	-1.29110000	-1.58047300	2.81326800
C	3.62768500	4.68165600	-0.04088700
C	4.53718800	5.66597800	0.66459600
H	2.15569600	3.11165300	-1.49723700
H	1.98340100	2.36250300	2.74516800
H	1.28601500	2.53438600	-3.38003300
H	0.30561400	1.60826500	-5.46801800
H	-1.12230100	-0.46382400	-5.31742900
H	-1.48465200	-1.50966300	-3.09919000
H	0.98654300	1.13888500	4.25471500
H	-0.23401600	-0.37819700	5.79982800
H	-1.73453000	-2.15188600	4.82524800
H	-1.92564700	-2.33765200	2.36928700
H	4.18294600	4.03877800	-0.73112400
H	2.82185300	5.18712800	-0.58219300
H	5.32720500	5.14300700	1.21162500
H	5.00395400	6.31807400	-0.08010300
H	3.97093200	6.28778600	1.36419000
C	-3.72075400	-0.68897900	-0.17171500
C	-2.66403000	1.37463600	0.01430500
C	-4.96732700	-0.08845100	-0.12413300
H	-3.61895300	-1.76205300	-0.27214700
C	-3.88481100	2.03050400	0.06575300
H	-1.73829000	1.92974300	0.07117500
H	-5.84966200	-0.71636800	-0.18908600
H	-3.89130400	3.11109100	0.15816500
C	1.05612400	-3.51389300	-0.01564400
C	2.38918600	-1.66480400	-0.44190600
C	2.15359700	-4.35779300	-0.05796100

H	0.06316800	-3.90369400	0.17186500
C	3.52525500	-2.45760000	-0.50002300
H	2.46655000	-0.59733500	-0.58546800
H	2.00062000	-5.41955000	0.10275900
H	4.48096800	-1.98265600	-0.69404500
Ru	-0.69436200	-1.01754500	-0.17085100
N	1.16425800	-2.17709400	-0.20175300
N	-2.57743600	0.03350200	-0.10153400
C	-5.08001700	1.30471800	-0.00127400
C	3.43215900	-3.84250100	-0.31101600
C	-6.42186500	1.97761100	0.06527000
H	-7.07962800	1.61464200	-0.73131000
H	-6.91153700	1.74758400	1.01949800
H	-6.33054900	3.06304500	-0.01872700
C	4.63785700	-4.73443100	-0.40225600
H	4.51260400	-5.63624100	0.20307700
H	4.78729900	-5.05116000	-1.44256400
H	5.54434200	-4.21222300	-0.08385700
O	-1.39631400	-2.31024200	-1.07937500

$Ru^{II}(H_2O)$(EtOtpy)(bpy)

Ru	0.68890200	0.00802500	-0.53623800
O	-5.35353800	0.54737500	0.18379100
N	-1.30324100	0.14778200	-0.22838200
N	0.10687600	-2.05118300	-0.50793000
N	0.41278700	2.12996100	-0.46445900
N	2.80801900	-0.14613000	-0.73842600
N	1.31387300	-0.06059900	1.44597500
C	-2.04027600	-0.98077000	-0.19082200

C	-3.42698700	-0.91852700	-0.04981000
C	-4.03578100	0.34401800	0.04294700
C	-3.24180900	1.50582100	-0.02146600
C	-1.86796300	1.37751300	-0.16313400
C	-1.24019600	-2.22380900	-0.32028900
C	-1.79591100	-3.50193700	-0.26154500
C	-0.97345300	-4.61957500	-0.39680400
C	0.39320000	-4.43412300	-0.58930400
C	0.89048700	-3.13401700	-0.63791000
C	-0.89459700	2.49217000	-0.26907800
C	-1.26026600	3.83554700	-0.18334200
C	-0.28512100	4.82495600	-0.30021200
C	1.04038500	4.44769700	-0.50116200
C	1.34477100	3.09081500	-0.57631400
C	-6.24945000	-0.58580300	0.25318400
C	-7.65442700	-0.04039300	0.42049800
C	3.49316800	-0.20919700	0.43766500
C	4.88798900	-0.30959300	0.45561300
C	5.58995700	-0.34765400	-0.74552700
C	4.88111300	-0.28491900	-1.94487300
C	3.49549100	-0.18497000	-1.89484100
C	2.65919400	-0.16363900	1.65336800
C	3.17902000	-0.22036900	2.94987200
C	2.32059300	-0.17053200	4.04378100
C	0.94905000	-0.06377600	3.81941100
C	0.48873500	-0.01211700	2.50933700
H	-4.01699900	-1.82321300	-0.02027200
H	-3.73004600	2.47056500	0.03431400
H	-2.86089100	-3.62866600	-0.10962000

H	-1.39857100	-5.61661200	-0.35132000
H	1.07098400	-5.27295300	-0.69977400
H	1.94670500	-2.94025000	-0.78466500
H	-2.29628100	4.10997800	-0.02556300
H	-0.56139800	5.87201500	-0.23427300
H	1.83212300	5.18186100	-0.59811700
H	2.36182500	2.74935100	-0.73049900
H	-6.15549200	-1.17143000	-0.66860300
H	-5.96189200	-1.21604400	1.10265200
H	-7.92956600	0.59300600	-0.42846200
H	-8.36299200	-0.87285300	0.47599000
H	-7.73679700	0.54700600	1.34017600
H	5.42459400	-0.35763200	1.39483200
H	6.67203300	-0.42528800	-0.74231600
H	5.38360900	-0.31199200	-2.90508400
H	2.90502400	-0.13354700	-2.80180400
H	4.24707500	-0.30316100	3.10613400
H	2.71867400	-0.21424700	5.05193000
H	0.23932100	-0.02088400	4.63768800
H	-0.56802800	0.07058000	2.29027000
O	0.14280700	0.07756400	-2.69963800
H	0.40956400	-0.70248900	-3.21588500
H	0.49190200	0.84855500	-3.17935600

$Ru^{III}(H_2O)$(EtOtpy)(bpy)

Ru	0.68679800	0.00161200	-0.56841700
O	-5.38317700	0.54706900	0.11554900
N	-1.33460000	0.14571900	-0.21402400
N	0.08130000	-2.02527100	-0.33646700

N	0.38693200	2.09280500	-0.30771200
N	2.78174300	-0.14928200	-0.85082500
N	1.39391700	-0.06010100	1.38493200
C	-2.07384400	-0.98111900	-0.16805500
C	-3.45832300	-0.91984400	-0.05303800
C	-4.07328700	0.34497400	0.00656100
C	-3.27314100	1.50637700	-0.04245600
C	-1.90035700	1.37498300	-0.15563300
C	-1.26912200	-2.21728700	-0.22849500
C	-1.79443100	-3.50404400	-0.15762200
C	-0.93156200	-4.59985500	-0.19147600
C	0.44127900	-4.38758100	-0.29077900
C	0.91374600	-3.08059200	-0.35679900
C	-0.92169200	2.47877400	-0.20307800
C	-1.25448200	3.82797400	-0.12512100
C	-0.24158100	4.78654200	-0.15296500
C	1.08626500	4.37738700	-0.25129100
C	1.36419600	3.01626200	-0.32206500
C	-6.29181600	-0.58469600	0.18213000
C	-7.69511900	-0.02645800	0.30534400
C	3.52367600	-0.20121500	0.29344800
C	4.91391500	-0.29507700	0.23481100
C	5.54402100	-0.33874900	-1.00742100
C	4.77466100	-0.28873800	-2.16950300
C	3.39412600	-0.19370200	-2.05014800
C	2.74596900	-0.15056200	1.53997200
C	3.30835600	-0.18950500	2.81633500
C	2.48117300	-0.13565300	3.93492400
C	1.10224100	-0.04322800	3.75809300

C	0.59410600	-0.00746400	2.46532500
H	-4.04269500	-1.82723500	-0.00669500
H	-3.75558200	2.47372500	0.01428100
H	-2.86347800	-3.65402100	-0.07342200
H	-1.33274300	-5.60579800	-0.13519600
H	1.14538400	-5.21071300	-0.31617500
H	1.97177300	-2.86560600	-0.43753800
H	-2.29101500	4.12982600	-0.04135900
H	-0.49201300	5.84009500	-0.09336900
H	1.90216000	5.08994200	-0.27309400
H	2.37986500	2.65023900	-0.40362200
H	-6.17409400	-1.18187800	-0.72837100
H	-6.02459200	-1.19689000	1.05003000
H	-7.94615700	0.59178900	-0.56175000
H	-8.40805400	-0.85521300	0.35864400
H	-7.79609300	0.57679000	1.21255800
H	5.50275100	-0.33429600	1.14218700
H	6.62470200	-0.41154600	-1.06319700
H	5.22677500	-0.32119900	-3.15354000
H	2.75207600	-0.14924800	-2.92054600
H	4.38142000	-0.26058300	2.93734400
H	2.91170500	-0.16494100	4.92977500
H	0.42077100	0.00203100	4.59914600
H	-0.46912300	0.06617600	2.28105900
O	-0.04726800	0.08024400	-2.65600800
H	0.09703700	-0.72654500	-3.18435200
H	0.28478800	0.82115800	-3.19607800

Ru^{III}(OH)(EtOtpy)(bpy)

Ru	0.66204900	0.00836900	-0.58450900
O	-5.37678700	0.56156500	0.14770100
N	-1.33857000	0.15290400	-0.29343000
N	0.08347800	-2.04561100	-0.44052900
N	0.40079700	2.12347100	-0.37543400
N	2.77143500	-0.13386700	-0.81615000
N	1.36881400	-0.08618700	1.44940300
C	-2.07427300	-0.97545500	-0.23312500
C	-3.45825800	-0.91027700	-0.08374800
C	-4.06482800	0.35531200	0.00474400
C	-3.26583500	1.51613300	-0.04852200
C	-1.89482400	1.38450600	-0.19872000
C	-1.26979900	-2.21747600	-0.31699000
C	-1.81788100	-3.49787400	-0.26284800
C	-0.97845300	-4.60915900	-0.33519500
C	0.39558300	-4.41931900	-0.45922700
C	0.88742000	-3.11779700	-0.50769100
C	-0.91179800	2.49226100	-0.24975300
C	-1.26370100	3.83807300	-0.16345600
C	-0.26767100	4.81310500	-0.20617600
C	1.06360000	4.42357400	-0.33201500
C	1.35626400	3.06509300	-0.41297900
C	-6.28203500	-0.56783700	0.21959000
C	-7.68280300	-0.01135600	0.38135700
C	3.50148300	-0.20151600	0.32957300
C	4.89571500	-0.28704800	0.27909300
C	5.53585400	-0.30409800	-0.95783700
C	4.77262000	-0.23576300	-2.12275300
C	3.38894100	-0.15109500	-2.00951800

C	2.72149000	-0.17872500	1.58565600
C	3.30391900	-0.24902500	2.85385400
C	2.49329400	-0.22445700	3.98578900
C	1.11112300	-0.13018900	3.83242400
C	0.59044600	-0.06346200	2.54535000
H	-4.04788800	-1.81412200	-0.03323500
H	-3.74835400	2.48175000	0.03324200
H	-2.88805400	-3.63181300	-0.16526500
H	-1.39822000	-5.60849400	-0.29415700
H	1.08263200	-5.25530600	-0.51883300
H	1.94725700	-2.91678300	-0.60717000
H	-2.30305800	4.12575400	-0.06407300
H	-0.53360100	5.86273200	-0.14054100
H	1.86787200	5.14904200	-0.36853200
H	2.37501100	2.71105900	-0.51552500
H	-6.18750800	-1.15575000	-0.70016300
H	-5.99870800	-1.19408100	1.07289100
H	-7.95188500	0.61966200	-0.47116700
H	-8.39635000	-0.83933800	0.43896800
H	-7.76308700	0.58013600	1.29847800
H	5.47988600	-0.33975900	1.18905400
H	6.61758300	-0.37028400	-1.00715300
H	5.23277700	-0.24703100	-3.10412700
H	2.73494300	-0.09448200	-2.87263600
H	4.37825900	-0.32300100	2.96205000
H	2.93899500	-0.27889400	4.97333800
H	0.44337400	-0.10834000	4.68572300
H	-0.47687000	0.01068300	2.37499900
O	0.41157400	0.05121400	-2.52654300

H		-0.52870000	0.07442900	-2.78531500

$Ru^{III}(OOH)(EtOtpy)(bpy)$

Ru	-1	0.68036700	0.02069900	-0.53344800
O	0	-5.34799400	0.47385800	0.31455800
O	-1	0.53806900	0.09514200	-2.32553000
N	0	-1.31632600	0.12776800	-0.20405600
N	0	0.12653500	-2.06436200	-0.46028200
N	0	0.39283500	2.12441500	-0.32386100
N	0	2.81700500	-0.13925900	-0.70390700
N	0	1.35765500	-0.07655600	1.53461200
C	0	-2.03451900	-1.01148400	-0.16345000
C	0	-3.41592100	-0.96671300	0.01030000
C	0	-4.03602000	0.28903300	0.14403900
C	0	-3.25344600	1.46090000	0.10545100
C	0	-1.88406000	1.34966200	-0.07303000
C	0	-1.22121100	-2.24641600	-0.30365400
C	0	-1.76286500	-3.53019100	-0.26936600
C	0	-0.92190800	-4.63585600	-0.39783800
C	0	0.44645500	-4.43616300	-0.55709500
C	0	0.93061800	-3.13029500	-0.58256300
C	0	-0.91893800	2.47120700	-0.13994500
C	0	-1.28830500	3.80988900	-0.01793900
C	0	-0.31173100	4.80233800	-0.08905000
C	0	1.01857600	4.43590000	-0.27788700
C	0	1.32921400	3.08340000	-0.38949800
C	0	-6.23856900	-0.66810500	0.36672400
C	0	-7.64321500	-0.13347900	0.56484400
C	0	3.51100100	-0.23132600	0.46272300

C	0	4.90422600	-0.34839000	0.45398000
C	0	5.58504600	-0.37236400	-0.76034900
C	0	4.86088900	-0.27891500	-1.94759500
C	0	3.47716200	-0.16320200	-1.87478800
C	0	2.70225100	-0.19877300	1.70137100
C	0	3.25706200	-0.28286000	2.98171600
C	0	2.42386800	-0.24017300	4.09655300
C	0	1.04838000	-0.11229700	3.91318700
C	0	0.55857800	-0.03301500	2.61430100
H	0	-3.99556700	-1.87772400	0.04241600
H	0	-3.74698700	2.41809700	0.21531000
H	0	-2.82894600	-3.67275500	-0.14337900
H	0	-1.33688200	-5.63774500	-0.37211700
H	0	1.13512900	-5.26664100	-0.66013400
H	0	1.98674100	-2.92176300	-0.70559700
H	0	-2.32712500	4.07810800	0.13007200
H	0	-0.59134800	5.84643700	0.00283200
H	0	1.80918900	5.17465000	-0.33962700
H	0	2.34842600	2.74764300	-0.53883300
H	0	-6.15105500	-1.22790400	-0.57109400
H	0	-5.93544400	-1.31546500	1.19724200
H	0	-7.93223500	0.51842000	-0.26516500
H	0	-8.34622000	-0.97124900	0.60896000
H	0	-7.71669100	0.43055800	1.49966600
H	0	5.45738500	-0.42018400	1.38164000
H	0	6.66602600	-0.46283400	-0.77489900
H	0	5.34952300	-0.29347400	-2.91503200
H	0	2.86113100	-0.08462000	-2.76350100
H	0	4.32674900	-0.38075500	3.11383600

H	0	2.84736200	-0.30515700	5.09322600
H	0	0.36261400	-0.07401400	4.75161100
H	0	-0.50319800	0.06591400	2.42246000
O	0	-0.65087700	0.69201200	-2.91563200
H	0	-0.36792900	0.73568100	-3.84974400

Ru^{IV}(O)(EtOtpy)(bpy)

Ru	0.66412600	0.02248600	-0.65616700
O	-5.34938100	0.53443100	0.17648600
O	0.51575300	0.07069200	-2.44859800
N	-1.32768900	0.15370500	-0.31678000
N	0.10259600	-2.05025900	-0.55886500
N	0.39920400	2.14740900	-0.47484800
N	2.80928600	-0.12752100	-0.74120000
N	1.30601800	-0.07471800	1.46931600
C	-2.05112200	-0.98395600	-0.26169700
C	-3.43094100	-0.92624300	-0.09361800
C	-4.04228000	0.33790500	0.01493200
C	-3.25205500	1.50637800	-0.04427000
C	-1.88427700	1.38541900	-0.21486000
C	-1.24424300	-2.22369800	-0.38488800
C	-1.79006300	-3.50384300	-0.32078100
C	-0.95404200	-4.61471200	-0.43879100
C	0.41251000	-4.42267100	-0.61788300
C	0.90242100	-3.11938600	-0.67236300
C	-0.91096300	2.50163900	-0.29739700
C	-1.27469500	3.84283100	-0.19741600
C	-0.29232200	4.82948100	-0.28443300
C	1.03561700	4.45439400	-0.46723300

C	1.34055300	3.09819800	-0.55722600
C	-6.25388000	-0.59866300	0.24859500
C	-7.65304200	-0.04429400	0.42738600
C	3.47930000	-0.21050300	0.43928400
C	4.87323200	-0.31080800	0.45662400
C	5.57750200	-0.32581800	-0.74450200
C	4.87615400	-0.24065200	-1.94568300
C	3.49018400	-0.14232000	-1.90141600
C	2.64673400	-0.18540600	1.66225700
C	3.17786100	-0.26805300	2.95223800
C	2.32205500	-0.23516500	4.05050800
C	0.94964200	-0.11942500	3.84045600
C	0.48482100	-0.04218100	2.53194500
H	-4.01738000	-1.83231400	-0.04937700
H	-3.74220700	2.46746500	0.04330000
H	-2.85506500	-3.63974800	-0.17962300
H	-1.37200000	-5.61440900	-0.39038300
H	1.09710900	-5.25711200	-0.71509300
H	1.95755600	-2.91779500	-0.81230000
H	-2.31199600	4.11901100	-0.05419400
H	-0.56732800	5.87607700	-0.20948100
H	1.82966200	5.18823300	-0.54083500
H	2.35812800	2.75562400	-0.70167900
H	-6.16583200	-1.17759700	-0.67704200
H	-5.96013600	-1.22914700	1.09481500
H	-7.93007300	0.59342500	-0.41748500
H	-8.36465600	-0.87398000	0.48376200
H	-7.72609500	0.53875200	1.35041200
H	5.40880600	-0.37642300	1.39485200

H	6.65952800	-0.40309200	-0.73778400
H	5.38344500	-0.24891000	-2.90344600
H	2.88785900	-0.07210900	-2.79972800
H	4.24551500	-0.35721700	3.10520200
H	2.72643100	-0.29894500	5.05512200
H	0.24682700	-0.08920000	4.66492900
H	-0.57448300	0.04804700	2.32235600

Ru^{IV}(OH)(EtOtpy)(bpy)

Ru	0.65181000	-0.00558000	-0.63731200
O	-5.32882100	0.55761700	0.13064000
O	0.49282600	0.01769500	-2.53687200
N	-1.31413900	0.14507000	-0.27029500
N	0.11034800	-2.04669300	-0.46138000
N	0.43047100	2.09506200	-0.43834500
N	2.78484700	-0.15095500	-0.75467600
N	1.26434800	-0.05928500	1.41799400
C	-2.04917700	-0.99483900	-0.21639500
C	-3.42568800	-0.92464900	-0.07628800
C	-4.03169400	0.34965300	0.00436500
C	-3.22914300	1.51771800	-0.04792900
C	-1.86398800	1.38777900	-0.19002300
C	-1.24271100	-2.22926900	-0.31350100
C	-1.77384900	-3.51242000	-0.24544900
C	-0.91892300	-4.61356200	-0.32451800
C	0.45100000	-4.40999000	-0.46852900
C	0.93319100	-3.10564200	-0.53011800
C	-0.87569700	2.48365100	-0.26771800
C	-1.19943400	3.83193300	-0.16687000

C	-0.18462000	4.78797400	-0.23999900
C	1.13546300	4.37699400	-0.40823600
C	1.40765800	3.01606100	-0.50028500
C	-6.26512000	-0.56040700	0.18910600
C	-7.65509400	0.02980000	0.30206700
C	3.45335200	-0.20069600	0.43018500
C	4.84537800	-0.29231000	0.45152800
C	5.54701500	-0.33483200	-0.75139300
C	4.84600600	-0.28661000	-1.95474100
C	3.45942100	-0.19337300	-1.91873500
C	2.60880700	-0.15134500	1.63500800
C	3.11055100	-0.19722400	2.93559000
C	2.23247500	-0.14755700	4.01540200
C	0.86249600	-0.05256000	3.77719600
C	0.41605300	-0.01101100	2.46343700
H	-4.01930300	-1.82602800	-0.03221000
H	-3.71479800	2.48259300	0.02035600
H	-2.84029300	-3.65851900	-0.12856200
H	-1.32576400	-5.61721600	-0.27033600
H	1.14621400	-5.23826800	-0.53474100
H	1.98910000	-2.89708500	-0.64643400
H	-2.22912100	4.13790700	-0.03080200
H	-0.42931200	5.84160500	-0.16267100
H	1.95013200	5.08847800	-0.46984900
H	2.41602700	2.64660800	-0.63654900
H	-6.14838900	-1.15370900	-0.72303400
H	-6.01105200	-1.17428900	1.05867900
H	-7.88537000	0.65370800	-0.56650500
H	-8.38468900	-0.78468500	0.34759000

H	-7.75072600	0.63257000	1.20990500
H	5.38007200	-0.33082100	1.39163100
H	6.62914800	-0.40596100	-0.74382000
H	5.35333700	-0.31995400	-2.91160600
H	2.85871800	-0.15362100	-2.81910500
H	4.17629400	-0.27121300	3.10754900
H	2.61743800	-0.18211000	5.02861200
H	0.14287900	-0.01043400	4.58601300
H	-0.63874200	0.06462500	2.23599400
H	0.23302700	0.85685600	-2.97057000

Ru^{V}(O)(EtOtpy)(bpy)

Ru	0.65133000	0.00576200	-0.74108200
O	-5.26113900	0.59838300	0.15423300
O	0.85981500	-0.03931200	-2.45378000
N	-1.26944200	0.16204200	-0.35535700
N	0.10905700	-2.05764900	-0.62978100
N	0.47123300	2.11949800	-0.53336300
N	2.82527900	-0.12882900	-0.61449300
N	1.15018400	-0.11082800	1.41605800
C	-2.01898700	-0.98473400	-0.29517600
C	-3.38512700	-0.89746400	-0.12958200
C	-3.97835400	0.38551700	-0.01088700
C	-3.16707600	1.55234900	-0.06871400
C	-1.81144200	1.41964000	-0.23986700
C	-1.23406700	-2.22775200	-0.43001400
C	-1.78108200	-3.50473300	-0.36020500
C	-0.94477400	-4.61445200	-0.50292200
C	0.41678500	-4.42218800	-0.71069500

C	0.91244200	-3.11972800	-0.76819100
C	-0.82302700	2.51228600	-0.32609900
C	-1.14091200	3.86009400	-0.20132700
C	-0.12354300	4.81238800	-0.29496800
C	1.18671900	4.39575700	-0.50816800
C	1.44968300	3.03217600	-0.62149700
C	-6.21818500	-0.50886700	0.23859100
C	-7.58874100	0.10506800	0.42525500
C	3.39741900	-0.21665400	0.61282300
C	4.78420900	-0.30036100	0.74597500
C	5.58641300	-0.28886000	-0.39150400
C	4.98461500	-0.19356800	-1.64404100
C	3.60005400	-0.11527000	-1.71933500
C	2.46287100	-0.21012500	1.74675200
C	2.86322500	-0.29783300	3.08040600
C	1.90200100	-0.28532300	4.08708400
C	0.55794900	-0.18338800	3.73752700
C	0.22080900	-0.09873700	2.39259000
H	-3.99088400	-1.79107600	-0.09565600
H	-3.64679300	2.51857100	0.01779800
H	-2.84289000	-3.64074900	-0.19805200
H	-1.36136100	-5.61411200	-0.45123400
H	1.09867700	-5.25581700	-0.82837600
H	1.96418000	-2.92028300	-0.92971400
H	-2.16498300	4.16931900	-0.03412000
H	-0.36049200	5.86621900	-0.19957300
H	2.00388800	5.10273800	-0.58638700
H	2.45197600	2.65987400	-0.78940400
H	-6.14876300	-1.08454700	-0.68878000

H	-5.93114200	-1.13717600	1.08655600
H	-7.84810400	0.74701600	-0.42141600
H	-8.32843000	-0.69886900	0.49068800
H	-7.63172600	0.69203000	1.34714700
H	5.23508900	-0.37148400	1.72683000
H	6.66471700	-0.35309000	-0.29646700
H	5.56599100	-0.18135300	-2.55838800
H	3.08745200	-0.04287000	-2.66985400
H	3.91215800	-0.37628800	3.33414100
H	2.20236900	-0.35406000	5.12684500
H	-0.22819700	-0.16685500	4.48300900
H	-0.81410300	-0.01431300	2.09409100

$Ru^{II}(H_2O)$(EtOtpy)(QC)

N	1.64924100	-0.24047800	-0.29808000
N	0.03221400	-2.31311600	-0.21503400
N	0.11509600	1.84079600	-0.74133100
C	2.27100500	-1.41361400	-0.03049800
C	3.64005100	-1.44141300	0.19510900
C	4.36931700	-0.23746300	0.15128000
C	3.69614900	0.96792000	-0.10644900
C	2.31858400	0.92993200	-0.32847300
C	1.35914200	-2.58343600	-0.00293400
C	1.79896600	-3.88854200	0.22225700
C	0.87789000	-4.93459500	0.23613900
C	-0.46940300	-4.65091000	0.02232100
C	-0.84664800	-3.32871700	-0.19898200
C	1.45449000	2.10436600	-0.60230600
C	1.94449100	3.40579500	-0.72078600

C	1.06451100	4.45546200	-0.97951600
C	-0.29399500	4.17921200	-1.11748800
C	-0.72329100	2.86049900	-0.99193000
H	4.17130700	-2.36031900	0.40953100
H	4.22480500	1.90998500	-0.12478900
H	2.85133900	-4.08866200	0.38482600
H	1.21128600	-5.95218200	0.41103700
H	-1.22188800	-5.43157600	0.02545800
H	-1.88289300	-3.05888400	-0.36902400
H	3.00456300	3.60274100	-0.61518900
H	1.43850000	5.46971100	-1.07204500
H	-1.01596600	4.96269400	-1.31873000
H	-1.77003600	2.59707500	-1.09195700
Ru	-0.34910900	-0.22932200	-0.51769900
C	-4.38875600	-0.44189800	-2.16175800
C	-5.25637300	0.00424800	-1.19713500
C	-4.75969400	0.34874500	0.08339300
C	-3.35009200	0.21903600	0.35748000
C	-3.02219500	-0.51114800	-1.84592900
H	-6.70548400	0.90846000	0.83280100
H	-4.72243800	-0.72328300	-3.15430600
H	-6.32064100	0.09861500	-1.39301900
C	-5.65187500	0.81591900	1.08105500
C	-2.88476100	0.52250400	1.68395000
H	-2.30894300	-0.82173200	-2.59992300
C	-3.81399900	0.97497300	2.61341200
C	-5.18206800	1.13926000	2.32984600
H	-3.43889500	1.19038900	3.60643300
H	-5.85439300	1.50265600	3.10093600

C	-1.47754500	0.35628000	2.26167400
O	-1.32999600	0.54953900	3.47813900
O	-0.47866900	0.00912200	1.51982000
N	-2.50429500	-0.19014100	-0.65824900
O	-0.13255600	-0.51111700	-2.73086400
H	0.49618000	-1.22007600	-2.95239400
H	0.22212600	0.28668800	-3.16104200
O	5.69164800	-0.35905900	0.36394000
C	6.55120700	0.80433000	0.37679500
C	6.52638900	1.51970400	1.72018100
H	7.54263500	0.39416000	0.17474700
H	6.28799400	1.46693700	-0.45421000
H	6.80729100	0.83160300	2.52355400
H	7.24661300	2.34476900	1.70409900
H	5.53954600	1.93440000	1.94830800

$Ru^{III}(H_2O)(EtOtpy)(QC)$

N	1.64854600	-0.26618200	-0.27659400
N	0.13404400	-2.40325900	-0.03857400
N	-0.01276800	1.72230200	-0.83289700
C	2.32863500	-1.39275300	0.04489000
C	3.69748100	-1.33585600	0.23954900
C	4.35853800	-0.09608000	0.10601200
C	3.61543800	1.05969200	-0.21490000
C	2.24406000	0.93869400	-0.40011300
C	1.47530900	-2.59771500	0.16239400
C	1.97384100	-3.86524100	0.45587000
C	1.09496200	-4.94410600	0.54880600
C	-0.26625900	-4.73216100	0.34760800

C	-0.70724700	-3.44346100	0.05641800
C	1.31416300	2.05177300	-0.73023300
C	1.73532100	3.36240800	-0.93636600
C	0.79651400	4.34698900	-1.25072900
C	-0.54576600	3.99863500	-1.35818600
C	-0.90926800	2.67085100	-1.14267100
H	4.28222200	-2.20983000	0.49573900
H	4.09702700	2.02187800	-0.30713400
H	3.03588100	-4.01181000	0.61007400
H	1.47405700	-5.93440200	0.77702200
H	-0.98426000	-5.54117800	0.41428900
H	-1.75720100	-3.22786700	-0.10144700
H	2.78355500	3.62189900	-0.85742800
H	1.11866600	5.37018200	-1.41142800
H	-1.30685500	4.72962400	-1.60447600
H	-1.94118300	2.34975300	-1.21880200
Ru	-0.35743400	-0.36886700	-0.43899400
C	-4.44204100	-0.99568100	-1.89444400
C	-5.27516700	-0.33625100	-1.02324300
C	-4.73624200	0.29061100	0.12538300
C	-3.32193300	0.21914600	0.36486400
C	-3.06551700	-0.99865300	-1.62594000
H	-6.64510700	1.02544200	0.81518500
H	-4.81222000	-1.49409900	-2.78246000
H	-6.34540500	-0.28538600	-1.19926200
C	-5.58093200	0.98724000	1.02758400
C	-2.80230200	0.82500600	1.55516500
H	-2.38048800	-1.47626800	-2.31606100
C	-3.67709500	1.50283500	2.39605500

C	-5.05701700	1.59907300	2.14124600
H	-3.25952600	1.95305900	3.28912200
H	-5.69691100	2.13834100	2.83156900
C	-1.38845600	0.74058300	2.06330200
O	-1.02635400	1.38001100	3.04017200
O	-0.55255200	-0.13240100	1.49965100
N	-2.51435600	-0.41079900	-0.56107800
O	-0.19758100	-0.78089400	-2.56266900
H	0.53597100	-1.34885300	-2.86086600
H	-0.21393900	-0.01103700	-3.16069100
O	5.67553400	-0.12951200	0.29901500
C	6.48598500	1.07487000	0.21907000
C	6.44681300	1.86840800	1.51577200
H	7.48809000	0.69025700	0.02430600
H	6.17595600	1.66455300	-0.64871900
H	6.76656400	1.24415500	2.35552700
H	7.13310600	2.71792600	1.43497800
H	5.44752400	2.25863100	1.73235500

Ru^{III}(OH)(EtOtpy)(QC)

N	1.65879300	-0.21213500	-0.31511900
N	-0.18231600	-2.11503500	-0.50044700
N	0.34862800	2.05460300	-0.43173300
C	2.14369900	-1.47310700	-0.23940500
C	3.50022600	-1.67773200	-0.04747900
C	4.35647500	-0.56210800	0.07184100
C	3.81775000	0.73429200	0.00984400
C	2.44384700	0.87396100	-0.18643700
C	1.11184900	-2.53644200	-0.36340700

C	1.41519000	-3.89698600	-0.34091900
C	0.38745100	-4.83286600	-0.46000100
C	-0.92494400	-4.38994000	-0.59985800
C	-1.16565200	-3.01760300	-0.61533400
C	1.70351100	2.15642300	-0.25998900
C	2.31360100	3.40666400	-0.15923100
C	1.53429600	4.56039000	-0.23122000
C	0.15710400	4.44200300	-0.40346000
C	-0.39627200	3.16781100	-0.49967000
H	3.93335400	-2.66770600	0.01678700
H	4.44505100	1.60718000	0.11697000
H	2.44064400	-4.22854900	-0.23327400
H	0.61595300	-5.89332300	-0.44359200
H	-1.75254800	-5.08324400	-0.69565800
H	-2.16772200	-2.61896200	-0.72246500
H	3.38561400	3.48452100	-0.02600400
H	2.00141400	5.53640400	-0.15338100
H	-0.48517400	5.31295700	-0.46411000
H	-1.46123000	3.02081000	-0.63549800
Ru	-0.32494300	0.03928500	-0.54945300
C	-4.27730800	0.66548200	-2.30213800
C	-5.20150600	0.62689100	-1.28893800
C	-4.77361900	0.41076000	0.04316300
C	-3.36953400	0.24141300	0.31903600
C	-2.92391100	0.48662200	-1.97464800
H	-6.77843000	0.49181800	0.83920300
H	-4.55976300	0.82523900	-3.33652400
H	-6.26180700	0.75596400	-1.48646600
C	-5.72985300	0.35704700	1.08862200

C	-2.96899100	0.01779800	1.68142500
H	-2.15930900	0.50011500	-2.74352900
C	-3.95851900	-0.03432400	2.65611600
C	-5.32786400	0.13407800	2.38208700
H	-3.63101100	-0.21185600	3.67328900
H	-6.05078500	0.08765700	3.19036600
C	-1.55927400	-0.15714700	2.23580300
O	-1.43032100	-0.47200100	3.42198000
O	-0.51603700	0.07524100	1.49052300
N	-2.47483200	0.28518700	-0.73457700
O	-0.14633800	0.02800000	-2.51807600
H	0.74831700	0.30557500	-2.78422800
O	5.65239900	-0.85479400	0.23614700
C	6.64117300	0.19260400	0.40064700
C	6.70055600	0.69791600	1.83454300
H	7.57673100	-0.29410600	0.11968000
H	6.44959100	0.99353800	-0.32028400
H	6.90332600	-0.12880900	2.52221300
H	7.51038400	1.42966300	1.92489400
H	5.76845300	1.18329000	2.14011000

Ru^{III}(OOH)(EtOtpy)(QC)

N	1.63951600	0.22308100	-0.17989400
N	0.04759100	-1.81021600	-0.77581900
N	0.06053600	2.30867400	0.07186900
C	2.27250300	-0.95758300	-0.31118500
C	3.64808900	-1.04231800	-0.10869000
C	4.35139300	0.12730800	0.23607200
C	3.65123800	1.34127400	0.38290300

C	2.28130400	1.35951400	0.16932000
C	1.38128500	-2.09600600	-0.66482100
C	1.84891500	-3.39188000	-0.87764300
C	0.94695600	-4.40480500	-1.20583900
C	-0.40634700	-4.10040900	-1.31656800
C	-0.81309600	-2.78598100	-1.09343200
C	1.39089500	2.53656000	0.29871300
C	1.84724700	3.80970600	0.63817500
C	0.93684700	4.85992700	0.74834800
C	-0.41483400	4.61382000	0.52001700
C	-0.81095500	3.32122800	0.18487800
H	4.15737600	-1.98966000	-0.20761700
H	4.20518200	2.22848000	0.66217200
H	2.90506400	-3.61608200	-0.79298400
H	1.30393300	-5.41561400	-1.37261800
H	-1.14130500	-4.85559400	-1.57030500
H	-1.85480600	-2.49683300	-1.17011700
H	2.90199200	3.98254800	0.81498200
H	1.28318800	5.85400200	1.01073600
H	-1.15716200	5.39992300	0.59746600
H	-1.85057400	3.07791100	-0.00093300
Ru	-0.35682900	0.28769000	-0.43332300
C	-4.40931800	0.80106400	-2.02546600
C	-5.28116400	0.23529300	-1.12967900
C	-4.78781000	-0.31041000	0.07962200
C	-3.37590200	-0.25236600	0.36090300
C	-3.04093300	0.79370800	-1.71368700
H	-6.74179400	-0.94026500	0.74631300
H	-4.74122800	1.23312200	-2.96255500

H	-6.34808400	0.19448800	-1.32901500
C	-5.68557000	-0.91144500	0.99803400
C	-2.91315900	-0.77862600	1.61600400
H	-2.31916500	1.19349700	-2.41609400
C	-3.84454700	-1.36188300	2.46641800
C	-5.21803000	-1.44419400	2.17364400
H	-3.46840300	-1.75245000	3.40426200
H	-5.89481400	-1.91229600	2.88136400
C	-1.50382800	-0.72361800	2.19554300
O	-1.29752200	-1.25080500	3.29234600
O	-0.56175200	-0.07556400	1.57374600
N	-2.52908400	0.29123700	-0.58862200
O	5.67207100	0.19969300	0.44356100
C	6.51177000	-0.97809300	0.34651100
C	6.48385400	-1.80420100	1.62398100
H	6.22731000	-1.55905800	-0.53642300
H	7.50726500	-0.56699900	0.17012000
H	5.49239900	-2.22219900	1.82420300
H	7.19062100	-2.63555400	1.53005600
H	6.78211600	-1.19212300	2.48065800
O	-0.20987600	0.61909900	-2.35466300
O	0.86262000	1.50439900	-2.73519800
H	0.72632300	1.53489400	-3.70099400

Ru^{IV}(O)(EtOtpy)(QC)

N	1.66864500	0.25678600	-0.35163000
N	0.23790700	-1.93928700	-0.69670200
N	-0.07457100	2.24948800	-0.35575800
C	2.38576000	-0.88186700	-0.31878800

C	3.75647600	-0.83671100	-0.07789600
C	4.36128400	0.41734500	0.13156900
C	3.56904500	1.58513900	0.11549400
C	2.20974400	1.47485300	-0.12641400
C	1.58503400	-2.11509700	-0.53527900
C	2.13982400	-3.39327000	-0.56945700
C	1.31077100	-4.49810500	-0.76697200
C	-0.05781800	-4.30293500	-0.92707000
C	-0.55484100	-3.00144700	-0.88590300
C	1.23290100	2.59301500	-0.14894800
C	1.59386000	3.92640100	0.03683800
C	0.60945800	4.91455700	0.01127600
C	-0.71737700	4.54910600	-0.19808600
C	-1.01734500	3.20019700	-0.37784900
H	4.33517400	-1.74800800	-0.04451100
H	4.04958100	2.53770200	0.29819600
H	3.20650600	-3.53320800	-0.44525400
H	1.73571200	-5.49576900	-0.79473100
H	-0.73709700	-5.13298600	-1.08276600
H	-1.61133300	-2.79360100	-1.00753000
H	2.63043400	4.19549900	0.19854900
H	0.88200300	5.95492600	0.15348700
H	-1.51295900	5.28466100	-0.22476000
H	-2.03305300	2.86208900	-0.54591300
Ru	-0.32778300	0.13363400	-0.63866000
O	-0.20190100	0.28590900	-2.43437200
C	-4.40116400	0.04971200	-2.23987700
C	-5.27024500	-0.18104200	-1.20416400
C	-4.77063900	-0.33388600	0.11080000

C	-3.35144500	-0.24197100	0.35280000
C	-3.03143600	0.12075100	-1.94940800
H	-6.73696200	-0.63959800	0.94550400
H	-4.73673700	0.17306100	-3.26310200
H	-6.34113800	-0.25089000	-1.37119500
C	-5.67688600	-0.57786200	1.17373700
C	-2.88931000	-0.39609500	1.70584100
H	-2.31262400	0.29182000	-2.74196800
C	-3.83144100	-0.63722000	2.69861700
C	-5.21319300	-0.73178800	2.45586300
H	-3.45164400	-0.75062500	3.70672700
H	-5.89626100	-0.92088800	3.27782500
C	-1.45973800	-0.31375400	2.23234000
O	-1.26636100	-0.49727500	3.43844500
O	-0.48530800	-0.03833700	1.42376000
N	-2.51154400	-0.01550800	-0.72646200
O	5.66290200	0.61665600	0.35418100
C	6.59577600	-0.49341300	0.41971700
C	6.60156200	-1.14914900	1.79233000
H	6.37811300	-1.20133200	-0.38575800
H	7.55890900	-0.02733800	0.20559400
H	5.64180500	-1.61734000	2.03198500
H	7.37343600	-1.92572500	1.81491700
H	6.83115300	-0.41106300	2.56684000

Ru^{IV}(OH)(EtOtpy)(QC)

N	1.66447500	0.27194600	-0.29442700
N	0.06372900	-1.75805400	-0.80681300
N	0.07332700	2.36746500	-0.02522400

C	2.29564500	-0.92159400	-0.38248100
C	3.66735200	-0.99496100	-0.19223200
C	4.37373100	0.19493600	0.09236400
C	3.67186100	1.41945800	0.19668300
C	2.30521000	1.43195500	0.00289800
C	1.39624900	-2.05992400	-0.68899900
C	1.83854200	-3.36674600	-0.86921300
C	0.91546900	-4.36911200	-1.17347600
C	-0.43168900	-4.04374500	-1.29880000
C	-0.82048500	-2.71977400	-1.10924200
C	1.41330000	2.60863700	0.12161100
C	1.87199400	3.89607700	0.38548700
C	0.95335700	4.93982600	0.50691100
C	-0.40588100	4.67452800	0.36661300
C	-0.80871900	3.36798900	0.09976400
H	4.17710200	-1.94500100	-0.25484800
H	4.22969200	2.31530400	0.43665800
H	2.89027500	-3.60749500	-0.77914200
H	1.25339600	-5.38990100	-1.31479000
H	-1.17848800	-4.79035400	-1.54120000
H	-1.85516500	-2.41389000	-1.20320800
H	2.93226500	4.08665500	0.49680200
H	1.30220800	5.94606000	0.71200400
H	-1.15134300	5.45540100	0.45938600
H	-1.85381200	3.10859300	-0.01675800
Ru	-0.32940600	0.31660100	-0.48445400
O	-0.24827900	0.67480500	-2.37461800
C	-4.39973100	0.97445000	-1.97606400
C	-5.26426800	0.38461800	-1.08596300

C	-4.75769800	-0.23076500	0.08288400
C	-3.34254800	-0.22017300	0.32701600
C	-3.02594900	0.92518000	-1.70274100
H	-6.70339900	-0.84713700	0.78198900
H	-4.74384900	1.45779400	-2.88255600
H	-6.33536600	0.37815200	-1.26318400
C	-5.63989400	-0.85791500	1.00028000
C	-2.86449300	-0.82888400	1.53337400
H	-2.31496700	1.34233200	-2.40528300
C	-3.77403400	-1.43770200	2.39112100
C	-5.15549100	-1.46517200	2.13454200
H	-3.38182800	-1.89155600	3.29397500
H	-5.82604700	-1.95059100	2.83528300
C	-1.45010800	-0.83212500	2.02269700
O	-1.07938700	-1.51413300	2.96175600
O	-0.60212300	0.02452800	1.43865900
N	-2.50616100	0.35266500	-0.61327000
H	0.56019700	0.37422300	-2.83379500
O	5.68320200	0.27793300	0.28044100
C	6.54306200	-0.89757800	0.23154900
C	6.55085700	-1.63862500	1.55872600
H	6.24327500	-1.52973900	-0.60886700
H	7.52436300	-0.47800400	0.00724000
H	5.57049300	-2.05864100	1.80414800
H	7.26900000	-2.46313900	1.49999500
H	6.85711600	-0.96986500	2.36850300

Ru^{V}(O)(EtOtpy)(QC)

N	1.66846100	0.26873500	-0.31271600

N	0.02778300	-1.74083100	-0.81407700
N	0.13377100	2.40011400	-0.09045800
C	2.27452900	-0.94312000	-0.40791600
C	3.64083800	-1.04364500	-0.21466100
C	4.37316500	0.13064700	0.08154600
C	3.69724200	1.37049100	0.19754700
C	2.33448600	1.41589700	0.00096200
C	1.35537100	-2.06460700	-0.71231700
C	1.77395800	-3.37828200	-0.89523100
C	0.82944500	-4.36590800	-1.18441700
C	-0.51307300	-4.01834800	-1.29032100
C	-0.87743600	-2.68672100	-1.09957100
C	1.46973600	2.60985500	0.11388600
C	1.94318600	3.88068300	0.42748600
C	1.03990000	4.93942500	0.53547500
C	-0.31692300	4.70456500	0.33395700
C	-0.73502200	3.41211400	0.02169600
H	4.13162000	-2.00327600	-0.28103900
H	4.27458100	2.25236300	0.44257200
H	2.82242900	-3.63719400	-0.81919500
H	1.14836100	-5.39252000	-1.32730200
H	-1.27628200	-4.75284300	-1.51801200
H	-1.90819500	-2.36472500	-1.17986800
H	3.00124000	4.04668700	0.58849400
H	1.39901000	5.93349900	0.77829000
H	-1.05051600	5.49799800	0.41356800
H	-1.77930300	3.17647200	-0.14225100
Ru	-0.30282000	0.36067400	-0.56606200
O	-0.40857100	0.69774500	-2.27023100

C	-4.49412500	0.97764800	-1.90027800
C	-5.31800000	0.33936500	-1.00571900
C	-4.76205100	-0.28158900	0.13691800
C	-3.34321700	-0.22076200	0.35118800
C	-3.11283700	0.95912800	-1.66398400
H	-6.66542600	-0.99533100	0.85955800
H	-4.87346500	1.46969900	-2.78783100
H	-6.39180900	0.29877400	-1.16033300
C	-5.59854400	-0.96465400	1.05861200
C	-2.82449600	-0.81893700	1.54484900
H	-2.44112300	1.41210900	-2.38286100
C	-3.68584300	-1.48378600	2.40808300
C	-5.06875900	-1.57347600	2.17124100
H	-3.25703900	-1.92480100	3.30091400
H	-5.70541200	-2.10139000	2.87285100
C	-1.41163000	-0.72061400	2.00716600
O	-0.96927800	-1.34068700	2.95968000
O	-0.64667500	0.16974400	1.37442700
N	-2.54300300	0.38502200	-0.60109000
O	5.67919000	0.18258200	0.27115700
C	6.52205100	-1.00746100	0.20057500
C	6.52713100	-1.76116100	1.52003000
H	6.20367400	-1.62346500	-0.64453800
H	7.50662200	-0.59820500	-0.02723400
H	5.54261500	-2.17057800	1.76652500
H	7.23371300	-2.59439900	1.44714000
H	6.84788000	-1.10533200	2.33459800

$Ru^{II}(H_2O)(Tpy)(pic)_2$

N	0.02340100	-0.02901300	1.41356500
N	0.20669000	-2.09922200	-0.19659600
N	-0.23582900	2.07566800	-0.14010100
C	0.22339900	-1.20962000	2.04881100
C	0.27642600	-1.24249200	3.44430200
C	0.11518000	-0.05309600	4.15472700
C	-0.09470500	1.14841600	3.47801500
C	-0.13659800	1.14074300	2.08198200
C	0.33225200	-2.37301400	1.14049100
C	0.51466600	-3.68042400	1.59437500
C	0.56072300	-4.72837200	0.67673600
C	0.41311400	-4.44520600	-0.67886600
C	0.23834200	-3.11953800	-1.06928800
C	-0.31758400	2.31996600	1.20570700
C	-0.53620500	3.61144000	1.68730800
C	-0.66778400	4.66805500	0.78713800
C	-0.57277800	4.41069400	-0.57862700
C	-0.35738300	3.10009300	-0.99893100
H	0.43762800	-2.17427100	3.97222900
H	-0.21714400	2.07099700	4.03193500
H	0.61676200	-3.88208000	2.65381100
H	0.70350500	-5.74749400	1.02001000
H	0.43224900	-5.22748500	-1.42912200
H	0.11801200	-2.85895000	-2.11469100
H	-0.60527600	3.79386600	2.75292800
H	-0.83941300	5.67515100	1.15204400
H	-0.66391100	5.20239100	-1.31358700
H	-0.27646100	2.84822800	-2.05065600
C	2.86141500	-0.66203700	-1.46670000

C	2.85655700	1.09522000	0.02341500
C	4.24022400	-0.59025800	-1.60872200
H	2.29459800	-1.41329600	-2.00357400
C	4.23645200	1.22176000	-0.06357700
H	2.28813400	1.75541200	0.66556000
H	4.73811200	-1.29208300	-2.27012000
H	4.73192100	1.98746700	0.52477600
C	-2.84180700	0.55246400	-1.58064100
C	-2.86858800	-0.94978700	0.16772000
C	-4.22268000	0.48857300	-1.70680600
H	-2.25488800	1.18280600	-2.23725800
C	-4.25088100	-1.06176600	0.10106700
H	-2.31108600	-1.51023400	0.90729800
H	-4.70951600	1.08734800	-2.47004900
H	-4.75966200	-1.71685900	0.80108900
Ru	-0.00061800	0.00630200	-0.56904400
N	-2.15480100	-0.15697300	-0.65881000
N	2.15735500	0.16947800	-0.66699600
C	4.97083500	0.37226200	-0.90018500
C	-4.97032900	-0.33601600	-0.85642200
C	6.46304500	0.50036800	-1.04705900
H	6.92602100	-0.46614300	-1.26690900
H	6.70491000	1.17718400	-1.87671500
H	6.91856400	0.91319900	-0.14231200
C	-6.46568000	-0.45375200	-0.98056000
H	-6.90706400	0.46866000	-1.36925100
H	-6.72571600	-1.26190100	-1.67641200
H	-6.92832400	-0.68850300	-0.01743600
O	-0.03072000	0.23464400	-2.81167700

H	0.15339100	-0.06252600	5.23845200
H	0.84003200	0.41233500	-3.20818000
H	-0.40792100	-0.49529800	-3.33241700

$Ru^{III}(H_2O)(Tpy)(pic)_2$

N	-0.13723600	-0.13908500	1.38385800
N	0.37495600	-2.08505200	-0.26800800
N	-0.11752300	2.03757100	-0.05076000
C	0.00256100	-1.34510400	1.97918500
C	-0.14847000	-1.45611200	3.36298800
C	-0.44035300	-0.31289600	4.10326200
C	-0.55855100	0.92333100	3.46941100
C	-0.39549100	0.99104400	2.08590300
C	0.30176200	-2.44426800	1.04891600
C	0.49433800	-3.76671500	1.44148600
C	0.75801000	-4.73590800	0.47433700
C	0.82324600	-4.35997300	-0.86424600
C	0.63266600	-3.02162900	-1.19740400
C	-0.41159300	2.21807600	1.27309400
C	-0.64476200	3.49371700	1.77863700
C	-0.56452800	4.59252600	0.92247000
C	-0.24455300	4.39421400	-0.41872300
C	-0.02465200	3.09714000	-0.87268800
H	-0.03895500	-2.41415600	3.85478700
H	-0.76140400	1.81716800	4.04523500
H	0.43908800	-4.04080500	2.48738500
H	0.90840800	-5.76891500	0.76811000
H	1.01883500	-5.07981600	-1.65007800
H	0.67593900	-2.69816200	-2.22953800

H	-0.88283000	3.63230600	2.82588500
H	-0.74407200	5.59078800	1.30609600
H	-0.16524200	5.22178000	-1.11347800
H	0.21214100	2.88887400	-1.90838100
C	2.91901600	-0.29924200	-1.63266500
C	2.81248900	0.83643400	0.37740800
C	4.29983600	-0.18348100	-1.66688500
H	2.40338700	-0.82256800	-2.42871900
C	4.19084700	0.98308000	0.40812500
H	2.20834500	1.23188900	1.18212600
H	4.83956100	-0.61468600	-2.50325000
H	4.64241500	1.49612900	1.25057300
C	-2.83247300	0.77849600	-1.44341500
C	-2.83022600	-1.14230800	-0.14638800
C	-4.21244200	0.74328600	-1.55614400
H	-2.26169600	1.56493700	-1.92037800
C	-4.21071700	-1.23784200	-0.22616100
H	-2.26245000	-1.88072700	0.40291100
H	-4.71040900	1.52135600	-2.12488900
H	-4.70737000	-2.06075400	0.27656600
Ru	0.00407600	0.00956600	-0.63649300
N	-2.13664200	-0.14866700	-0.74398800
N	2.16735300	0.20636200	-0.62875900
C	4.97851300	0.47324300	-0.63173600
C	-4.94451300	-0.28308300	-0.94271800
C	6.47220600	0.63846500	-0.64625500
H	6.95693000	-0.17177700	-1.19776900
H	6.73846300	1.58142300	-1.14098800
H	6.87838100	0.67289600	0.36852000

C	-6.43956000	-0.36621300	-1.07015200
H	-6.89019300	0.62996600	-1.10396300
H	-6.70635700	-0.87889800	-2.00332600
H	-6.88029700	-0.92994000	-0.24373200
O	-0.05852600	0.55422900	-2.76210700
H	-0.56377000	-0.38251400	5.17801700
H	0.78084500	0.71060200	-3.23302700
H	-0.59083600	-0.01873700	-3.34638600

$Ru^{III}(OH)(Tpy)(pic)_2$

N	0.07608700	0.03823200	1.44065300
N	0.16658500	-2.06574900	-0.10003300
N	-0.25311800	2.05980000	-0.17657900
C	0.30523800	-1.11575400	2.10492000
C	0.45155600	-1.10048000	3.49350100
C	0.35140700	0.11866600	4.16419700
C	0.10872200	1.29637200	3.45751600
C	-0.02574300	1.22943200	2.06876300
C	0.33643300	-2.30897000	1.23386500
C	0.48436000	-3.61292500	1.70449300
C	0.44808200	-4.67613300	0.80336900
C	0.25398100	-4.41417000	-0.55096700
C	0.11556500	-3.09214600	-0.96523900
C	-0.26434000	2.36691800	1.15560100
C	-0.47990700	3.67722300	1.57984600
C	-0.68597400	4.67961400	0.63251100
C	-0.67164700	4.35086300	-0.72087700
C	-0.45384300	3.02535000	-1.08789700
H	0.63803300	-2.01414500	4.04400600

H	0.03322200	2.24200200	3.97977700
H	0.61901900	-3.79839200	2.76307700
H	0.56246800	-5.69408000	1.15986700
H	0.20836100	-5.21107400	-1.28406600
H	-0.03933800	-2.83226700	-2.00602200
H	-0.49145900	3.91363400	2.63674700
H	-0.85531800	5.70202500	0.95262100
H	-0.82600400	5.09927100	-1.48939600
H	-0.43219600	2.71311000	-2.12512400
C	2.83536000	-0.77227200	-1.46647600
C	2.85154700	1.12984100	-0.15989000
C	4.21417500	-0.73031400	-1.61357300
H	2.25912200	-1.56413700	-1.92914100
C	4.23176300	1.23196100	-0.26216500
H	2.29177000	1.85898400	0.41037000
H	4.70319500	-1.50053700	-2.20101400
H	4.73576900	2.04954400	0.24276600
C	-2.83543500	0.16838600	-1.79949000
C	-2.89131100	-0.62230400	0.37060900
C	-4.21798600	0.10003200	-1.89606900
H	-2.22386000	0.49624600	-2.63055200
C	-4.27611900	-0.71787200	0.34122700
H	-2.34406900	-0.90005100	1.26213200
H	-4.69473200	0.40324900	-2.82288200
H	-4.79700900	-1.07568600	1.22337300
Ru	-0.00886300	-0.00861000	-0.63019400
N	-2.16762400	-0.18517300	-0.67990200
N	2.14534600	0.14290500	-0.75025100
C	4.95531200	0.29165600	-1.00626700

C	-4.98130000	-0.35229600	-0.81156000
C	6.44831500	0.38570300	-1.16445600
H	6.90191300	-0.60606800	-1.25061700
H	6.69581400	0.94074800	-2.07846000
H	6.90604400	0.91345600	-0.32312600
C	-6.48018300	-0.45825600	-0.89257900
H	-6.90695800	0.38693000	-1.44170300
H	-6.76697500	-1.37244600	-1.42777900
H	-6.93287100	-0.49785900	0.10190300
O	-0.08230000	0.00240800	-2.59063800
H	0.46288400	0.15079300	5.24233600
H	0.80243600	0.15747100	-2.97014800

$Ru^{III}(OOH)(Tpy)(pic)_2$

N	0.05941200	0.18210100	1.49121200
N	-0.06403000	2.10188800	-0.29121500
N	0.07879600	-2.05394000	0.10110800
C	0.03874600	1.42530900	2.02222900
C	0.07136900	1.58255900	3.40965400
C	0.11858300	0.44094800	4.21048600
C	0.13561300	-0.83019200	3.63557800
C	0.10804500	-0.93783400	2.24321900
C	-0.02301700	2.51051200	1.01470000
C	-0.05355900	3.86695000	1.33534000
C	-0.13040100	4.81136200	0.31147800
C	-0.18128700	4.37952800	-1.01131800
C	-0.14628200	3.01140500	-1.27371300
C	0.12876300	-2.19461900	1.46060400
C	0.20281200	-3.45886300	2.04318600

C	0.22945000	-4.59132400	1.22945200
C	0.18301700	-4.43479100	-0.15283500
C	0.10757800	-3.14628700	-0.67739000
H	0.05753000	2.56670200	3.86095300
H	0.16971400	-1.71151600	4.26351900
H	-0.01983700	4.18611700	2.36982600
H	-0.15306900	5.86912800	0.55059400
H	-0.24600500	5.08077100	-1.83520200
H	-0.19542500	2.61646500	-2.28224600
H	0.24185900	-3.56279300	3.12043500
H	0.28662000	-5.57868700	1.67475400
H	0.20200300	-5.28599700	-0.82336300
H	0.06742100	-2.97454400	-1.74716600
C	-2.75581800	-1.01793900	-1.35476800
C	-2.94455700	0.65674300	0.22101800
C	-4.13302400	-1.17146300	-1.41188900
H	-2.10756600	-1.62037200	-1.97754300
C	-4.32920600	0.55974900	0.21336600
H	-2.45453600	1.37855600	0.86069800
H	-4.55146000	-1.91337800	-2.08429500
H	-4.90497100	1.21781700	0.85598600
C	2.78088800	-0.81833200	-1.54842300
C	2.96651000	0.70745800	0.17217200
C	4.15992400	-0.90689800	-1.66937200
H	2.13134800	-1.38596300	-2.20303100
C	4.35271800	0.67008900	0.10633000
H	2.47484200	1.34198600	0.89770200
H	4.58021500	-1.56896700	-2.41950300
H	4.92755000	1.28410500	0.79194300

Ru	0.00563200	-0.00972900	-0.54846800
N	2.17620200	-0.02471300	-0.63870300
N	-2.15318500	-0.11697600	-0.54947600
C	-4.96425600	-0.37465200	-0.61371800
C	4.99042900	-0.15091800	-0.83153100
C	-6.46057600	-0.53154300	-0.62850500
H	-6.81745200	-0.84914000	-1.61265800
H	-6.76680600	-1.29780500	0.09529600
H	-6.96206600	0.40075500	-0.35369200
C	6.48937400	-0.20079200	-0.95186800
H	6.82960200	-1.19016600	-1.27167000
H	6.83185700	0.52287800	-1.70273300
H	6.97444800	0.05161200	-0.00473700
O	0.09939700	-0.18596000	-2.50181200
H	0.14132800	0.54258700	5.28987400
O	-0.93475200	0.46499800	-3.22880400
H	-0.63350400	0.32009100	-4.14757500

$Ru^{IV}(O)(Tpy)(pic)_2$

N	-0.00002200	0.00000700	1.42009000
N	-0.25169200	2.07154900	-0.19113700
N	0.25163200	-2.07152400	-0.19115500
C	-0.19231000	1.17184900	2.05734900
C	-0.19966400	1.19863800	3.45399600
C	0.00038400	0.00005500	4.14186000
C	0.20022400	-1.19855200	3.45398000
C	0.19244600	-1.17181400	2.05733500
C	-0.34957200	2.33510900	1.14866800
C	-0.56001900	3.63699800	1.59888500

C	-0.66724800	4.67657900	0.67451500
C	-0.55566900	4.39347100	-0.68351600
C	-0.34786900	3.07343300	-1.07791400
C	0.34950300	-2.33509700	1.14864900
C	0.55973400	-3.63702300	1.59886400
C	0.66679000	-4.67662000	0.67449400
C	0.55525300	-4.39349300	-0.68353700
C	0.34764400	-3.07342400	-1.07793300
H	-0.35225500	2.12122100	3.99969400
H	0.35302100	-2.12111000	3.99966300
H	-0.63872100	3.84199400	2.65947300
H	-0.83184100	5.69226700	1.01754900
H	-0.62642800	5.17097300	-1.43515900
H	-0.25176100	2.79859500	-2.12168900
H	0.63837400	-3.84203700	2.65945300
H	0.83121200	-5.69233600	1.01752600
H	0.62588500	-5.17100500	-1.43518100
H	0.25156600	-2.79857200	-2.12170700
C	-2.84602100	0.34723600	-1.74541700
C	-2.86922800	-0.84312400	0.23594900
C	-4.22780000	0.27927300	-1.84362800
H	-2.25456300	0.83998600	-2.50688000
C	-4.25215400	-0.95017500	0.19839100
H	-2.31128500	-1.28122200	1.05263300
H	-4.71640400	0.74472900	-2.69338200
H	-4.75866900	-1.47770400	0.99992100
C	2.84612200	-0.34751900	-1.74504400
C	2.86920800	0.84339100	0.23598800
C	4.22790800	-0.27961300	-1.84317500

H	2.25471100	-0.84047100	-2.50641300
C	4.25214100	0.95040400	0.19849900
H	2.31122500	1.28174200	1.05250600
H	4.71656300	-0.74531800	-2.69276500
H	4.75860700	1.47815000	0.99991800
Ru	0.00000500	0.00002100	-0.68264600
N	2.16326500	0.20172900	-0.71731700
N	-2.16323100	-0.20171600	-0.71749100
C	-4.97368700	-0.38220500	-0.85914400
C	4.97373900	0.38212500	-0.85882100
C	-6.47078100	-0.49975300	-0.94651100
H	-6.90369900	0.33262800	-1.50839700
H	-6.74557800	-1.42745600	-1.46477500
H	-6.92585600	-0.53204900	0.04768800
C	6.47084100	0.49962100	-0.94612100
H	6.90380700	-0.33309500	-1.50747600
H	6.74568200	1.42701100	-1.46492000
H	6.92582900	0.53250700	0.04809700
O	0.00007600	0.00008000	-2.48827800
H	0.00056100	0.00007700	5.22636700

$Ru^{IV}(OH)(Tpy)(pic)_2$

N	0.15224500	-0.02848700	1.40268800
N	0.06451700	-2.07637000	-0.21425000
N	-0.23072300	2.07027900	-0.11442700
C	0.37995600	-1.22297000	2.00733400
C	0.59994000	-1.26079500	3.38184100
C	0.57316500	-0.06194900	4.09750000
C	0.32276300	1.15188700	3.45343000

C	0.11445700	1.14805000	2.07670600
C	0.30877700	-2.38005300	1.09705900
C	0.41824900	-3.70689700	1.50295100
C	0.26020300	-4.72577100	0.56142600
C	-0.01511900	-4.39746800	-0.76282600
C	-0.10859700	-3.05348100	-1.11951800
C	-0.15588900	2.32233400	1.22751400
C	-0.32849000	3.61522100	1.71203600
C	-0.58215300	4.65477400	0.81467800
C	-0.65430300	4.37985100	-0.54749700
C	-0.47453000	3.06775900	-0.98040400
H	0.78315600	-2.19828800	3.89094700
H	0.29627400	2.07468400	4.01843000
H	0.61328100	-3.94627100	2.54085100
H	0.34302600	-5.76246400	0.86814300
H	-0.15732200	-5.15800300	-1.52123800
H	-0.31377100	-2.74240400	-2.13674000
H	-0.27112400	3.81254500	2.77508500
H	-0.71943700	5.66533400	1.18289300
H	-0.84392400	5.15737700	-1.27774900
H	-0.50825900	2.80569100	-2.03064000
C	2.85769500	-0.96811800	-1.07620700
C	2.77376400	1.30216100	-0.59533600
C	4.23608200	-0.92514000	-1.19295600
H	2.32778000	-1.89649100	-1.23236300
C	4.15017200	1.40651200	-0.69249300
H	2.17628200	2.17256000	-0.36925700
H	4.76648200	-1.83987000	-1.43437700
H	4.61147100	2.37427500	-0.52832700

C	-2.86195900	0.52356700	-1.60099000
C	-2.85790000	-0.79910000	0.31138400
C	-4.24440700	0.48985900	-1.65928800
H	-2.28413200	1.04331400	-2.35381800
C	-4.24103900	-0.86227400	0.31044200
H	-2.28740100	-1.29787000	1.08239700
H	-4.74530900	1.01470400	-2.46558200
H	-4.73777900	-1.42195900	1.09546400
Ru	-0.03098400	0.00719300	-0.64086900
N	-2.16893800	-0.11252300	-0.62838900
N	2.11877700	0.12889000	-0.77619900
C	4.92662700	0.27974500	-0.99915900
C	-4.97709600	-0.21294100	-0.69082100
C	6.41830900	0.36531500	-1.14010900
H	6.89554000	-0.59195200	-0.91410300
H	6.67708200	0.62908800	-2.17404500
H	6.83565700	1.13874800	-0.48946700
C	-6.47586700	-0.28854100	-0.74173800
H	-6.90444100	0.61386000	-1.18646900
H	-6.78245900	-1.14002100	-1.36331500
H	-6.90226500	-0.43588300	0.25407800
O	-0.08986500	0.06875000	-2.54681400
H	0.74404000	-0.07411700	5.16805400
H	0.74178600	0.21519300	-3.04182100

$Ru^{V}(O)(Tpy)(pic)_2$

N	0.00503400	0.04280000	1.46374000
N	0.00014100	2.04960700	-0.15443200
N	-0.00733400	-2.09221100	-0.00006300

C	0.01042800	1.24113000	2.08727200
C	0.01900800	1.32262600	3.47959900
C	0.02267900	0.14126500	4.21728000
C	0.01685900	-1.08904500	3.56302900
C	0.00743800	-1.11496700	2.16954400
C	0.00626300	2.37764800	1.16635800
C	0.00716100	3.71170700	1.56611700
C	0.00057600	4.71846100	0.60368900
C	-0.00648800	4.36344900	-0.74131800
C	-0.00634700	3.01758400	-1.09163900
C	-0.00136100	-2.32012200	1.34184800
C	-0.00537300	-3.61959200	1.84066300
C	-0.01528700	-4.69544100	0.95438600
C	-0.02147200	-4.44706800	-0.41471300
C	-0.01753100	-3.12922000	-0.86147100
H	0.02328300	2.28282000	3.97858100
H	0.01980200	-2.01082700	4.13022000
H	0.01214400	3.95823000	2.62007900
H	0.00090200	5.76001600	0.90475400
H	-0.01182700	5.10629200	-1.53021900
H	-0.01118400	2.70368500	-2.12594300
H	-0.00125900	-3.78989900	2.90975100
H	-0.01842800	-5.71108200	1.33392300
H	-0.02952700	-5.24852700	-1.14362600
H	-0.02215900	-2.89866000	-1.92047400
C	-2.74866100	-0.37635500	-1.93094100
C	-2.96126600	0.29200400	0.28506900
C	-4.12465000	-0.39939400	-2.08854300
H	-2.08936100	-0.62546900	-2.75260600

C	-4.34399000	0.28874000	0.18632800
H	-2.48104000	0.56143400	1.21503300
H	-4.53335000	-0.67293400	-3.05539500
H	-4.92812900	0.56637300	1.05703900
C	2.74442300	-0.40280900	-1.93091500
C	2.96439100	0.28396300	0.27822400
C	4.11994500	-0.43735800	-2.09000400
H	2.08229600	-0.66031100	-2.74776900
C	4.34697300	0.27029200	0.17793000
H	2.48783600	0.56021300	1.20798400
H	4.52566600	-0.73073400	-3.05223500
H	4.93403100	0.54468400	1.04774300
Ru	-0.00075000	-0.02660000	-0.69630200
N	2.16741400	-0.05034200	-0.75796300
N	-2.16826500	-0.03864600	-0.75526700
C	-4.96605900	-0.06488200	-1.01809200
C	4.96503000	-0.09388100	-1.02542000
C	-6.46221000	-0.10323000	-1.15045600
H	-6.77558700	0.09083000	-2.17982400
H	-6.83415800	-1.09786300	-0.87254300
H	-6.93900200	0.62421200	-0.48774000
C	6.45991700	-0.09076200	-1.17624400
H	6.78513900	-0.81058600	-1.93225100
H	6.79862400	0.90287800	-1.49690900
H	6.95575400	-0.31768800	-0.22842000
O	0.00140500	0.58218400	-2.31251200
H	0.02998000	0.17893800	5.30061000

$Ru^{II}(H_2O)(Tpy)(Bpy)$

Ru	-0.04009600	-0.00028200	-0.51452200
N	1.93013000	0.00254300	-0.08180300
N	0.37263700	2.09606300	-0.43771200
N	0.37911900	-2.09551300	-0.43795800
N	-2.14785000	-0.00294000	-0.85045000
N	-0.79354600	-0.00135600	1.42555800
C	2.56856200	1.19072100	0.01796300
C	3.94418800	1.21683000	0.26086300
C	3.94796900	-1.20540800	0.26083100
C	2.57213100	-1.18348700	0.01790600
C	1.68651700	2.36790200	-0.15864600
C	2.13913500	3.68370300	-0.05611500
C	1.24477800	4.73695500	-0.24145700
C	-0.08791300	4.45062800	-0.52715000
C	-0.48097400	3.11732300	-0.61544900
C	1.69380600	-2.36343700	-0.15874900
C	2.15035600	-3.67784100	-0.05611900
C	1.25915400	-4.73377900	-0.24152200
C	-0.07433900	-4.45146300	-0.52739600
C	-0.47139600	-3.11933000	-0.61578300
C	-2.90928500	-0.00336400	0.27904500
C	-4.30589300	-0.00442300	0.20694900
C	-4.92983000	-0.00516500	-1.03721000
C	-4.14278300	-0.00489900	-2.18841000
C	-2.75969400	-0.00379300	-2.04889000
C	-2.15336300	-0.00270100	1.54557700
C	-2.75832100	-0.00348300	2.80561100
C	-1.97100800	-0.00287300	3.95284200
C	-0.58388600	-0.00147100	3.81761600

C	-0.03737600	-0.00074000	2.53997000
H	4.47846600	2.15498600	0.34633400
H	4.48503700	-2.14195900	0.34628100
H	3.17929700	3.88758600	0.16774800
H	1.58845400	5.76291300	-0.16255500
H	-0.81870400	5.23676000	-0.67924800
H	-1.50712200	2.84550500	-0.83405200
H	3.19110400	-3.87859500	0.16787700
H	1.60590400	-5.75869400	-0.16252400
H	-0.80274800	-5.23978900	-0.67957400
H	-1.49832600	-2.85060800	-0.83453800
H	-4.90439400	-0.00465500	1.10926600
H	-6.01263700	-0.00596800	-1.10378000
H	-4.58318000	-0.00552100	-3.17894600
H	-2.10881900	-0.00359500	-2.91486600
H	-3.83730000	-0.00459700	2.89245500
H	-2.43534900	-0.00348800	4.93321800
H	0.07254400	-0.00093700	4.68018400
H	1.03467200	0.00042700	2.39101300
O	0.62921200	0.00068500	-2.64154600
H	0.35607600	0.77695700	-3.16051200
H	0.36089100	-0.77856600	-3.15856200
C	4.62743900	0.00674000	0.38576500
H	5.69539200	0.00831600	0.57444300

$Ru^{III}(H_2O)(Tpy)(Bpy)$

Ru	-0.04209500	-0.00000600	-0.54697000
N	1.96764700	0.00018700	-0.09219000
N	0.38897600	2.06591000	-0.26528000

N	0.38957900	-2.06573200	-0.26599400
N	-2.12431100	-0.00005900	-0.95020600
N	-0.86138400	-0.00069700	1.36211000
C	2.60913600	1.18520100	-0.01694000
C	3.99564800	1.21130500	0.14383900
C	3.99598400	-1.21046100	0.14355100
C	2.60949100	-1.18469200	-0.01723100
C	1.71660300	2.35512500	-0.10301000
C	2.14456800	3.67598400	-0.00619600
C	1.20650300	4.70652500	-0.07352700
C	-0.14206200	4.39548500	-0.23009500
C	-0.51621600	3.05808300	-0.31836600
C	1.71723200	-2.35478300	-0.10367000
C	2.14539100	-3.67560500	-0.00716900
C	1.20749400	-4.70626900	-0.07491600
C	-0.14110400	-4.39539500	-0.23156200
C	-0.51546200	-3.05803300	-0.31948600
C	-2.93075100	-0.00031100	0.15111800
C	-4.31862600	-0.00020800	0.01520700
C	-4.88013500	0.00012500	-1.26019800
C	-4.04535300	0.00033700	-2.37720200
C	-2.67001900	0.00023100	-2.18197400
C	-2.22306300	-0.00065400	1.43977300
C	-2.85764300	-0.00091500	2.68250300
C	-2.09332700	-0.00122900	3.84617800
C	-0.70351300	-0.00125800	3.74770600
C	-0.12168600	-0.00097500	2.48601200
H	4.52981500	2.15064300	0.21061300
H	4.53041100	-2.14966700	0.21011600

H	3.19602800	3.90076100	0.12217800
H	1.53024400	5.73888700	0.00104200
H	-0.90236100	5.16556500	-0.28382400
H	-1.55165800	2.76829500	-0.44461900
H	3.19687300	-3.90023400	0.12128200
H	1.53137900	-5.73860300	-0.00059300
H	-0.90127300	-5.16557900	-0.28563100
H	-1.55093100	-2.76836500	-0.44578900
H	-4.95743000	-0.00037800	0.88887800
H	-5.95841900	0.00024000	-1.37623900
H	-4.44379400	0.00059600	-3.38463600
H	-1.98078400	0.00036300	-3.01654900
H	-3.93817800	-0.00087100	2.74339600
H	-2.58010700	-0.00143200	4.81525500
H	-0.06882300	-0.00148200	4.62572000
H	0.95307300	-0.00094800	2.36389300
O	0.83044700	0.00163600	-2.58769300
H	0.62520200	0.78107200	-3.13671100
H	0.62772100	-0.77837600	-3.13683600
C	4.68374400	0.00051400	0.21856000
H	5.76104100	0.00067600	0.34074600

Ru^{III}(OH)(Tpy)(Bpy)

Ru	0.01130900	0.00003600	-0.56047900
N	-1.97399800	-0.00049900	-0.15459000
N	-0.38969500	-2.09073600	-0.36542000
N	-0.39092000	2.09064400	-0.36528900
N	2.10974000	0.00050000	-0.91011400
N	0.83546400	0.00010400	1.43036000

C	-2.60784100	-1.18850100	-0.04038800
C	-3.98486100	-1.21303900	0.19291500
C	-4.66737800	-0.00132600	0.30376700
C	-3.98556400	1.21082000	0.19297300
C	-2.60854200	1.18712900	-0.04033500
C	-1.71623900	-2.36317900	-0.16288600
C	-2.16240500	-3.68062800	-0.06975100
C	-1.24642900	-4.72588800	-0.18444700
C	0.09990100	-4.43380700	-0.38889000
C	0.48837200	-3.09948800	-0.47327000
C	-1.71762900	2.36232700	-0.16281200
C	-2.16457300	3.67951400	-0.06976900
C	-1.24920300	4.72530800	-0.18450500
C	0.09729700	4.43400200	-0.38889400
C	0.48655000	3.09989400	-0.47317200
C	2.90521900	0.00063500	0.19324500
C	4.29691400	0.00086100	0.06480000
C	4.86703400	0.00096400	-1.20610000
C	4.03718300	0.00084100	-2.32649000
C	2.65935800	0.00061000	-2.13627800
C	2.19688300	0.00051400	1.49109800
C	2.85194300	0.00080700	2.72522400
C	2.10555800	0.00066200	3.90079800
C	0.71387300	0.00021300	3.82443700
C	0.11919200	-0.00005200	2.56807600
H	-4.51818200	-2.15032000	0.28992400
H	-4.51947400	2.14775500	0.29006700
H	-3.21244300	-3.89250700	0.09011400
H	-1.58549300	-5.75381900	-0.11355800

H	0.84418600	-5.21582300	-0.48361800
H	1.52243600	-2.82039900	-0.63563600
H	-3.21474500	3.89077400	0.09004500
H	-1.58887900	5.75304300	-0.11369500
H	0.84113200	5.21644100	-0.48365700
H	1.52079000	2.82140900	-0.63546000
H	4.93236600	0.00094900	0.94113300
H	5.94630800	0.00113800	-1.31576200
H	4.44181800	0.00091500	-3.33204000
H	1.95620600	0.00051200	-2.96170700
H	3.93323700	0.00115700	2.77415600
H	2.60797500	0.00089800	4.86230600
H	0.09407500	0.00007100	4.71340600
H	-0.95852800	-0.00040900	2.45782500
O	-0.34160500	0.00032900	-2.48671900
H	-1.29263400	0.00035000	-2.70362000
H	-5.73673100	-0.00161800	0.48389400

Ru^{III}(OOH)(Tpy)(Bpy)

Ru	0.03251900	0.00945400	-0.48180600
O	-0.42578400	0.02653000	-2.38721900
N	-0.44733400	-2.07497300	-0.53088300
N	-1.94551100	0.03734300	-0.03217400
N	-0.30678800	2.11292300	-0.21707600
N	0.75050300	-0.21465900	1.52874600
N	2.16544800	0.00380600	-0.71322000
C	0.37794700	-3.09981300	-0.78777100
H	1.41171900	-2.84324900	-0.98677700
C	-0.05748000	-4.42336600	-0.80324000

H	0.64884500	-5.21716500	-1.01704900
C	-1.39869600	-4.68919100	-0.54174500
H	-1.77377300	-5.70697500	-0.54447200
C	-2.26269700	-3.62764400	-0.27453500
H	-3.30896300	-3.81734200	-0.06849100
C	-1.76907800	-2.32345900	-0.27388800
C	-2.61193400	-1.13659300	-0.00055700
C	-3.98219800	-1.13466500	0.27372900
H	-4.54738400	-2.05735500	0.31030800
C	-4.61677300	0.08687000	0.49854700
H	-5.67978700	0.10724100	0.71237300
C	-3.90071200	1.28472800	0.44857400
H	-4.40486400	2.22761600	0.61871500
C	-2.53323600	1.23621100	0.17259300
C	-1.61443100	2.39764500	0.07217000
C	-2.03342100	3.71418100	0.25349400
H	-3.06915100	3.93288800	0.48242200
C	-1.10850000	4.75240700	0.13615700
H	-1.42616000	5.78022600	0.27535200
C	0.21716700	4.45037800	-0.16029300
H	0.96871800	5.22480100	-0.26095100
C	0.57663800	3.11452300	-0.32918600
H	1.59573800	2.82971300	-0.56159900
C	-0.03388500	-0.32277100	2.61581100
H	-1.10269500	-0.30460800	2.44472400
C	0.48047200	-0.44712200	3.90116500
H	-0.19568700	-0.52812200	4.74422400
C	1.86375700	-0.46367000	4.06600300
H	2.30505900	-0.55759200	5.05246800

C	2.68031200	-0.35912800	2.94354900
H	3.75640400	-0.37175400	3.05759100
C	2.10376200	-0.23661800	1.67649500
C	2.88989900	-0.13101200	0.43123500
C	4.28634600	-0.16628500	0.39308600
H	4.85998100	-0.27737100	1.30432800
C	4.94331800	-0.06082000	-0.82990500
H	6.02709400	-0.08860800	-0.86865200
C	4.19035300	0.08156200	-1.99348600
H	4.65869800	0.16954300	-2.96704200
C	2.80324400	0.10991700	-1.89324100
H	2.17162300	0.23599500	-2.76359400
O	0.09686400	1.13174900	-3.10750600
H	-0.15018700	0.89121900	-4.02296600

Ru^{IV}(O)(Tpy)(Bpy)

Ru	-0.00749200	0.00010100	-0.63051500
O	0.26513400	0.00009500	-2.40975900
N	0.39082500	2.10304000	-0.46511400
N	1.97005200	-0.00030100	-0.16745500
N	0.38987300	-2.10294100	-0.46539700
N	-0.78332300	0.00005000	1.44790700
N	-2.14577500	0.00050800	-0.85304800
C	-0.47479200	3.11389600	-0.61946600
H	-1.50198400	2.83889300	-0.82628200
C	-0.08248300	4.44770900	-0.52407500
H	-0.81749800	5.23285800	-0.65712000
C	1.25350200	4.73340000	-0.25904900
H	1.59522300	5.75957700	-0.17733700

C	2.15783500	3.68302700	-0.09779100
H	3.20040400	3.89040300	0.10952200
C	1.70850200	2.36855400	-0.20601100
C	2.59366300	1.19111100	-0.04391500
C	3.96386800	1.21340000	0.22390400
H	4.49825800	2.14885200	0.32982800
C	4.63960900	-0.00094500	0.35337200
H	5.70416700	-0.00119900	0.56002600
C	3.96332800	-1.21497000	0.22367800
H	4.49732300	-2.15066800	0.32942300
C	2.59313200	-1.19202100	-0.04412900
C	1.70746200	-2.36905300	-0.20642700
C	2.15625300	-3.68374200	-0.09852700
H	3.19874700	-3.89160300	0.10866700
C	1.25147400	-4.73370200	-0.25995700
H	1.59276700	-5.76004200	-0.17850300
C	-0.08441100	-4.44739900	-0.52483400
H	-0.81975600	-5.23221800	-0.65799900
C	-0.47617200	-3.11340900	-0.61991700
H	-1.50325600	-2.83792200	-0.82662200
C	-0.02935700	-0.00024400	2.55975600
H	1.04465000	-0.00020800	2.41632200
C	-0.57912900	-0.00057300	3.83719700
H	0.07107900	-0.00079900	4.70417700
C	-1.96673100	-0.00060400	3.96139600
H	-2.43630700	-0.00088100	4.93933500
C	-2.75267300	-0.00028300	2.81159100
H	-3.83132700	-0.00031400	2.89819100
C	-2.13807300	0.00007000	1.55676400

C	-2.89215000	0.00046500	0.28388400
C	-4.28794600	0.00081200	0.21429500
H	-4.88421500	0.00078900	1.11753800
C	-4.91547300	0.00120400	-1.02879200
H	-5.99856500	0.00147000	-1.08948300
C	-4.13642200	0.00123400	-2.18427600
H	-4.58241100	0.00152400	-3.17204800
C	-2.75241000	0.00088100	-2.05391600
H	-2.09271400	0.00087300	-2.91369400

Ru^{IV}(OH)(Tpy)(Bpy)

Ru	0.07385000	-0.00008800	-0.55708100
O	-0.22965500	0.00124000	-2.48428200
H	-1.17070300	0.00791800	-2.75547400
N	-0.42196700	-2.06496100	-0.42322300
N	-1.97010800	0.00136000	-0.15920700
N	-0.41864200	2.06567900	-0.41952900
N	0.78363800	-0.00131600	1.45069200
N	2.13018300	0.00177200	-0.84895500
C	0.47444700	-3.05279900	-0.59436800
H	1.49949100	-2.76406600	-0.78698200
C	0.09920100	-4.39050300	-0.52351700
H	0.85128800	-5.15782000	-0.66259900
C	-1.23455800	-4.70439800	-0.27521600
H	-1.55667200	-5.73803900	-0.21486600
C	-2.16106500	-3.67569600	-0.10660700
H	-3.20286200	-3.90211200	0.08255600
C	-1.73642700	-2.35280700	-0.18725500
C	-2.61248800	-1.18296900	-0.03810100

C	-3.98457800	-1.20665000	0.21380300
H	-4.51241300	-2.14648500	0.31262700
C	-4.66597000	0.00309000	0.33776700
H	-5.73225000	0.00375000	0.53313900
C	-3.98283700	1.21190100	0.21518900
H	-4.50923800	2.15239900	0.31530700
C	-2.61073400	1.18645600	-0.03685200
C	-1.73291600	2.35512700	-0.18421700
C	-2.15607300	3.67846500	-0.10283500
H	-3.19775300	3.90590000	0.08572200
C	-1.22830900	4.70622200	-0.27001100
H	-1.54919600	5.74020200	-0.20897400
C	0.10521200	4.39082500	-0.51760100
H	0.85842300	5.15730100	-0.65526200
C	0.47893300	3.05271600	-0.58907400
H	1.50392100	2.76314400	-0.78058500
C	0.01903000	-0.00161400	2.55744700
H	-1.05291500	0.00061800	2.40885500
C	0.56981300	-0.00468000	3.83405900
H	-0.08290100	-0.00493000	4.69859100
C	1.95631300	-0.00712500	3.96196600
H	2.42193000	-0.00938600	4.94136100
C	2.75132300	-0.00628400	2.81465900
H	3.82956400	-0.00774300	2.90521900
C	2.14473800	-0.00336900	1.56124300
C	2.89299000	-0.00186200	0.28937600
C	4.28045500	-0.00333100	0.19238300
H	4.89635800	-0.00601500	1.08212400
C	4.88105100	-0.00130000	-1.06929100

H	5.96270000	-0.00244100	-1.14834200
C	4.08719400	0.00227800	-2.21750300
H	4.52255800	0.00382600	-3.20934500
C	2.70833700	0.00390700	-2.07214100
H	2.02831000	0.00659000	-2.91551600

Ru^{V}(O)(Tpy)(Bpy)

Ru	-0.00278700	-0.04500200	-0.69965500
O	-0.08338300	0.19020000	-2.40639100
N	-0.11469300	-2.09282000	-0.24332100
N	-1.96566100	-0.25978300	-0.22738800
N	-0.62722700	1.97990700	-0.47015000
N	0.70116400	0.23892800	1.34977800
N	2.14382900	0.10649500	-0.87198900
C	0.92601600	-2.94625200	-0.22510300
H	1.91308700	-2.51973900	-0.34110600
C	0.73478200	-4.31573500	-0.07935000
H	1.59552900	-4.97327400	-0.07523200
C	-0.56239700	-4.80353500	0.05787200
H	-0.74207100	-5.86625300	0.17632700
C	-1.64041600	-3.91395300	0.05177900
H	-2.65321900	-4.27980600	0.16440200
C	-1.40030100	-2.55420400	-0.09332300
C	-2.44419900	-1.51497600	-0.06597900
C	-3.80992300	-1.69703600	0.13448900
H	-4.22717100	-2.68707300	0.26692700
C	-4.63616200	-0.56945000	0.16764200
H	-5.70201900	-0.69356500	0.32148800
C	-4.10790800	0.71415800	0.02397200

H	-4.75362900	1.58145200	0.07379000
C	-2.73386800	0.85525400	-0.17374000
C	-1.98034000	2.11238200	-0.30089600
C	-2.55089900	3.37931200	-0.22075700
H	-3.61945800	3.48760600	-0.08323800
C	-1.73251200	4.50663600	-0.30916400
H	-2.16972100	5.49694300	-0.24863000
C	-0.35986100	4.34657900	-0.47305000
H	0.30861100	5.19536000	-0.55237000
C	0.16409400	3.05774500	-0.55315800
H	1.22164600	2.88392900	-0.70434400
C	-0.11661300	0.31666200	2.41683700
H	-1.17859000	0.22438800	2.23405200
C	0.37124300	0.49383300	3.70464700
H	-0.32268000	0.54236600	4.53518900
C	1.74874000	0.59936600	3.88888000
H	2.16550000	0.73985800	4.88008900
C	2.59283300	0.52603000	2.78392400
H	3.66351500	0.61014400	2.91400700
C	2.04748900	0.34895100	1.51175200
C	2.84852600	0.27623700	0.27921900
C	4.24055900	0.37062900	0.24659500
H	4.80335200	0.50791000	1.16055900
C	4.90557100	0.28667800	-0.97429000
H	5.98714300	0.35785300	-1.00725100
C	4.16762600	0.11284300	-2.14314500
H	4.64346700	0.04595800	-3.11421600
C	2.78336300	0.02804800	-2.05423000
H	2.16185700	-0.10185700	-2.93152600

$Ru^{II}(H_2O)(Tpy)(QC)$

N	-2.21444900	-0.33188000	0.04394800
N	-1.11587900	2.04804200	-0.17912100
N	-0.28894400	-2.04649500	-0.47437600
C	-3.05602800	0.69280600	0.31456400
C	-4.37258500	0.42414800	0.69774100
C	-4.78567700	-0.90485100	0.80044900
C	-3.89379400	-1.94380700	0.53163600
C	-2.58675800	-1.62845800	0.15124500
C	-2.44054500	2.03272300	0.17181600
C	-3.14234600	3.22208800	0.37599700
C	-2.48774900	4.44348000	0.22616800
C	-1.13999400	4.44937800	-0.12738900
C	-0.49480800	3.23029400	-0.32017200
C	-1.50656100	-2.59277100	-0.15896000
C	-1.68952200	-3.97658000	-0.14327200
C	-0.62164400	-4.81775100	-0.45089600
C	0.61187400	-4.25432100	-0.77102900
C	0.73295200	-2.86707300	-0.77064700
H	-5.06457900	1.22758800	0.91912200
H	-4.21493700	-2.97417000	0.62409800
H	-4.19067300	3.19802900	0.64836100
H	-3.02553700	5.37252800	0.38385600
H	-0.58968400	5.37498500	-0.25380500
H	0.55282800	3.18370300	-0.59543600
H	-2.65707200	-4.39630600	0.10473500
H	-0.75552100	-5.89435700	-0.44061200
H	1.47064100	-4.86869900	-1.01721300
H	1.67258600	-2.38360600	-1.01219700

Ru	-0.30699200	0.08419700	-0.41366900
C	3.38773300	1.07135500	-2.52571700
C	4.43012800	0.86602900	-1.65758100
C	4.16064100	0.49566800	-0.31764000
C	2.79411100	0.34844800	0.11721200
C	2.08031400	0.87037500	-2.05393500
H	6.25032300	0.38488700	0.21319300
H	3.54393500	1.36260800	-3.55833000
H	5.46236900	0.98277300	-1.97526700
C	5.23423700	0.27483500	0.58138900
C	2.55020300	0.03548900	1.49912500
H	1.24003800	0.98028000	-2.72880700
C	3.64891000	-0.16665700	2.32607700
C	4.98167200	-0.06830400	1.88654100
H	3.43846500	-0.39367400	3.36408600
H	5.79524200	-0.24520700	2.58325300
C	1.21105700	-0.04183200	2.23408100
O	1.23894200	-0.17123800	3.46702400
O	0.08690800	0.04553500	1.60230900
H	-5.80386400	-1.13160300	1.09779200
N	1.77570200	0.51318600	-0.80457300
O	-0.79590100	0.14103100	-2.59694200
H	-1.57834200	0.68660400	-2.78911900
H	-1.00810600	-0.74188100	-2.94736500

Ru^{III}(H_2O)(Tpy)(QC)

N	-2.19706400	-0.44863400	0.02763800
N	-1.28202500	2.01831900	-0.06030300
N	-0.10932000	-1.97547400	-0.59226500

C	-3.11308600	0.49138000	0.33863800
C	-4.40827600	0.09134500	0.67851500
C	-4.70279700	-1.27141400	0.68766800
C	-3.72703400	-2.21935400	0.36785000
C	-2.44743200	-1.77498600	0.03504100
C	-2.60281000	1.87849900	0.27951200
C	-3.39023800	2.99785900	0.54195200
C	-2.82357200	4.26989200	0.46248400
C	-1.47972700	4.39696100	0.12167700
C	-0.74141500	3.24352100	-0.13240600
C	-1.28513900	-2.62732800	-0.32284200
C	-1.35583300	-4.01579800	-0.39429600
C	-0.21794600	-4.74626200	-0.74293500
C	0.96777200	-4.07233300	-1.01777100
C	0.97955900	-2.68179100	-0.93258700
H	-5.17021700	0.81686100	0.93332000
H	-3.96828000	-3.27447200	0.38473400
H	-4.43468300	2.88317300	0.80447900
H	-3.42867800	5.14661800	0.66641200
H	-0.99915300	5.36581700	0.05273600
H	0.30855500	3.29092700	-0.39459600
H	-2.28559700	-4.52920400	-0.18296400
H	-0.26634800	-5.82833000	-0.79971600
H	1.87254400	-4.60021100	-1.29515700
H	1.87587400	-2.11052500	-1.14235400
Ru	-0.30568000	0.15473400	-0.36734000
C	3.30011600	1.70352400	-2.30128200
C	4.35292000	1.40450000	-1.47040100
C	4.10849200	0.78425500	-0.22230300

C	2.75660800	0.48370400	0.15934700
C	2.00675500	1.35230600	-1.88916700
H	6.19604300	0.69431700	0.32001200
H	3.44069400	2.18348700	-3.26243000
H	5.37530400	1.63557100	-1.75386500
C	5.18604500	0.45745400	0.64061200
C	2.52798800	-0.10626400	1.44528200
H	1.16117100	1.54366500	-2.53909600
C	3.62482300	-0.41288700	2.24259800
C	4.94940400	-0.14707600	1.85190200
H	3.42670500	-0.85992700	3.20967800
H	5.76916100	-0.40514500	2.51384000
C	1.19721600	-0.37522400	2.09337200
O	1.12119600	-0.97241400	3.15708400
O	0.08649300	0.13153300	1.55221000
H	-5.70212300	-1.60144500	0.94951000
N	1.73285100	0.75657200	-0.72537100
O	-0.77054600	0.30047300	-2.48374400
H	-1.68098100	0.54098200	-2.73476500
H	-0.52266200	-0.45956200	-3.04186600

Ru^{III}(OH)(Tpy)(QC)

N	-2.26798400	-0.05977100	0.02471800
N	-0.77460800	2.09633400	-0.27843600
N	-0.62654500	-2.10168100	-0.31669100
C	-2.92642100	1.10372700	0.21278900
C	-4.27725500	1.07687700	0.56943500
C	-4.90420700	-0.15958600	0.72682700
C	-4.19258500	-1.34565800	0.54277400

C	-2.84332800	-1.27015800	0.18775700
C	-2.09089200	2.31371700	0.02552700
C	-2.58302700	3.61285400	0.15132900
C	-1.72178600	4.69445600	-0.03133300
C	-0.38373700	4.45736400	-0.33665200
C	0.04979500	3.13868600	-0.45171400
C	-1.92542600	-2.41510300	-0.02212200
C	-2.32790800	-3.74704500	0.07441100
C	-1.39452600	-4.76328900	-0.12833200
C	-0.07518600	-4.42887500	-0.42343400
C	0.26744600	-3.08145200	-0.50881900
H	-4.83291200	1.99277300	0.72748300
H	-4.68268600	-2.30149200	0.68040900
H	-3.62582800	3.78364100	0.38921500
H	-2.09675000	5.70786900	0.06509600
H	0.31860000	5.26940000	-0.48526500
H	1.07925100	2.89788600	-0.68945700
H	-3.35733500	-3.99351300	0.30391500
H	-1.69964900	-5.80180100	-0.05533300
H	0.68124100	-5.18774200	-0.58675200
H	1.27874200	-2.76647200	-0.73733400
Ru	-0.31163300	0.01319400	-0.44079100
C	3.43680600	0.17482800	-2.66928500
C	4.46884000	0.13943000	-1.76631400
C	4.18694100	0.08111900	-0.38022800
C	2.81654900	0.06197200	0.06632200
C	2.12252000	0.14381900	-2.17719700
H	6.27328300	0.05664500	0.17256000
H	3.60584800	0.22100000	-3.73906500

H	5.50494000	0.15526600	-2.09201100
C	5.25518400	0.04256400	0.55090700
C	2.56570600	0.01708600	1.48156200
H	1.27754100	0.15886200	-2.85630900
C	3.65997200	-0.01913300	2.33848500
C	4.99530200	-0.01083700	1.89750800
H	3.44452500	-0.05176200	3.39933900
H	5.80372400	-0.04236200	2.62107000
C	1.22640800	0.01602200	2.21333800
O	1.23705400	0.01626300	3.44790900
O	0.09609500	0.01458100	1.56611300
H	-5.95225400	-0.19930700	1.00278800
N	1.81005000	0.08791600	-0.88137500
O	-0.71453600	0.01420100	-2.37501000
H	-1.67485800	0.00031100	-2.53639200

Ru^{III}(OOH)(Tpy)(QC)

N	-2.16739100	0.37935300	0.16086700
N	-0.20314900	2.05173500	-0.46922900
N	-1.09510000	-2.02180000	0.04642500
C	-2.50478300	1.68442700	0.20396600
C	-3.79989300	2.04147800	0.58590800
C	-4.70283300	1.03050200	0.92114100
C	-4.31691900	-0.30908600	0.88392600
C	-3.01090000	-0.61795800	0.49312400
C	-1.41075600	2.61881000	-0.16620900
C	-1.57522500	4.00279000	-0.20971100
C	-0.49694100	4.81289700	-0.56732300
C	0.72538600	4.22257200	-0.87516600

C	0.82829400	2.83403200	-0.81332100
C	-2.41368400	-1.96929900	0.41005200
C	-3.12122400	-3.13839300	0.68934300
C	-2.47512600	-4.37068600	0.60072900
C	-1.13127400	-4.40923100	0.23672400
C	-0.47741200	-3.20914500	-0.03207800
H	-4.10561800	3.07928200	0.62923000
H	-5.01858900	-1.08695200	1.15836400
H	-2.53229000	4.44974200	0.02973100
H	-0.61724100	5.89044600	-0.60369300
H	1.58827400	4.81420600	-1.15836300
H	1.75688700	2.32511200	-1.04432700
H	-4.16573300	-3.09150800	0.97218500
H	-3.01806200	-5.28503000	0.81547900
H	-0.58986200	-5.34499000	0.15848900
H	0.56720900	-3.18525700	-0.31945600
Ru	-0.27755100	-0.10179400	-0.34777400
O	-0.69369200	-0.21230500	-2.25642800
C	3.34646300	-1.32784600	-2.48371100
C	4.41451900	-1.10279400	-1.65252400
C	4.19264500	-0.61529200	-0.34231200
C	2.84506100	-0.37269200	0.10715400
C	2.06014200	-1.04019000	-2.00226200
H	6.29752800	-0.55839500	0.13246000
H	3.46716700	-1.70260300	-3.49360500
H	5.43329000	-1.29001100	-1.97930000
C	5.29754600	-0.36777800	0.51120100
C	2.65372400	0.08536700	1.45630700
H	1.19681500	-1.17152900	-2.64387400

C	3.78040800	0.31520400	2.23681500
C	5.09533400	0.10461300	1.78402600
H	3.60760900	0.66148500	3.24852200
H	5.93325200	0.30340000	2.44461200
C	1.34110300	0.30514300	2.20063900
O	1.38669200	0.71287600	3.36466600
O	0.20288600	0.01446500	1.63958800
H	-5.71216000	1.29081400	1.22096100
N	1.80108600	-0.57906700	-0.77716200
O	-2.03331900	-0.64023300	-2.56206500
H	-1.98681300	-0.67391200	-3.53637200

Ru^{IV}(O)(Tpy)(QC)

N	2.28094900	-0.05744200	0.00430500
N	0.63271900	-2.09290500	-0.42132500
N	0.79219800	2.11566700	-0.30505100
C	2.83584200	-1.27452000	0.16928800
C	4.16939100	-1.36062600	0.57663100
C	4.87462700	-0.17858500	0.80697900
C	4.26069400	1.06444500	0.64705900
C	2.92529400	1.10294400	0.23819800
C	1.92149000	-2.41305900	-0.09315900
C	2.32044800	-3.74611600	-0.00941700
C	1.39256200	-4.75777800	-0.25951600
C	0.08404000	-4.41604100	-0.58807900
C	-0.25533400	-3.06594300	-0.65956600
C	2.09916200	2.31991800	0.04286300
C	2.59436500	3.61281800	0.20660000
C	1.74478100	4.70306300	0.01536200

C	0.41661000	4.47863300	-0.33480100
C	-0.02064800	3.16373800	-0.48602000
H	4.65164100	-2.31942200	0.71981000
H	4.81396400	1.97390300	0.84421600
H	3.34222700	-3.99822200	0.24621500
H	1.69486300	-5.79770000	-0.19695800
H	-0.66799800	-5.17016700	-0.78892200
H	-1.25867400	-2.74592500	-0.91446700
H	3.63007200	3.77372400	0.47939600
H	2.12229600	5.71234500	0.14001800
H	-0.27691900	5.29654900	-0.49194100
H	-1.04359800	2.93412700	-0.76002800
Ru	0.32567600	0.02971900	-0.52061900
O	0.68446200	0.06046100	-2.29166400
C	-3.51009300	0.30974700	-2.60460100
C	-4.51434400	0.25159900	-1.67214100
C	-4.19237500	0.12693300	-0.30009700
C	-2.81034100	0.06499600	0.10848100
C	-2.18477900	0.23860500	-2.15335800
H	-6.26426900	0.11379400	0.30208900
H	-3.70812200	0.40447400	-3.66595400
H	-5.55924700	0.29845500	-1.96464800
C	-5.23838200	0.06274100	0.65507200
C	-2.53010000	-0.05863200	1.51348100
H	-1.36434300	0.27374600	-2.86030500
C	-3.60302200	-0.12006100	2.39463100
C	-4.94844200	-0.06193100	1.99031600
H	-3.35935100	-0.21492300	3.44577800
H	-5.73968600	-0.11360800	2.73139000

C	-1.17224500	-0.12120200	2.20561400
O	-1.14268700	-0.26616500	3.43154000
O	-0.08386900	-0.00263400	1.51097600
H	5.91069900	-0.22682600	1.12391700
N	-1.83040000	0.12191000	-0.87051500

Ru^{IV}(OH)(Tpy)(QC)

N	-2.26756800	0.10675800	0.07190300
N	-0.56907800	2.03297000	-0.57097400
N	-0.85762300	-2.12791600	-0.16607500
C	-2.77884100	1.35239900	0.15588500
C	-4.10914100	1.50476300	0.54835700
C	-4.85377500	0.35929500	0.83814100
C	-4.28797600	-0.91328700	0.73998000
C	-2.95341400	-1.02274200	0.34487700
C	-1.83158400	2.43082700	-0.20683000
C	-2.16931500	3.78066600	-0.20805700
C	-1.21617600	4.72735500	-0.58886100
C	0.05562600	4.30602500	-0.96626700
C	0.34294300	2.94364700	-0.94556500
C	-2.17387400	-2.27210300	0.19114400
C	-2.71164800	-3.54068900	0.38904400
C	-1.89986500	-4.66444600	0.22260100
C	-0.56573000	-4.49849600	-0.13714000
C	-0.07887000	-3.20665400	-0.32353100
H	-4.56095900	2.48521000	0.62827800
H	-4.87815700	-1.79099000	0.96995200
H	-3.16431300	4.09608800	0.08028400
H	-1.47336900	5.78095400	-0.59248300

H	0.82097900	5.00867200	-1.27342700
H	1.31595800	2.56195800	-1.22985100
H	-3.75103900	-3.65859100	0.66868100
H	-2.31249300	-5.65578600	0.37455100
H	0.09714700	-5.34463600	-0.27338800
H	0.95159400	-3.02268700	-0.60121700
Ru	-0.31748100	-0.08005700	-0.42002400
O	-0.72217500	-0.25970100	-2.29528600
C	3.45853100	-0.88324000	-2.48940700
C	4.48109300	-0.70019900	-1.59101000
C	4.18695200	-0.31409200	-0.26265600
C	2.81806400	-0.12517400	0.13319800
C	2.14400200	-0.67411100	-2.05327500
H	6.26107700	-0.26546400	0.32791700
H	3.63597300	-1.17637900	-3.51721300
H	5.51740800	-0.84313200	-1.88174600
C	5.24093900	-0.10993000	0.66512700
C	2.56323500	0.26724200	1.48797800
H	1.31417800	-0.79674900	-2.73846700
C	3.63723800	0.46321300	2.34997500
C	4.97270500	0.27864100	1.95542500
H	3.41134300	0.76487600	3.36604900
H	5.77465000	0.43920700	2.66774500
C	1.22607800	0.48303600	2.12044600
O	1.09616200	0.99103200	3.22069600
O	0.13877500	0.04266300	1.47216800
H	-5.88888300	0.45993800	1.14497700
N	1.81985700	-0.31011500	-0.80725800
H	-1.06629800	0.54451500	-2.73085500

$Ru^{V}(O)(Tpy)(QC)$

N	2.25977800	-0.32596400	-0.01485700
N	0.27502200	-2.00843600	-0.55262600
N	1.15511300	2.07443600	-0.10960200
C	2.59701200	-1.63576100	0.03212200
C	3.90401800	-1.97348500	0.37740500
C	4.80697800	-0.94685800	0.66780800
C	4.41568000	0.39225100	0.62758000
C	3.09870800	0.69327000	0.27890200
C	1.49724300	-2.57300900	-0.28977600
C	1.65442500	-3.95419600	-0.33493200
C	0.55852300	-4.76047700	-0.65285700
C	-0.67206600	-4.16985300	-0.92147700
C	-0.77568300	-2.78174800	-0.86205400
C	2.48449900	2.03517400	0.21464600
C	3.17699500	3.21290600	0.48255700
C	2.50113400	4.43320400	0.42524800
C	1.14737500	4.45084600	0.10486500
C	0.50394900	3.24196200	-0.15850200
H	4.21925200	-3.00765400	0.42728400
H	5.12253700	1.17552400	0.86935600
H	2.61827700	-4.40298100	-0.12991700
H	0.67459200	-5.83799100	-0.69037400
H	-1.54465500	-4.75962200	-1.17546500
H	-1.70876900	-2.27276600	-1.06897900
H	4.22928300	3.18472500	0.73607900
H	3.03281800	5.35527700	0.63294100
H	0.58570000	5.37595100	0.05366700
H	-0.54779900	3.20190200	-0.41392200

Ru	0.35422400	0.12541400	-0.49380600
O	0.56428000	0.35154800	-2.20913400
C	-3.45168500	1.37485700	-2.37586500
C	-4.46360800	1.14818600	-1.47525900
C	-4.15821500	0.62363400	-0.19817200
C	-2.79048400	0.35042100	0.14433400
C	-2.14004900	1.06295500	-1.99642600
H	-6.21994400	0.57811500	0.43420500
H	-3.63621800	1.77075700	-3.36724700
H	-5.49845100	1.35804800	-1.72742100
C	-5.19881700	0.35859300	0.73038200
C	-2.52597600	-0.17039300	1.45242600
H	-1.33020300	1.20765700	-2.70112800
C	-3.58348100	-0.42473900	2.31807600
C	-4.92012300	-0.16875100	1.96901600
H	-3.34495700	-0.82361400	3.29759400
H	-5.71477100	-0.37805600	2.67666700
C	-1.17603700	-0.43252800	2.01823800
O	-0.97708400	-1.09675400	3.02024300
O	-0.15040500	0.17663000	1.41420400
H	5.82708900	-1.19622800	0.93796400
N	-1.80217700	0.57176200	-0.79949300

$Ru^{II}(H_2O)(TpyCl)(Bpy)$

Ru	0.44263200	-0.00011300	-0.53588900
N	0.01640500	-2.09546100	-0.48272500
N	-1.55025900	0.00012200	-0.21639700
N	0.01709600	2.09531000	-0.48251200
N	1.08656300	-0.00027300	1.44768200

N	2.55334800	-0.00018100	-0.75695600
C	0.87564800	-3.11850900	-0.61785000
H	1.91159000	-2.84868700	-0.78648500
C	0.47580800	-4.45102400	-0.54712200
H	1.21190100	-5.23842500	-0.66246600
C	-0.86948200	-4.73563700	-0.32691800
H	-1.21804200	-5.76093200	-0.26395200
C	-1.77058000	-3.68098000	-0.18739500
H	-2.82079200	-3.88386000	-0.01583400
C	-1.31023700	-2.36684700	-0.26882700
C	-2.19587400	-1.18626800	-0.13837400
C	-3.57937400	-1.21689200	0.04374700
H	-4.12305100	-2.15002700	0.10869300
C	-4.25393900	0.00055300	0.13526400
C	-3.57897700	1.21778100	0.04381700
H	-4.12236500	2.15108300	0.10879300
C	-2.19548000	1.18672000	-0.13829500
C	-1.30948300	2.36704200	-0.26862700
C	-1.76945800	3.68129700	-0.18706200
H	-2.81961700	3.88445800	-0.01551900
C	-0.86806200	4.73572000	-0.32642300
H	-1.21633700	5.76110600	-0.26335700
C	0.47715800	4.45075400	-0.54659500
H	1.21347900	5.23796200	-0.66180400
C	0.87662800	3.11813700	-0.61746800
H	1.91250200	2.84805400	-0.78610000
C	0.27628400	-0.00038900	2.52280800
H	-0.78709000	-0.00027200	2.32087000
C	0.75830800	-0.00063000	3.82631500

H	0.05952300	-0.00072500	4.65497200
C	2.13696200	-0.00074900	4.02986500
H	2.55216200	-0.00095800	5.03201100
C	2.98075200	-0.00060100	2.92323600
H	4.05426000	-0.00069400	3.06310600
C	2.43951800	-0.00034900	1.63477700
C	3.25841500	-0.00017900	0.40746900
C	4.65652700	0.00000100	0.39994400
H	5.21342100	0.00001100	1.32858100
C	5.33506600	0.00017700	-0.81576600
H	6.41983000	0.00033000	-0.83314500
C	4.60200300	0.00014800	-2.00234900
H	5.08963700	0.00027500	-2.97063000
C	3.21326500	-0.00004700	-1.92967800
H	2.59202600	-0.00011900	-2.81804300
Cl	-5.98631700	0.00082900	0.36340100
O	0.13102600	-0.00008400	-2.73249500
H	-0.35406300	0.77518200	-3.06663300
H	-0.35604000	-0.77420700	-3.06640200

$Ru^{III}(H_2O)$(TpyCl)(Bpy)

Ru	0.44028700	-0.00987100	-0.55446600
N	-0.02314500	-2.07331200	-0.30373800
N	-1.58978200	0.00593900	-0.20643300
N	0.00998000	2.05902000	-0.32840700
N	1.16409900	0.00228300	1.39403400
N	2.51992300	-0.00362200	-0.86881900
C	0.87268600	-3.07478200	-0.32666400
H	1.91556000	-2.79462500	-0.40496700

C	0.48196600	-4.40976800	-0.26706100
H	1.23673800	-5.18657800	-0.29572000
C	-0.87405300	-4.70884600	-0.16900600
H	-1.21103600	-5.73828700	-0.11750100
C	-1.80438000	-3.66878100	-0.13051200
H	-2.86213600	-3.88567900	-0.04821600
C	-1.35960100	-2.35267800	-0.19760300
C	-2.24403000	-1.17343700	-0.13633300
C	-3.62997500	-1.19375700	0.00563900
H	-4.17762100	-2.12459800	0.06830000
C	-4.30082200	0.02949100	0.07228900
C	-3.60904300	1.24065900	0.01525200
H	-4.14010300	2.18083100	0.08200400
C	-2.22283900	1.19643700	-0.12656600
C	-1.31914200	2.36028200	-0.19245900
C	-1.74056600	3.68334500	-0.10716700
H	-2.79254200	3.91642000	0.00020600
C	-0.79453700	4.70789300	-0.15901200
H	-1.11276700	5.74246300	-0.09319300
C	0.55369100	4.38643100	-0.28968100
H	1.32050400	5.15076800	-0.33145000
C	0.92113200	3.04576300	-0.36594200
H	1.95724600	2.74980500	-0.46935100
C	0.38307400	0.01177500	2.48912300
H	-0.68623700	0.02271100	2.32636400
C	0.91655600	0.00414000	3.77208000
H	0.24970200	0.01161000	4.62585700
C	2.30155000	-0.01445000	3.92271300
H	2.75140900	-0.02195300	4.90937800

C	3.10969100	-0.02344500	2.78890900
H	4.18703100	-0.03756100	2.89014000
C	2.52321000	-0.01414800	1.52317800
C	3.28225900	-0.01379800	0.26367700
C	4.67402600	-0.01655500	0.17896200
H	5.28131400	-0.02598500	1.07490000
C	5.28160500	-0.00436500	-1.07543900
H	6.36348600	-0.00632500	-1.15140100
C	4.48963600	0.01482700	-2.22338400
H	4.92604800	0.03063300	-3.21487600
C	3.10798300	0.01509200	-2.08123800
H	2.44437500	0.04090600	-2.93688400
Cl	-6.02836300	0.04346600	0.24199000
O	-0.03027900	0.00576200	-2.68271600
H	-0.88132200	0.39486800	-2.95874900
H	0.01413400	-0.86620600	-3.11943500

Ru^{III}(OH)(TpyCl)(Bpy)

Ru	0.41069900	-0.00006200	-0.57579000
N	-0.00139300	-2.09167900	-0.39681800
N	-1.58799500	-0.00003300	-0.24555700
N	-0.00141700	2.09161200	-0.39703800
N	1.16202400	0.00018700	1.44383000
N	2.51839500	-0.00010400	-0.84984000
C	0.87806100	-3.10151300	-0.47296800
H	1.91783900	-2.82435200	-0.59727000
C	0.48423900	-4.43570700	-0.40349500
H	1.23058100	-5.21854100	-0.47135100
C	-0.86845500	-4.72655100	-0.24825200

H	-1.21125200	-5.75393500	-0.18982000
C	-1.78688200	-3.67974600	-0.16727400
H	-2.84219800	-3.89107500	-0.04637100
C	-1.33472300	-2.36404400	-0.24425800
C	-2.22746300	-1.18649300	-0.15796700
C	-3.61149600	-1.21788400	0.01551300
H	-4.15323500	-2.15137200	0.08921100
C	-4.28831900	-0.00003800	0.09462800
C	-3.61151800	1.21781100	0.01533700
H	-4.15328100	2.15129600	0.08889600
C	-2.22748400	1.18642400	-0.15813400
C	-1.33475300	2.36397900	-0.24457400
C	-1.78693200	3.67968800	-0.16778800
H	-2.84225600	3.89103200	-0.04698000
C	-0.86851000	4.72649300	-0.24885400
H	-1.21132600	5.75388000	-0.19058500
C	0.48419500	4.43564300	-0.40397600
H	1.23053600	5.21847400	-0.47187800
C	0.87803100	3.10144100	-0.47326000
H	1.91781900	2.82427500	-0.59747200
C	0.40621800	0.00037300	2.55587100
H	-0.66699900	0.00037200	2.40839100
C	0.95628400	0.00056200	3.83231300
H	0.30584000	0.00071400	4.69908200
C	2.34440200	0.00054700	3.95755200
H	2.81271200	0.00068700	4.93609100
C	3.13168800	0.00035800	2.80897200
H	4.21050400	0.00035100	2.89539800
C	2.52058600	0.00018300	1.55267600

C	3.27446700	-0.00000400	0.28098800
C	4.66974600	-0.00006800	0.20139000
H	5.27468200	-0.00000900	1.09915700
C	5.28396700	-0.00021000	-1.04882700
H	6.36644500	-0.00026100	-1.12045000
C	4.49410200	-0.00028800	-2.19771800
H	4.93352600	-0.00038400	-3.18855700
C	3.11044100	-0.00023700	-2.05613400
H	2.43673900	-0.00030800	-2.90569400
Cl	-6.01790900	-0.00003400	0.30626200
O	0.13020900	-0.00019100	-2.51319100
H	-0.81130800	-0.00018200	-2.76860500

Ru^{III}(OOH)(TpyCl)(Bpy)

Ru	-0.44288000	-0.00999600	-0.50246000
N	-0.06555600	2.10039900	-0.38442100
N	1.54860100	0.03029100	-0.12498700
N	0.00335300	-2.08831000	-0.32534200
N	-1.17502100	-0.00382700	1.50811600
N	-2.55988000	-0.01864000	-0.76715300
C	-0.95103900	3.09881100	-0.51456400
H	-1.98282000	2.80664900	-0.66885700
C	-0.57555000	4.43938600	-0.45988200
H	-1.32770300	5.21168600	-0.57111300
C	0.76687200	4.74931200	-0.26178500
H	1.09647700	5.78153100	-0.21263600
C	1.69254900	3.71449700	-0.12454700
H	2.74011400	3.94117200	0.03100500
C	1.25781400	2.39223400	-0.18929700

C	2.16482800	1.22810900	-0.04744100
C	3.54324800	1.28549300	0.15849400
H	4.06980000	2.22817600	0.22542800
C	4.23811000	0.08034900	0.27705800
C	3.58356100	-1.14958400	0.19749700
H	4.13977700	-2.07235900	0.29498500
C	2.20401200	-1.14337700	-0.01103300
C	1.33508900	-2.33635500	-0.12353400
C	1.80852500	-3.64360600	-0.02397400
H	2.86259100	-3.83415800	0.13657900
C	0.91417300	-4.70796600	-0.13324200
H	1.27370700	-5.72852100	-0.05787700
C	-0.43719500	-4.44241700	-0.33818400
H	-1.16629500	-5.23910600	-0.42937800
C	-0.85254100	-3.11599900	-0.42805700
H	-1.89228500	-2.85860900	-0.59012900
C	-0.40554500	-0.00228400	2.61105400
H	0.66550800	-0.00398900	2.45134200
C	-0.93924500	0.00393600	3.89435900
H	-0.27708000	0.00585900	4.75222700
C	-2.32533600	0.00693100	4.03816400
H	-2.78110100	0.01072400	5.02255200
C	-3.12647400	0.00433400	2.89944000
H	-4.20407800	0.00574100	2.99922900
C	-2.53176800	-0.00063000	1.63520600
C	-3.30129300	-0.00565400	0.37387000
C	-4.69773700	0.00308700	0.31592200
H	-5.28788300	0.01361800	1.22339000
C	-5.33292000	-0.00108000	-0.92331700

H	-6.41635200	0.00577200	-0.97767800
C	-4.56078100	-0.01401800	-2.08386100
H	-5.01406700	-0.01799600	-3.06840100
C	-3.17549100	-0.02266900	-1.96236100
H	-2.52357200	-0.03488800	-2.82861200
O	-0.19076600	-0.06347200	-2.44084900
O	1.09808900	-0.42226000	-2.91186300
H	0.93297800	-0.48725700	-3.87330200
Cl	5.95792500	0.11336400	0.53363300

Ru^{IV}(O)(TpyCl)(Bpy)

Ru	-0.41182000	0.00000000	-0.64590700
N	-0.00299500	2.10400200	-0.49856300
N	1.58410900	0.00000000	-0.27227900
N	-0.00299500	-2.10400200	-0.49856300
N	-1.10619700	0.00000000	1.46190100
N	-2.55413100	0.00000000	-0.78575800
C	-0.87345400	3.11562600	-0.61244000
H	-1.90941900	2.84226700	-0.77199800
C	-0.47560000	4.44941100	-0.53449600
H	-1.21555700	5.23496900	-0.63319000
C	0.87091200	4.73450000	-0.33134400
H	1.21686000	5.76027200	-0.26487800
C	1.78106000	3.68303900	-0.21240300
H	2.83204200	3.89034700	-0.05320700
C	1.32531600	2.36991800	-0.30047400
C	2.21478100	1.19005200	-0.18086200
C	3.59444700	1.21976900	0.01781900
H	4.13796000	2.15175100	0.09601800

C	4.26896500	-0.00000100	0.11234100
C	3.59444600	-1.21976900	0.01781900
H	4.13796000	-2.15175200	0.09601900
C	2.21478100	-1.19005300	-0.18086200
C	1.32531500	-2.36991800	-0.30047400
C	1.78105900	-3.68303900	-0.21240300
H	2.83204100	-3.89034700	-0.05320700
C	0.87091000	-4.73450100	-0.33134400
H	1.21685800	-5.76027200	-0.26487800
C	-0.47560100	-4.44941100	-0.53449700
H	-1.21555900	-5.23496900	-0.63319000
C	-0.87345500	-3.11562500	-0.61244000
H	-1.90942000	-2.84226600	-0.77199900
C	-0.31109300	0.00000000	2.54482300
H	0.75678800	0.00000000	2.36177500
C	-0.81241900	0.00000000	3.84195900
H	-0.13000700	0.00000000	4.68382800
C	-2.19438000	0.00000000	4.01818400
H	-2.62693200	0.00000000	5.01299500
C	-3.02315600	0.00000000	2.89885400
H	-4.09781900	0.00000000	3.02561100
C	-2.45609200	0.00000000	1.62189800
C	-3.25753000	0.00000000	0.37838700
C	-4.65488000	0.00000000	0.36016500
H	-5.21746500	0.00000000	1.28476100
C	-5.32781100	0.00000000	-0.85893000
H	-6.41239100	0.00000000	-0.87949000
C	-4.59235300	0.00000000	-2.04257000
H	-5.07455300	0.00000000	-3.01318600

C	-3.20455100	0.00000000	-1.96357200
H	-2.57692100	0.00000000	-2.84705500
O	-0.21048200	0.00000000	-2.43457300
Cl	5.98689600	-0.00000100	0.35622200

Ru^{IV}(OH)(TpyCl)(Bpy)

Ru	-0.41427100	0.03043600	-0.62994700
N	-0.01093300	2.09675600	-0.39281100
N	1.56967800	0.00298800	-0.22847800
N	-0.04656700	-2.05736400	-0.48014400
N	-1.05370800	-0.03138300	1.40994000
N	-2.54558900	0.04634300	-0.78046200
C	-0.90018200	3.10059200	-0.46173200
H	-1.93850700	2.82358100	-0.59213500
C	-0.50410800	4.43330400	-0.38556900
H	-1.25175900	5.21438700	-0.45382400
C	0.84755700	4.72509100	-0.22524000
H	1.18693200	5.75284300	-0.15953200
C	1.77234100	3.68094900	-0.14476100
H	2.82616600	3.89459000	-0.01709200
C	1.32646900	2.36753600	-0.22917900
C	2.21281000	1.19015200	-0.14181100
C	3.59502200	1.20300700	0.01879800
H	4.14786600	2.12975200	0.09548200
C	4.26263400	-0.02606100	0.07693000
C	3.57211400	-1.24070600	-0.01562600
H	4.10838500	-2.17908200	0.03252900
C	2.19053100	-1.19794200	-0.17499200
C	1.28230900	-2.35610500	-0.29649400

C	1.70182500	-3.67887300	-0.22120100
H	2.74857100	-3.91358500	-0.07434200
C	0.76024300	-4.70479400	-0.33295800
H	1.07959100	-5.73938300	-0.27466800
C	-0.58233400	-4.38567100	-0.51424600
H	-1.34308100	-5.15150900	-0.60619700
C	-0.95195000	-3.04493900	-0.57984500
H	-1.98257600	-2.74775000	-0.72564200
C	-0.21739400	-0.06151800	2.46602600
H	0.84270500	-0.04623600	2.25357700
C	-0.68293900	-0.10715600	3.77275000
H	0.02722000	-0.12831500	4.59060100
C	-2.05930100	-0.12328100	3.99253200
H	-2.45897000	-0.15932600	4.99990900
C	-2.92474300	-0.09175500	2.90196800
H	-3.99513100	-0.10201200	3.05963900
C	-2.40466400	-0.04605300	1.60908100
C	-3.23306400	-0.00387200	0.39440500
C	-4.62802700	-0.00733500	0.39577400
H	-5.17660300	-0.04917400	1.32766000
C	-5.31439100	0.04490100	-0.81536600
H	-6.39880100	0.04262200	-0.82294900
C	-4.59516400	0.10265800	-2.00744400
H	-5.08968200	0.14858400	-2.97041200
C	-3.20640800	0.10165300	-1.95246900
H	-2.59635100	0.15046600	-2.84567000
O	-0.16768600	0.07736400	-2.51772500
H	-0.08567500	-0.76900800	-3.00647700
Cl	5.97728500	-0.04550700	0.26921700

$Ru^V(O)(TpyCl)(Bpy)$

Ru	-0.38456700	0.00007600	-0.72588500
N	-0.01474500	2.03306800	-0.34047100
N	1.60903000	0.00009800	-0.30793800
N	-0.01470400	-2.03304400	-0.34052700
N	-1.07194400	-0.00003000	1.36834000
N	-2.52335100	0.00020400	-0.87263200
C	-0.94897400	2.99882900	-0.31935300
H	-1.98151400	2.68978900	-0.41149600
C	-0.59528700	4.34009300	-0.20876700
H	-1.37433900	5.09286900	-0.20546200
C	0.75241900	4.67748400	-0.11404000
H	1.05632900	5.71453300	-0.02546000
C	1.71889500	3.66897300	-0.12740300
H	2.77016400	3.91562500	-0.04675500
C	1.32062100	2.34312900	-0.23591600
C	2.23719000	1.19101400	-0.20701600
C	3.61960000	1.22200300	-0.04930000
H	4.15919100	2.15627200	0.03454200
C	4.30171900	0.00012000	0.00813400
C	3.61958900	-1.22180200	-0.04926000
H	4.15922600	-2.15604300	0.03457700
C	2.23720700	-1.19084500	-0.20701600
C	1.32069100	-2.34299500	-0.23602200
C	1.71904500	-3.66885300	-0.12777200
H	2.77034300	-3.91543300	-0.04726100
C	0.75263000	-4.67742500	-0.11454900
H	1.05657600	-5.71448200	-0.02618100
C	-0.59510000	-4.34010600	-0.20917700

H	-1.37408900	-5.09293800	-0.20590400
C	-0.94883900	-2.99879700	-0.31960000
H	-1.98142100	-2.68993600	-0.41186000
C	-0.26497800	-0.00019300	2.44500100
H	0.80121000	-0.00017600	2.25964700
C	-0.76263600	-0.00041400	3.74151700
H	-0.07535300	-0.00045400	4.57896300
C	-2.14451500	-0.00054600	3.92491200
H	-2.57075700	-0.00075800	4.92204600
C	-2.98129400	-0.00042400	2.81178100
H	-4.05497300	-0.00056200	2.94370200
C	-2.42378000	-0.00015300	1.53247000
C	-3.22531900	-0.00001000	0.29530400
C	-4.62094900	-0.00009100	0.27401700
H	-5.18363400	-0.00030400	1.19820900
C	-5.29124400	0.00008100	-0.94686800
H	-6.37546200	0.00001000	-0.96843600
C	-4.55606400	0.00034100	-2.13040000
H	-5.03653100	0.00052100	-3.10148200
C	-3.16887800	0.00038700	-2.05489700
H	-2.54836600	0.00055900	-2.94254600
O	-0.23849000	-0.00054300	-2.44926300
Cl	6.01716300	0.00006100	0.18541800

$Ru^{II}(H_2O)(TpyMe)(Bpy)$

Ru	0.25678100	-0.00000500	-0.53383900
N	-0.17238600	-2.09592300	-0.47575100
N	-1.73225600	0.00023700	-0.18524500
N	-0.17173600	2.09601900	-0.47529600

N	0.92117100	-0.00044100	1.44128800
N	2.36611600	-0.00012000	-0.77743500
C	0.68308900	-3.12010800	-0.62881600
H	1.71614000	-2.85078800	-0.81574300
C	0.28374400	-4.45207500	-0.55364800
H	1.01644000	-5.24032800	-0.68416100
C	-1.05811900	-4.73464300	-0.30921800
H	-1.40706000	-5.75968200	-0.24215500
C	-1.95476400	-3.67912500	-0.15103300
H	-3.00222700	-3.88003800	0.03885800
C	-1.49582800	-2.36428100	-0.23769000
C	-2.37919400	-1.18326300	-0.08900400
C	-3.75659600	-1.20402700	0.13031200
H	-4.28675700	-2.14566900	0.21323500
C	-4.46502300	0.00062100	0.24276900
C	-3.75625100	1.20501400	0.13063800
H	-4.28608800	2.14680500	0.21382200
C	-2.37879800	1.18386900	-0.08873200
C	-1.49511600	2.36467200	-0.23718300
C	-1.95371900	3.67961900	-0.15030000
H	-3.00113200	3.88077200	0.03960600
C	-1.05680300	4.73493700	-0.30829400
H	-1.40548300	5.76005300	-0.24105400
C	0.28498800	4.45206900	-0.55276500
H	1.01788500	5.24015800	-0.68313200
C	0.68399400	3.12001100	-0.62817300
H	1.71698100	2.85046800	-0.81514000
C	0.12139000	-0.00059800	2.52445000
H	-0.94378600	-0.00046100	2.33179100

C	0.61607000	-0.00090000	3.82317500
H	-0.07443400	-0.00101900	4.65878800
C	1.99675200	-0.00104200	4.01347400
H	2.42157900	-0.00128000	5.01160500
C	2.82967100	-0.00087000	2.89868500
H	3.90449300	-0.00097400	3.02820300
C	2.27593000	-0.00056100	1.61538500
C	3.08278700	-0.00033500	0.38006300
C	4.48094600	-0.00030600	0.35921800
H	5.04674300	-0.00047400	1.28252100
C	5.14802300	-0.00004900	-0.86273900
H	6.23259400	-0.00001900	-0.89047800
C	4.40343700	0.00016300	-2.04230000
H	4.88187600	0.00036000	-3.01520000
C	3.01551000	0.00011600	-1.95616000
H	2.38591200	0.00026800	-2.83872900
C	-5.95712400	0.00059900	0.45115100
H	-6.28277300	0.89282100	0.99331800
H	-6.47506100	-0.00646600	-0.51636800
H	-6.28094400	-0.88516800	1.00497300
O	-0.08716400	0.00053200	-2.72785100
H	-0.58314700	0.77494600	-3.04749300
H	-0.58314100	-0.77374200	-3.04782900

$Ru^{III}(H_2O)(TpyMe)(Bpy)$

Ru	0.25093600	-0.00865200	-0.55278500
N	-0.21578700	-2.07027900	-0.29693300
N	-1.77125300	0.01394200	-0.18277700
N	-0.16970100	2.06134800	-0.32031500

N	0.99466600	0.00050800	1.38793300
N	2.33180900	-0.01044500	-0.88760300
C	0.67680000	-3.07523000	-0.32983400
H	1.71943800	-2.79791400	-0.42131000
C	0.28290800	-4.40815900	-0.26444100
H	1.03422000	-5.18797000	-0.30172200
C	-1.07369000	-4.70177800	-0.15007900
H	-1.41356300	-5.73013200	-0.09404200
C	-1.99950300	-3.65895600	-0.10126700
H	-3.05720700	-3.87106500	-0.00644600
C	-1.55227100	-2.34314900	-0.17409500
C	-2.43246000	-1.16116200	-0.10218700
C	-3.81489700	-1.16705000	0.06268800
H	-4.35341400	-2.10418200	0.13626600
C	-4.51481100	0.04672800	0.14318100
C	-3.78644100	1.24268600	0.07394400
H	-4.30168100	2.19229400	0.15365300
C	-2.40331000	1.20403100	-0.09158200
C	-1.49657400	2.36488900	-0.16786700
C	-1.91156100	3.69041900	-0.07689700
H	-2.96178500	3.92561500	0.04258400
C	-0.96327900	4.71154000	-0.13871300
H	-1.27737200	5.74718300	-0.06847300
C	0.38313400	4.38659100	-0.28515900
H	1.15145800	5.14893100	-0.33516100
C	0.74472100	3.04547100	-0.36731000
H	1.77845900	2.74555700	-0.48330100
C	0.22305900	0.01324800	2.48966800
H	-0.84749000	0.02977500	2.33502400

C	0.76749700	0.00219600	3.76799000
H	0.10823400	0.01244300	4.62764000
C	2.15369100	-0.02330000	3.90653700
H	2.61203200	-0.03369100	4.88930500
C	2.95188700	-0.03540100	2.76573900
H	4.02996100	-0.05472500	2.85802500
C	2.35448000	-0.02243300	1.50496200
C	3.10299100	-0.02448300	0.23852300
C	4.49428000	-0.03259900	0.14339100
H	5.10827900	-0.04512100	1.03471300
C	5.09242800	-0.02160600	-1.11545600
H	6.17369400	-0.02772500	-1.19968100
C	4.29163200	0.00178900	-2.25715400
H	4.72060300	0.01686400	-3.25191200
C	2.91103800	0.00721600	-2.10395100
H	2.24110100	0.03647800	-2.95463800
C	-6.01118200	0.06163800	0.28747600
H	-6.35596600	0.98271100	0.76392800
H	-6.47915000	0.00176600	-0.70337400
H	-6.36130500	-0.79479000	0.87008600
O	-0.25061000	0.01053100	-2.67277700
H	-1.11820000	0.37913400	-2.92435900
H	-0.19820500	-0.85933200	-3.11281500

Ru^{III}(OH)(TpyMe)(Bpy)

Ru	-0.22203500	-0.00000100	-0.57486800
N	0.18991600	2.09117300	-0.38824500
N	1.77175000	0.00000000	-0.22021800
N	0.18991500	-2.09117500	-0.38823900

N	-0.99323500	0.00000500	1.43717400
N	-2.32951100	-0.00000200	-0.86846900
C	-0.68857700	3.10168300	-0.47587100
H	-1.72668200	2.82426500	-0.61365200
C	-0.29602100	4.43527600	-0.40137600
H	-1.04089900	5.21863000	-0.47902300
C	1.05536000	4.72491100	-0.22870800
H	1.39785700	5.75228200	-0.16630200
C	1.97184400	3.67805600	-0.13546700
H	3.02585600	3.88791100	-0.00109100
C	1.52166200	2.36122600	-0.21747900
C	2.41339400	1.18372200	-0.11758700
C	3.79245800	1.20524400	0.08716000
H	4.32068100	2.14704200	0.17660900
C	4.50281200	0.00000000	0.18397300
C	3.79245700	-1.20524500	0.08716200
H	4.32068000	-2.14704300	0.17661100
C	2.41339400	-1.18372300	-0.11758500
C	1.52166100	-2.36122800	-0.21747400
C	1.97184300	-3.67805700	-0.13546100
H	3.02585600	-3.88791200	-0.00108600
C	1.05535900	-4.72491300	-0.22869800
H	1.39785600	-5.75228300	-0.16629000
C	-0.29602200	-4.43527700	-0.40136400
H	-1.04090000	-5.21863200	-0.47900700
C	-0.68857800	-3.10168500	-0.47586100
H	-1.72668300	-2.82426700	-0.61364000
C	-0.24685000	0.00000900	2.55537100
H	0.82752200	0.00000900	2.41593200

C	-0.80770800	0.00001200	3.82718300
H	-0.16473100	0.00001500	4.69954400
C	-2.19691500	0.00001100	3.94057700
H	-2.67355200	0.00001400	4.91512100
C	-2.97439200	0.00000700	2.78534400
H	-4.05394100	0.00000700	2.86279500
C	-2.35247800	0.00000400	1.53417500
C	-3.09533500	0.00000000	0.25570800
C	-4.49003500	-0.00000200	0.16452100
H	-5.10236900	0.00000000	1.05727000
C	-5.09385700	-0.00000600	-1.09071500
H	-6.17571000	-0.00000700	-1.17143900
C	-4.29422900	-0.00000800	-2.23293000
H	-4.72543100	-0.00001100	-3.22739800
C	-2.91182000	-0.00000600	-2.07944100
H	-2.23102700	-0.00000700	-2.92343400
C	5.99637200	-0.00000400	0.37224300
H	6.32849900	-0.88950700	0.91445400
H	6.49716700	-0.00012000	-0.60422200
H	6.32852900	0.88960400	0.91426300
O	0.08421200	-0.00000200	-2.50934200
H	1.03065600	0.00001400	-2.74550100

Ru^{III}(OOH)(TpyMe)(Bpy)

Ru	-0.25557700	-0.00890100	-0.49768800
N	0.08852400	2.10817500	-0.36561300
N	1.72413100	0.06142900	-0.07247800
N	0.21944200	-2.07957700	-0.31422800
N	-1.02942400	-0.02163000	1.49795400

N	-2.36978900	-0.04404800	-0.80416800
C	-0.80732800	3.09479700	-0.51678800
H	-1.83097600	2.78838700	-0.69630300
C	-0.45225600	4.43998700	-0.45239500
H	-1.21165800	5.20236600	-0.58157700
C	0.88116700	4.76726100	-0.22132200
H	1.19550100	5.80392100	-0.16388500
C	1.81686200	3.74515900	-0.06184600
H	2.85746000	3.98475100	0.11906000
C	1.40314300	2.41614500	-0.13751600
C	2.32306700	1.26484200	0.02627300
C	3.69510000	1.33281100	0.27171200
H	4.19340800	2.29118700	0.35485200
C	4.43715200	0.15269100	0.41250200
C	3.76904500	-1.07701800	0.30571700
H	4.32623600	-2.00004200	0.41579000
C	2.39813200	-1.10093000	0.05925000
C	1.54942100	-2.30620800	-0.07799200
C	2.03812500	-3.60763900	0.03227500
H	3.09044900	-3.78149000	0.22083800
C	1.16342700	-4.68484000	-0.10180200
H	1.53627700	-5.70008500	-0.01832400
C	-0.18655000	-4.43993700	-0.34187700
H	-0.90077500	-5.24750400	-0.45317800
C	-0.61846900	-3.12011800	-0.44043300
H	-1.65740200	-2.87748800	-0.62882700
C	-0.28153300	-0.01733700	2.61555600
H	0.79235700	-0.00663200	2.47588500
C	-0.84009500	-0.02312400	3.88831800

H	-0.19474000	-0.01826200	4.75891900
C	-2.22870900	-0.03579000	4.00523000
H	-2.70354800	-0.04179400	4.98059600
C	-3.00739400	-0.04161700	2.85108100
H	-4.08671100	-0.05267300	2.93013000
C	-2.38814900	-0.03388300	1.59853600
C	-3.13308200	-0.04230400	0.32229200
C	-4.52832500	-0.04801200	0.23794000
H	-5.13548000	-0.04610700	1.13416000
C	-5.13999300	-0.05560200	-1.01302800
H	-6.22222400	-0.06007200	-1.08793400
C	-4.34567300	-0.05744800	-2.15864300
H	-4.78022500	-0.06387400	-3.15159800
C	-2.96293500	-0.05157800	-2.01065800
H	-2.29437300	-0.05460600	-2.86426500
O	0.03640200	-0.05313600	-2.42949600
O	1.32838900	-0.43932000	-2.87702200
H	1.17774400	-0.50258900	-3.84073600
C	5.91873000	0.19152200	0.67722600
H	6.15276400	-0.30361300	1.62649800
H	6.46440900	-0.34147700	-0.10953500
H	6.29105700	1.21758700	0.72195900

Ru^{IV}(O)(TpyMe)(Bpy)

Ru	-0.22153500	0.00000100	-0.64806500
N	0.18553400	2.10381500	-0.49377800
N	1.76556100	0.00000000	-0.24583800
N	0.18553100	-2.10381400	-0.49377700
N	-0.93263900	0.00000100	1.45564700

N	-2.36677300	0.00000100	-0.80132300
C	-0.68310800	3.11578500	-0.62354900
H	-1.71611700	2.84191200	-0.80087200
C	-0.28705600	4.44905700	-0.53978800
H	-1.02473600	5.23498600	-0.65189600
C	1.05646200	4.73325700	-0.31346700
H	1.40155600	5.75909600	-0.24207700
C	1.96380100	3.68196900	-0.17784000
H	3.01221600	3.88817300	-0.00104600
C	1.51056000	2.36776700	-0.27220100
C	2.39774300	1.18775400	-0.13529800
C	3.77070600	1.20765600	0.10097000
H	4.29969400	2.14803400	0.19777800
C	4.47618700	-0.00000200	0.21629500
C	3.77070400	-1.20766000	0.10096900
H	4.29969100	-2.14803800	0.19777600
C	2.39774200	-1.18775600	-0.13529800
C	1.51055600	-2.36776800	-0.27220100
C	1.96379600	-3.68197000	-0.17783900
H	3.01221000	-3.88817600	-0.00104500
C	1.05645500	-4.73325700	-0.31346500
H	1.40154800	-5.75909700	-0.24207500
C	-0.28706200	-4.44905600	-0.53978600
H	-1.02474400	-5.23498300	-0.65189300
C	-0.68311300	-3.11578300	-0.62354700
H	-1.71612100	-2.84190900	-0.80087000
C	-0.14426300	0.00000100	2.54333800
H	0.92465300	0.00000100	2.36609700
C	-0.65358300	0.00000300	3.83740000

H	0.02341700	0.00000300	4.68364900
C	-2.03665600	0.00000300	4.00499500
H	-2.47536500	0.00000400	4.99713300
C	-2.85839400	0.00000200	2.88051000
H	-3.93381900	0.00000300	3.00067600
C	-2.28330100	0.00000100	1.60699100
C	-3.07719600	0.00000100	0.35833600
C	-4.47454600	0.00000000	0.33215400
H	-5.04263500	0.00000000	1.25340800
C	-5.14038100	-0.00000100	-0.89081300
H	-6.22484500	-0.00000200	-0.91772800
C	-4.39788500	-0.00000100	-2.07009700
H	-4.87448000	-0.00000100	-3.04350400
C	-3.01050100	0.00000000	-1.98266900
H	-2.37741300	0.00000000	-2.86230800
O	-0.00752000	0.00000000	-2.43488500
C	5.96401500	-0.00000700	0.43902400
H	6.28397100	-0.88995700	0.98744800
H	6.48441200	-0.00011700	-0.52724600
H	6.28400700	0.89003900	0.98726900

Ru^{IV}(OH)(TpyMe)(Bpy)

Ru	0.22539600	-0.02702300	-0.63113300
N	-0.18415200	-2.09157000	-0.39642900
N	-1.74923900	0.00851300	-0.20852700
N	-0.13060000	2.06128800	-0.47272800
N	0.87978700	0.02431100	1.40696700
N	2.35967900	-0.05286700	-0.79272400
C	0.70081000	-3.09931700	-0.47672900

H	1.73834000	-2.82582700	-0.62039900
C	0.30099700	-4.42962900	-0.39587000
H	1.04418000	-5.21404500	-0.47407700
C	-1.05059800	-4.71556800	-0.21859000
H	-1.39303500	-5.74212400	-0.14891100
C	-1.96964800	-3.66847100	-0.12595200
H	-3.02278000	-3.87687800	0.01567900
C	-1.52065800	-2.35558500	-0.21506200
C	-2.40077600	-1.17493500	-0.11246400
C	-3.77806300	-1.17203600	0.07215600
H	-4.32354700	-2.10422600	0.15615800
C	-4.47093900	0.05008200	0.14956500
C	-3.74643100	1.24835300	0.04375800
H	-4.26599900	2.19666200	0.10351900
C	-2.36711200	1.20919300	-0.14046000
C	-1.45640500	2.36375300	-0.27265900
C	-1.86872900	3.68910700	-0.19083100
H	-2.91300000	3.92681600	-0.03157200
C	-0.92457600	4.71080900	-0.31147900
H	-1.23882000	5.74673700	-0.24810500
C	0.41536800	4.38698600	-0.50805400
H	1.17778400	5.15026700	-0.60722200
C	0.77829300	3.04549700	-0.58025800
H	1.80581500	2.74361300	-0.73824600
C	0.05019300	0.05482200	2.46821200
H	-1.01118700	0.04642900	2.26154000
C	0.52375300	0.09309400	3.77242400
H	-0.18121500	0.11498100	4.59474300
C	1.90138300	0.10123200	3.98392400

H	2.30744100	0.13141700	4.98895200
C	2.75990900	0.06939800	2.88793500
H	3.83124900	0.07358600	3.03920000
C	2.23169800	0.03111600	1.59797900
C	3.05349300	-0.01088200	0.37826700
C	4.44858500	-0.01508600	0.37252000
H	5.00253300	0.02008700	1.30151200
C	5.12832100	-0.06634900	-0.84240200
H	6.21269700	-0.07004900	-0.85565200
C	4.40255900	-0.11532100	-2.03092900
H	4.89190500	-0.15998700	-2.99661300
C	3.01408400	-0.10690200	-1.96822700
H	2.39854900	-0.14830000	-2.85811800
O	-0.03022000	-0.06320800	-2.51841300
H	-0.11416800	0.78938000	-2.99520300
C	-5.95926900	0.06047500	0.34457900
H	-6.45162600	-0.48454100	-0.46882800
H	-6.22220900	-0.44761900	1.27939600
H	-6.35437300	1.07758000	0.37535200

Ru^{V}(O)(TpyMe)(Bpy)

Ru	-0.19985100	0.02922700	-0.71414700
N	0.07156200	2.07359600	-0.28634100
N	1.76885600	0.10082300	-0.26857200
N	0.27833200	-2.03844700	-0.49364500
N	-0.88110900	-0.18413400	1.35332400
N	-2.36275600	0.03389100	-0.83134500
C	-0.89964100	3.00508200	-0.27184400
H	-1.91710200	2.65713700	-0.38855100

C	-0.60242700	4.35611500	-0.13023500
H	-1.40900000	5.07923700	-0.12916700
C	0.72867600	4.74185600	0.00701900
H	0.99029400	5.78781300	0.12286100
C	1.73412300	3.77127200	0.00236100
H	2.77233500	4.05727100	0.11425500
C	1.38900800	2.43423400	-0.14153900
C	2.34985900	1.31638800	-0.12392500
C	3.72365500	1.39460200	0.04965900
H	4.20900000	2.35630100	0.16562000
C	4.49302000	0.21430400	0.08032900
C	3.84024700	-1.02164000	-0.04388000
H	4.41292900	-1.93956300	0.00165500
C	2.46020300	-1.06449200	-0.21740700
C	1.61878400	-2.26660700	-0.32674400
C	2.09776800	-3.57004400	-0.23544200
H	3.15642200	-3.75249400	-0.09871900
C	1.20083200	-4.63686100	-0.31257600
H	1.56589600	-5.65543100	-0.24279100
C	-0.15718300	-4.38032100	-0.47691300
H	-0.88530700	-5.17946600	-0.54774600
C	-0.58769700	-3.05778800	-0.56657300
H	-1.63057100	-2.80928700	-0.71606800
C	-0.04463000	-0.29993600	2.40254700
H	1.01630700	-0.28271200	2.19391600
C	-0.51134200	-0.42113600	3.70466200
H	0.19858800	-0.50322300	4.51894900
C	-1.88759500	-0.42714700	3.92382900
H	-2.28834800	-0.52030900	4.92714300

C	-2.75149600	-0.31301700	2.83791600
H	-3.82197000	-0.31700600	2.99479900
C	-2.22680300	-0.19645900	1.55024200
C	-3.05030700	-0.08292700	0.33611000
C	-4.44591300	-0.08848100	0.33610900
H	-4.99451800	-0.18678500	1.26361900
C	-5.13262800	0.03353900	-0.86940600
H	-6.21702700	0.03145900	-0.87721300
C	-4.41219200	0.15580800	-2.05550600
H	-4.90488500	0.25001800	-3.01587500
C	-3.02364400	0.15010800	-1.99864100
H	-2.41590100	0.23500100	-2.89092600
O	-0.19626200	-0.20948000	-2.42193600
C	5.98170800	0.28924200	0.24728400
H	6.41776100	-0.69621900	0.42097900
H	6.43412400	0.71404200	-0.65751900
H	6.24403200	0.94977100	1.08017100

$Ru^{II}(H_2O)(TpyMeO)(Bpy)$

Ru	0.45705000	-0.00990700	-0.53805100
N	0.18141900	-2.13146700	-0.46142400
N	-1.53630000	-0.15240400	-0.22196100
N	-0.13310400	2.04841200	-0.51261700
N	1.08624300	0.06621400	1.44602700
N	2.56267400	0.14468500	-0.74957700
C	1.11344800	-3.09095700	-0.58699800
H	2.12719500	-2.74758800	-0.75765100
C	0.81283100	-4.44809400	-0.50492400
H	1.60433400	-5.18090000	-0.61328300

C	-0.50872200	-4.82779200	-0.28270800
H	-0.78176400	-5.87531200	-0.21116000
C	-1.48406300	-3.84040800	-0.15269000
H	-2.51713900	-4.11677100	0.02011200
C	-1.12249500	-2.49632300	-0.24570400
C	-2.09636500	-1.38344400	-0.12719600
C	-3.46497600	-1.51347200	0.05668800
H	-3.94924400	-2.47858100	0.13586700
C	-4.25762900	-0.35239800	0.13756900
C	-3.65536600	0.91094800	0.02537200
H	-4.24631100	1.81435500	0.07457700
C	-2.27364700	0.97587800	-0.15743700
C	-1.47752500	2.21976600	-0.30259800
C	-2.03396000	3.49742300	-0.23710500
H	-3.09637100	3.62315100	-0.06742300
C	-1.21553200	4.61567300	-0.38913300
H	-1.64113900	5.61223200	-0.33843500
C	0.14782500	4.43164200	-0.60582100
H	0.82213900	5.27124000	-0.73064000
C	0.64627100	3.13237100	-0.65997500
H	1.69995600	2.93962100	-0.82506600
C	0.27070500	0.01956400	2.51640900
H	-0.78765300	-0.06803500	2.30655400
C	0.74183100	0.07874800	3.82252200
H	0.03935700	0.03698700	4.64708900
C	2.11483400	0.19130200	4.03445400
H	2.52130900	0.24090900	5.03898800
C	2.96402900	0.23907700	2.93303800
H	4.03322000	0.32593900	3.07948100

C	2.43378800	0.17478300	1.64134300
C	3.25850300	0.21668500	0.41862300
C	4.65297200	0.32089600	0.41893200
H	5.20182000	0.37706700	1.35072100
C	5.33819700	0.35199700	-0.79252400
H	6.42006200	0.43260000	-0.80382400
C	4.61502000	0.27813200	-1.98310800
H	5.10799300	0.29906900	-2.94851600
C	3.22978400	0.17534100	-1.91810100
H	2.61642300	0.11458800	-2.81001700
O	-5.57155600	-0.55765200	0.31691000
C	-6.45083300	0.57265200	0.40868000
H	-7.44619200	0.15349200	0.55155000
H	-6.18832500	1.20206300	1.26517600
H	-6.42671400	1.16142100	-0.51395100
O	0.14129500	-0.06590400	-2.73752900
H	-0.42979100	0.64919500	-3.06955300
H	-0.27135600	-0.89041800	-3.05018400

$Ru^{III}(H_2O)$(TpyMeO)(Bpy)

Ru	0.45092500	-0.00718100	-0.56595000
N	-0.17474500	-2.03135200	-0.32608200
N	-1.57004800	0.15862400	-0.22768600
N	0.17703000	2.08653200	-0.32908300
N	1.15422400	-0.05962300	1.38993400
N	2.53720200	-0.14508000	-0.85455200
C	0.64373200	-3.09723200	-0.35024800
H	1.70512000	-2.89458200	-0.42065100
C	0.15416100	-4.39897600	-0.30086700

H	0.84737800	-5.23114000	-0.32943000
C	-1.22177000	-4.59390800	-0.21382500
H	-1.63682800	-5.59487900	-0.17145500
C	-2.07099700	-3.48710600	-0.17495500
H	-3.14237700	-3.62530800	-0.10124400
C	-1.52917700	-2.20677400	-0.23002600
C	-2.32095500	-0.96124100	-0.16254700
C	-3.70080000	-0.88202500	-0.01156400
H	-4.29696800	-1.78114400	0.04790200
C	-4.29606700	0.39067700	0.07485000
C	-3.48452400	1.54304700	0.02152400
H	-3.95331000	2.51502300	0.10579800
C	-2.11714600	1.39606100	-0.13070600
C	-1.12460200	2.48732400	-0.18821300
C	-1.43980000	3.83942800	-0.08875700
H	-2.47046200	4.15210800	0.02279200
C	-0.41711300	4.78684500	-0.13059000
H	-0.65385700	5.84237200	-0.05318700
C	0.90275500	4.36333900	-0.26646400
H	1.72638300	5.06643700	-0.30093900
C	1.16320500	2.99997100	-0.35785600
H	2.17263000	2.62424000	-0.46610300
C	0.36144300	0.00190600	2.47485200
H	-0.70090000	0.10018200	2.29650600
C	0.87449800	-0.06085700	3.76463000
H	0.19847600	-0.00881800	4.60965700
C	2.25141700	-0.19155300	3.93322700
H	2.68551000	-0.24564800	4.92549600
C	3.07210600	-0.25128700	2.81004500

H	4.14361100	-0.35032700	2.92518100
C	2.50570400	-0.18214500	1.53680300
C	3.28035900	-0.22397000	0.28721900
C	4.66974800	-0.32646800	0.22217700
H	5.26133400	-0.39041300	1.12638300
C	5.29545200	-0.34314000	-1.02305200
H	6.37540900	-0.42233100	-1.08396400
C	4.52394900	-0.25329700	-2.18147900
H	4.97469800	-0.25734400	-3.16670300
C	3.14386800	-0.15551600	-2.05761900
H	2.49712200	-0.07223800	-2.92264200
O	-5.60090200	0.60884100	0.21788200
C	-6.50883800	-0.50687600	0.29092300
H	-7.49734600	-0.06252300	0.39450500
H	-6.46164000	-1.10197600	-0.62553800
H	-6.28575900	-1.12682500	1.16405300
O	-0.02209400	0.05957600	-2.69694300
H	-0.87663300	0.45869300	-2.94713400
H	0.00237900	-0.80597900	-3.14760000

Ru^{III}(OH)(TpyMeO)(Bpy)

Ru	0.43717900	0.00001100	-0.59325700
N	0.01607300	-2.09173800	-0.43520000
N	-1.57618500	0.00009000	-0.36050800
N	0.01624300	2.09179100	-0.43513700
N	1.09286800	-0.00005000	1.46042800
N	2.55628700	-0.00007100	-0.76723400
C	0.89788400	-3.10236300	-0.46824500
H	1.94292900	-2.82543500	-0.53691100

C	0.50052600	-4.43613900	-0.42444700
H	1.24878500	-5.21950000	-0.45565800
C	-0.85916400	-4.72608000	-0.34207500
H	-1.20518700	-5.75349000	-0.30599100
C	-1.77945800	-3.67885300	-0.30536900
H	-2.84021600	-3.88764400	-0.24056800
C	-1.32364300	-2.36275400	-0.35318800
C	-2.22088100	-1.18625400	-0.30726200
C	-3.61015500	-1.21489300	-0.20120300
H	-4.16347200	-2.14486700	-0.16386700
C	-4.30547700	0.00019800	-0.15478400
C	-3.61005700	1.21523100	-0.20116500
H	-4.16331200	2.14524100	-0.16377600
C	-2.22078400	1.18648400	-0.30723500
C	-1.32345100	2.36291300	-0.35312400
C	-1.77916000	3.67904800	-0.30527100
H	-2.83990200	3.88792100	-0.24047000
C	-0.85878200	4.72620100	-0.34194500
H	-1.20472200	5.75363800	-0.30583500
C	0.50088500	4.43615300	-0.42431800
H	1.24920700	5.21945500	-0.45550500
C	0.89813700	3.10234600	-0.46815000
H	1.94315900	2.82533700	-0.53682100
C	0.28597300	-0.00003800	2.53595800
H	-0.77919400	0.00000500	2.33824800
C	0.77557700	-0.00007900	3.83683800
H	0.08513300	-0.00006700	4.67217900
C	2.15635200	-0.00013300	4.02692300
H	2.57850800	-0.00016600	5.02621500

C	2.99657300	-0.00014600	2.91652000
H	4.07025400	-0.00018900	3.05305000
C	2.44475900	-0.00010400	1.63290600
C	3.25795000	-0.00011600	0.39805200
C	4.65552000	-0.00017100	0.38484800
H	5.21711800	-0.00020700	1.31036200
C	5.32829900	-0.00017900	-0.83478000
H	6.41297400	-0.00022200	-0.85505200
C	4.59391100	-0.00013200	-2.01995100
H	5.07988900	-0.00013700	-2.98885600
C	3.20510100	-0.00007800	-1.94396800
H	2.57232100	-0.00004000	-2.82453400
O	-5.66359600	0.00028600	-0.11537500
C	-6.25229000	-0.00032700	1.20226500
H	-7.33085400	-0.00019600	1.04504400
H	-5.95649900	-0.89675100	1.75794100
H	-5.95639600	0.89553800	1.75878900
O	0.24241800	0.00004400	-2.54161900
H	-0.68883400	0.00006500	-2.83211400

Ru^{III}(OOH)(TpyMeO)(Bpy)

Ru	-0.45139100	-0.02222500	-0.50528400
N	0.07732600	2.06387100	-0.42599900
N	1.53711200	-0.10910100	-0.12502100
N	-0.14673800	-2.12080400	-0.29323900
N	-1.17471100	0.06515500	1.50745300
N	-2.56836500	0.12067100	-0.76069700
C	-0.73461900	3.12078700	-0.57418600
H	-1.78456600	2.90041600	-0.72570800

C	-0.26600300	4.43211800	-0.54046800
H	-0.96118000	5.25410700	-0.66572200
C	1.09497000	4.64830100	-0.34396800
H	1.49766100	5.65494800	-0.31012100
C	1.94421800	3.55251400	-0.18844700
H	3.00481200	3.70724700	-0.03336000
C	1.41835200	2.26258600	-0.23302500
C	2.24217800	1.03755400	-0.07060500
C	3.62042100	1.00746500	0.13141100
H	4.19008200	1.92447900	0.17531000
C	4.24960700	-0.24161800	0.27596500
C	3.48285400	-1.42213000	0.21779900
H	3.98495600	-2.37421400	0.33339400
C	2.11584900	-1.32482500	0.01198100
C	1.16386500	-2.45567900	-0.08039900
C	1.54414200	-3.79099800	0.04601200
H	2.58219000	-4.04868100	0.21666400
C	0.57978300	-4.79308500	-0.04953000
H	0.86768900	-5.83460100	0.04592300
C	-0.74962200	-4.43909300	-0.26699100
H	-1.53130500	-5.18543400	-0.34837900
C	-1.07139600	-3.08953600	-0.38190300
H	-2.09044000	-2.76407500	-0.55340900
C	-0.40063800	0.02257400	2.60615400
H	0.66537700	-0.07098200	2.43970200
C	-0.92384500	0.09660200	3.89177200
H	-0.25844700	0.05971100	4.74639600
C	-2.30409300	0.21689800	4.04219500
H	-2.75164200	0.27715400	5.02853600

C	-3.11012600	0.25781600	2.90763700
H	-4.18340000	0.34919000	3.01265100
C	-2.52562300	0.18007100	1.64080100
C	-3.30047500	0.21045900	0.38282700
C	-4.69282800	0.32274400	0.33114500
H	-5.27515800	0.39333500	1.24101900
C	-5.33389300	0.34370300	-0.90484300
H	-6.41409800	0.43055500	-0.95460500
C	-4.57145900	0.25188500	-2.06831300
H	-5.02956900	0.26385700	-3.05060000
C	-3.19001800	0.14126600	-1.95261600
H	-2.54547900	0.06353800	-2.82103100
O	5.56152500	-0.41249100	0.47353000
C	6.42086500	0.73699700	0.54409900
H	7.42161600	0.33795500	0.70395400
H	6.39186200	1.30090400	-0.39334900
H	6.13979100	1.38036100	1.38371400
O	-0.20759100	-0.12873100	-2.44095600
O	1.00046300	-0.72162700	-2.90064600
H	0.81812200	-0.78988000	-3.85841400

Ru^{IV}(O)(TpyMeO)(Bpy)

Ru	-0.44027800	0.00001400	-0.66454600
N	-0.02546800	2.10443500	-0.53892400
N	1.56918200	-0.00002000	-0.38320000
N	-0.02554200	-2.10441900	-0.53894700
N	-1.03356700	0.00000600	1.47581100
N	-2.58945500	0.00003900	-0.69960700
C	-0.90009500	3.11665300	-0.61410400

H	-1.94281900	2.84335700	-0.72190100
C	-0.49818900	4.44999900	-0.56144900
H	-1.24132600	5.23603700	-0.62729800
C	0.85733400	4.73438300	-0.42538700
H	1.20672800	5.76023100	-0.38049200
C	1.77104800	3.68268100	-0.34619600
H	2.82908500	3.88759600	-0.23920900
C	1.31102000	2.36922200	-0.40644400
C	2.20599200	1.19025200	-0.32550800
C	3.59202500	1.21734900	-0.19577700
H	4.14725500	2.14583300	-0.15493900
C	4.28495500	-0.00007000	-0.13530300
C	3.59198200	-1.21746300	-0.19578600
H	4.14718100	-2.14596500	-0.15495200
C	2.20595000	-1.19031600	-0.32551900
C	1.31093600	-2.36925400	-0.40646400
C	1.77091700	-3.68273000	-0.34622600
H	2.82894600	-3.88768300	-0.23923600
C	0.85716600	-4.73439900	-0.42543200
H	1.20652400	-5.76026000	-0.38054500
C	-0.49834600	-4.44996500	-0.56149900
H	-1.24150900	-5.23597800	-0.62736000
C	-0.90020600	-3.11660600	-0.61414200
H	-1.94292000	-2.84327200	-0.72194100
C	-0.18742600	-0.00001300	2.51927300
H	0.87056900	-0.00000800	2.28532600
C	-0.62580100	-0.00003700	3.83907600
H	0.09677800	-0.00005200	4.64683000
C	-1.99765900	-0.00004000	4.08183300

H	-2.38184300	-0.00005900	5.09633400
C	-2.87917600	-0.00001800	3.00360100
H	-3.94660900	-0.00002000	3.18202100
C	-2.37406400	0.00000500	1.70069200
C	-3.23495500	0.00002900	0.49738700
C	-4.63161300	0.00004200	0.54783900
H	-5.14772800	0.00003400	1.49911500
C	-5.36374800	0.00006700	-0.63663200
H	-6.44803900	0.00007800	-0.60387000
C	-4.68716800	0.00007900	-1.85486400
H	-5.21632000	0.00009900	-2.80073000
C	-3.29716800	0.00006400	-1.84382200
H	-2.71347100	0.00007100	-2.75694900
O	5.63921000	-0.00010100	-0.07517400
C	6.21467800	0.00001000	1.25001500
H	7.29431600	-0.00002800	1.10224200
H	5.91264800	0.89655000	1.80154400
H	5.91260500	-0.89641500	1.80170600
O	-0.32848800	0.00002400	-2.46064600

Ru^{IV}(OH)(TpyMeO)(Bpy)

Ru	-0.41630100	0.03327500	-0.63530700
N	-0.17417200	2.12071000	-0.39399200
N	1.54920600	0.15257900	-0.23609800
N	0.10300200	-2.02071800	-0.47853400
N	-1.05303300	-0.06457700	1.40993400
N	-2.54667100	-0.12025000	-0.78289900
C	-1.14330200	3.04942000	-0.45322300
H	-2.15587600	2.68871100	-0.58201500

C	-0.85634100	4.40810500	-0.37069300
H	-1.66378900	5.12778200	-0.43224900
C	0.46912400	4.80624000	-0.21263300
H	0.72464900	5.85781200	-0.14257000
C	1.47472500	3.84058500	-0.13907600
H	2.50817300	4.13644600	-0.00958500
C	1.13666300	2.49520200	-0.23092300
C	2.11173000	1.38912100	-0.14682000
C	3.47735900	1.50448100	0.01007900
H	3.97222700	2.46421700	0.08491500
C	4.26305900	0.32839900	0.07095400
C	3.64490700	-0.93903600	-0.02289700
H	4.22856500	-1.84692200	0.02528400
C	2.26912400	-0.99400100	-0.18062400
C	1.44967000	-2.21984000	-0.29433000
C	1.96259400	-3.50896400	-0.20985300
H	3.02362000	-3.66754500	-0.06310600
C	1.09671600	-4.60015600	-0.31089500
H	1.48984700	-5.60846600	-0.24350400
C	-0.26586900	-4.38053900	-0.49396200
H	-0.96913400	-5.20033300	-0.57818600
C	-0.73021600	-3.07073200	-0.57106300
H	-1.77952800	-2.84946400	-0.71813600
C	-0.21822300	-0.02041300	2.46629900
H	0.83762300	0.07590800	2.25249300
C	-0.67993100	-0.09196800	3.77349300
H	0.02868900	-0.05091600	4.59198000
C	-2.05081800	-0.21530000	3.99268400
H	-2.44748500	-0.27522100	5.00015900

C	-2.91484800	-0.25990000	2.90141800
H	-3.98123200	-0.35336200	3.05905600
C	-2.39836700	-0.18201900	1.60821200
C	-3.22785700	-0.21076200	0.39266500
C	-4.61888200	-0.31857700	0.39618800
H	-5.16273700	-0.39116900	1.32896200
C	-5.30749300	-0.33178500	-0.81480400
H	-6.38869400	-0.41577100	-0.82123300
C	-4.59476200	-0.23463800	-2.00827400
H	-5.09193400	-0.23838000	-2.97100600
C	-3.20971900	-0.12960600	-1.95456500
H	-2.60269100	-0.04635700	-2.84748200
O	5.56322000	0.51922000	0.21809300
C	6.46436700	-0.60731800	0.29265500
H	7.45419200	-0.16793200	0.39932200
H	6.23051500	-1.22282000	1.16522800
H	6.41210100	-1.19704400	-0.62613600
O	-0.20942600	0.09692800	-2.52919000
H	0.05101200	-0.72263100	-2.99887000

Ru^{V}(O)(TpyMeO)(Bpy)

Ru	-0.40638200	0.00265500	-0.73792200
N	0.07850900	2.06865100	-0.52315600
N	1.50914100	-0.12071500	-0.31297300
N	-0.17657900	-2.11698700	-0.61476000
N	-0.95963000	-0.04912500	1.40788800
N	-2.57872500	0.18762800	-0.65406900
C	-0.75191600	3.11476500	-0.62528600
H	-1.79655500	2.89497000	-0.80381400

C	-0.29167300	4.42621400	-0.51140000
H	-0.99534200	5.24518400	-0.60049000
C	1.06263400	4.64583000	-0.28505200
H	1.45215800	5.65327100	-0.19078800
C	1.92701000	3.55322700	-0.17711400
H	2.98379700	3.71011700	-0.00119200
C	1.41458300	2.26681100	-0.30005800
C	2.22903400	1.03959300	-0.19941400
C	3.59276600	0.97943400	-0.00616800
H	4.17488500	1.88581900	0.07569000
C	4.21283300	-0.29286100	0.07944300
C	3.43348800	-1.47546700	-0.03234900
H	3.93553800	-2.43231500	0.02881700
C	2.07802400	-1.36946400	-0.22686400
C	1.12010900	-2.48191100	-0.37561900
C	1.47066200	-3.82452500	-0.28210700
H	2.49638700	-4.11137800	-0.08736100
C	0.48426700	-4.79989700	-0.44272200
H	0.74647300	-5.84970800	-0.37247800
C	-0.82802900	-4.41084200	-0.69173300
H	-1.62166500	-5.13624300	-0.82467300
C	-1.12449100	-3.05106600	-0.77079300
H	-2.13030200	-2.70069400	-0.96509300
C	-0.05912600	-0.18574700	2.40182600
H	0.98202200	-0.26303000	2.12425200
C	-0.43196500	-0.22419800	3.73938700
H	0.33257600	-0.33204900	4.49956400
C	-1.78236700	-0.12054000	4.06274900
H	-2.11017500	-0.14656000	5.09613800

C	-2.71412200	0.01631800	3.03775000
H	-3.76779800	0.09548600	3.27042800
C	-2.27859600	0.04827200	1.71272100
C	-3.18163600	0.18346400	0.56162200
C	-4.56793000	0.30638700	0.66756400
H	-5.04392000	0.30233000	1.63918300
C	-5.33650500	0.43710900	-0.48557700
H	-6.41412300	0.53323500	-0.41189400
C	-4.70271700	0.44325600	-1.72595400
H	-5.25789000	0.54191600	-2.65130500
C	-3.32057400	0.31673700	-1.77438700
H	-2.78429500	0.31434800	-2.71448200
O	5.49910800	-0.48093500	0.26188000
C	6.40788900	0.64024800	0.39675600
H	7.38914800	0.18753200	0.51975000
H	6.38303000	1.25291200	-0.50723200
H	6.14692800	1.22717300	1.28042600
O	-0.58648400	-0.00183500	-2.45422300

$Ru^{II}(H_2O)(TpyCl)(QC)$

N	-1.85505600	-0.07048300	-0.16113100
N	-0.52203100	2.19348200	-0.23506300
N	-0.07664500	-1.95337800	-0.60444100
C	-2.60990400	1.02380000	0.09055000
C	-3.96740400	0.88343500	0.38518800
C	-4.49856600	-0.40550700	0.41986200
C	-3.70725800	-1.52794200	0.17868500
C	-2.35685200	-1.32588200	-0.11293000
C	-1.86202000	2.30135900	0.03096700

C	-2.45769200	3.54760700	0.23002800
C	-1.67879800	4.70170000	0.16239800
C	-0.31663500	4.58289200	-0.10433000
C	0.21890600	3.31142500	-0.29639500
C	-1.35899700	-2.38606100	-0.38346700
C	-1.67655300	-3.74441500	-0.42181300
C	-0.67645000	-4.67886800	-0.68546300
C	0.62391400	-4.23150800	-0.90843500
C	0.87850300	-2.86307400	-0.85939600
H	-4.59664500	1.73952000	0.58984400
H	-4.13615800	-2.52018500	0.22593900
H	-3.51874000	3.62192100	0.43581300
H	-2.13291600	5.67487000	0.31627900
H	0.32812900	5.45242900	-0.16437400
H	1.27304200	3.16885600	-0.50549800
H	-2.69430800	-4.07432200	-0.25082800
H	-0.91420800	-5.73690700	-0.71655400
H	1.43380800	-4.92135300	-1.11726700
H	1.87418600	-2.46881500	-1.02660400
Ru	0.10826100	0.16664300	-0.48132400
C	4.01517800	0.86042300	-2.30492000
C	4.97028300	0.51888500	-1.38100400
C	4.57477300	0.12695100	-0.07912700
C	3.17390000	0.10030600	0.25938700
C	2.66519000	0.77304000	-1.92832800
H	6.60240000	-0.21069300	0.58090200
H	4.26968000	1.17359900	-3.31120500
H	6.02830200	0.54330500	-1.62572900
C	5.55739100	-0.23380900	0.87688100

C	2.80587400	-0.23888000	1.60702800
H	1.88784700	0.99183700	-2.65039300
C	3.81995100	-0.58223100	2.49331000
C	5.18315100	-0.60039100	2.14596100
H	3.51758200	-0.82653700	3.50429400
H	5.92567800	-0.88399400	2.88538900
C	1.41873800	-0.20442800	2.24969500
O	1.34627000	-0.39148200	3.47288700
O	0.35639600	0.03748700	1.55280500
N	2.24257300	0.40027400	-0.71847000
O	-0.21740600	0.33248900	-2.68911300
H	-0.94821100	0.93370800	-2.91602100
H	-0.45435400	-0.52127400	-3.09214300
Cl	-6.19672500	-0.61990900	0.78656000

$Ru^{III}(H_2O)(TpyCl)(QC)$

N	-1.85919800	-0.08288600	-0.13780900
N	-0.61868600	2.23779800	-0.09755700
N	0.03246400	-1.86597700	-0.68760400
C	-2.65845200	0.96559200	0.14458800
C	-4.01492300	0.74766400	0.39019600
C	-4.48172800	-0.56612300	0.34083900
C	-3.63140500	-1.63992100	0.05838200
C	-2.28763400	-1.36174900	-0.18200700
C	-1.96393400	2.27215000	0.16422800
C	-2.60693200	3.47926900	0.42768900
C	-1.86980100	4.66375600	0.42700000
C	-0.50404000	4.61470700	0.16384300
C	0.08564700	3.37853500	-0.09297400

C	-1.23212400	-2.36005000	-0.49490800
C	-1.48271600	-3.72461300	-0.60161600
C	-0.43436800	-4.59480200	-0.90977800
C	0.84204900	-4.08033000	-1.11055100
C	1.03374100	-2.70482600	-0.99161700
H	-4.69091500	1.56124000	0.61665700
H	-4.01824500	-2.64982200	0.03383000
H	-3.67012000	3.50111600	0.63261000
H	-2.36177300	5.60835100	0.63186900
H	0.10686200	5.50968900	0.15763300
H	1.14629300	3.28972300	-0.29459800
H	-2.48192900	-4.11378500	-0.45122600
H	-0.62272900	-5.65955900	-0.99373900
H	1.68221900	-4.71893300	-1.35639900
H	2.00733500	-2.25477300	-1.14332300
Ru	0.11500500	0.26605300	-0.41415400
C	3.99352100	1.35081800	-2.11267000
C	4.94892900	0.86889800	-1.25032500
C	4.55534700	0.24752200	-0.04138500
C	3.15708900	0.13821600	0.26792300
C	2.64387200	1.17988100	-1.77352300
H	6.57712800	-0.17250100	0.58855400
H	4.25030900	1.83892200	-3.04525500
H	6.00663500	0.95631800	-1.47958200
C	5.52844900	-0.26661100	0.85388300
C	2.78008500	-0.45386700	1.51714800
H	1.86805700	1.51489600	-2.45161800
C	3.77829000	-0.94826000	2.34900100
C	5.14520600	-0.87076900	2.02743200

H	3.46867600	-1.39274700	3.28763200
H	5.88408200	-1.27135200	2.71323700
C	1.39360300	-0.53838900	2.09337800
O	1.17353100	-1.14873300	3.12873800
O	0.40110400	0.14866300	1.51982000
N	2.22962900	0.58962600	-0.64912500
O	-0.21074000	0.51636100	-2.54656100
H	-1.05848700	0.89588800	-2.84149400
H	-0.04471100	-0.26132700	-3.11066100
Cl	-6.16565700	-0.87930100	0.64307600

Ru^{III}(OH)(TpyCl)(QC)

N	-1.88261100	-0.00013000	-0.15854800
N	-0.30227700	2.10132800	-0.38383600
N	-0.30003900	-2.09995100	-0.38629600
C	-2.51063500	1.18585000	-0.01734700
C	-3.87916300	1.21660400	0.25593800
C	-4.54678600	-0.00187300	0.38256200
C	-3.87786500	-1.21949600	0.25433000
C	-2.50938700	-1.18697000	-0.01892400
C	-1.62531000	2.36700500	-0.15767000
C	-2.07917900	3.68169700	-0.06155100
C	-1.17151600	4.73242300	-0.19617600
C	0.17210400	4.44737400	-0.42397600
C	0.56598000	3.11365300	-0.51132900
C	-1.62287700	-2.36711100	-0.16078700
C	-2.07558700	-3.68234200	-0.06667700
C	-1.16695400	-4.73207100	-0.20264200
C	0.17645000	-4.44550800	-0.42974200

C	0.56914000	-3.11129800	-0.51508200
H	-4.41513100	2.14892700	0.37372400
H	-4.41288700	-2.15251400	0.37091500
H	-3.12715100	3.89030500	0.11590200
H	-1.51579600	5.75848300	-0.12271000
H	0.90983900	5.23378400	-0.53362300
H	1.59847100	2.83694400	-0.68849600
H	-3.12340300	-3.89218100	0.11023300
H	-1.51034400	-5.75854100	-0.13074400
H	0.91490900	-5.23110000	-0.54036800
H	1.60142200	-2.83341300	-0.69163100
Ru	0.09829500	0.00108600	-0.50802700
C	3.97473900	0.00542000	-2.50784300
C	4.94967500	0.00376900	-1.54289200
C	4.58371900	0.00121700	-0.17545100
C	3.18878200	0.00048400	0.18749600
C	2.63292500	0.00446600	-2.09597300
H	6.63261000	0.00001800	0.50266200
H	4.20841000	0.00735600	-3.56639500
H	6.00381000	0.00435700	-1.80480700
C	5.59358100	-0.00064700	0.81943100
C	2.85301000	-0.00211300	1.58570300
H	1.83126000	0.00556500	-2.82570200
C	3.89320900	-0.00395200	2.50823000
C	5.25279300	-0.00325500	2.14892200
H	3.61383200	-0.00597500	3.55462300
H	6.01596400	-0.00474600	2.92067000
C	1.47195800	-0.00294400	2.23407000
O	1.40503500	-0.00636500	3.46654300

O	0.38353400	0.00040100	1.51787700
N	2.24180700	0.00213000	-0.82033900
O	-0.18772600	0.00176600	-2.46246100
H	-1.13626600	0.00392000	-2.68342100
Cl	-6.25860400	-0.00301900	0.72377200

Ru^{III}(OOH)(TpyCl)(QC)

N	1.83727400	-0.07964300	-0.05182900
N	0.07233200	-1.99379900	-0.55647600
N	0.45991700	2.16189400	-0.07398900
C	2.34447500	-1.32826900	-0.03862000
C	3.69443500	-1.52069300	0.25780400
C	4.46866000	-0.39299100	0.53647400
C	3.92192400	0.88975600	0.53525500
C	2.56473900	1.01791500	0.23279300
C	1.36169500	-2.39967900	-0.34507000
C	1.70392700	-3.74882300	-0.41928600
C	0.71794000	-4.69269100	-0.71002000
C	-0.58975200	-4.26688200	-0.92224300
C	-0.86940600	-2.90377700	-0.83686300
C	1.79539400	2.28227800	0.20083200
C	2.36307000	3.53245700	0.44281600
C	1.55775900	4.67043800	0.40732700
C	0.19938500	4.53367900	0.13322000
C	-0.31029900	3.25851600	-0.10169600
H	4.13838100	-2.50692900	0.27982300
H	4.53772600	1.74794700	0.76921600
H	2.72617100	-4.06771300	-0.25662200
H	0.97574200	-5.74473000	-0.76952900

H	-1.38581200	-4.96594800	-1.15095000
H	-1.87045200	-2.52071000	-0.99685100
H	3.42107500	3.62289900	0.65642000
H	1.99056100	5.64754800	0.59339200
H	-0.46256100	5.39121600	0.09870000
H	-1.35964300	3.09860900	-0.31965700
Ru	-0.12890200	0.15105400	-0.42609700
O	0.13726300	0.32220000	-2.35798700
C	-4.01587300	0.85152400	-2.31704300
C	-4.98771000	0.54713400	-1.39794000
C	-4.61676600	0.14645200	-0.09217900
C	-3.22104400	0.07120500	0.25995800
C	-2.67377100	0.73231000	-1.92560200
H	-6.66234900	-0.11197600	0.54562900
H	-4.25150500	1.16524700	-3.32746300
H	-6.04236400	0.60559700	-1.65091000
C	-5.62264500	-0.17850100	0.85269600
C	-2.88277100	-0.30750600	1.60489500
H	-1.88094500	0.93671500	-2.63546300
C	-3.91801100	-0.61965200	2.47827300
C	-5.27767000	-0.56830700	2.12266900
H	-3.63463000	-0.90314700	3.48448100
H	-6.03810400	-0.82426300	2.85354700
C	-1.50332000	-0.37021700	2.25154400
O	-1.42410000	-0.71650900	3.43362100
O	-0.43831400	-0.02230800	1.58881800
N	-2.27469400	0.35681000	-0.70870000
O	1.41589300	0.85087500	-2.74773000
H	1.29326600	0.91037700	-3.71428100

Cl	6.16242400	-0.59600400	0.90776500

$Ru^{IV}(O)(TpyCl)(QC)$

N	1.89485900	-0.00684300	-0.19212800
N	0.26500700	-2.07335500	-0.50885600
N	0.35013200	2.13764700	-0.39378400
C	2.48528800	-1.21145300	-0.07034500
C	3.84615800	-1.27828300	0.23161500
C	4.53462300	-0.07632000	0.40486300
C	3.89553300	1.16000000	0.29699200
C	2.53320600	1.16445700	-0.00566500
C	1.57735000	-2.36988500	-0.26158600
C	2.00420100	-3.69430900	-0.18865000
C	1.07980600	-4.72411400	-0.37006300
C	-0.25169400	-4.40716800	-0.61980200
C	-0.61875700	-3.06336700	-0.68172900
C	1.67256600	2.36716200	-0.13021700
C	2.15134500	3.66703500	0.02091600
C	1.26857800	4.74140500	-0.09865200
C	-0.07412800	4.49241400	-0.36521800
C	-0.49382000	3.17011700	-0.50607600
H	4.36166400	-2.22344800	0.33684400
H	4.44829400	2.07689000	0.45107700
H	3.04360400	-3.92800300	0.00640200
H	1.40317200	-5.75804100	-0.31553000
H	-1.00191700	-5.17547900	-0.76626400
H	-1.64174200	-2.76322100	-0.87497500
H	3.19881000	3.84701300	0.22955100
H	1.63232900	5.75672900	0.01676500

H	-0.79343900	5.29695300	-0.46495000
H	-1.52778400	2.92236400	-0.71469000
Ru	-0.09165100	0.04342100	-0.58860500
O	0.15725100	0.08275300	-2.37854600
C	-4.04956100	0.24129500	-2.43507200
C	-4.99329700	0.16129500	-1.44290300
C	-4.58540800	0.04627900	-0.09307000
C	-3.18007700	0.01767900	0.23075300
C	-2.69799500	0.20080500	-2.06565300
H	-6.61587200	-0.01607900	0.63414300
H	-4.31390500	0.33005000	-3.48240300
H	-6.05495900	0.18369400	-1.67096500
C	-5.56950400	-0.04086400	0.92406100
C	-2.81206400	-0.09439400	1.61628900
H	-1.92368700	0.25276600	-2.82180400
C	-3.82763400	-0.17835300	2.56128600
C	-5.19608700	-0.15459800	2.23938000
H	-3.51833200	-0.26335000	3.59588000
H	-5.93944800	-0.22340700	3.02713600
C	-1.41384100	-0.12279500	2.22461100
O	-1.30593500	-0.24979100	3.44786100
O	-0.37176800	0.00235800	1.46265400
N	-2.26339000	0.09451600	-0.80666400
Cl	6.23555800	-0.12097300	0.78060500

Ru^{IV}(OH)(TpyCl)(QC)

N	1.88888200	0.00227700	-0.13355800
N	0.23097800	-1.98437200	-0.67854300
N	0.38173500	2.18004300	-0.24277500

C	2.45460300	-1.22145700	-0.09467100
C	3.81033200	-1.33058800	0.20579800
C	4.52218800	-0.15201400	0.45065000
C	3.90905700	1.10343100	0.40287400
C	2.54892300	1.15452300	0.10291600
C	1.52942100	-2.33638000	-0.40370300
C	1.91915500	-3.67082200	-0.44228600
C	0.97893700	-4.65167200	-0.76654800
C	-0.33035700	-4.27739200	-1.05217200
C	-0.66799600	-2.92679300	-0.99952100
C	1.71320800	2.37472400	0.02167500
C	2.21421300	3.66040600	0.19957500
C	1.34865700	4.75273100	0.10799600
C	0.00007800	4.53653400	-0.15709200
C	-0.44795700	3.22776700	-0.32716400
H	4.30817800	-2.28996400	0.25070700
H	4.48188300	1.99972500	0.59909400
H	2.94276100	-3.95037900	-0.22620400
H	1.27555000	-5.69432500	-0.79753500
H	-1.08759400	-5.00706500	-1.31289900
H	-1.67216300	-2.58227500	-1.21427600
H	3.26590100	3.81666300	0.40493600
H	1.73181300	5.75806100	0.24391300
H	-0.70430100	5.35632500	-0.23325100
H	-1.48779200	3.00549600	-0.53233300
Ru	-0.09513100	0.11429400	-0.48998200
O	0.17358500	0.32665400	-2.38545700
C	-4.02682300	0.78407400	-2.29458800
C	-4.97900900	0.55187500	-1.33233600

C	-4.58199700	0.16251600	-0.03210100
C	-3.18365300	0.02167000	0.26878100
C	-2.67907900	0.62209600	-1.94992600
H	-6.60830100	0.02816100	0.69549800
H	-4.28392100	1.08109900	-3.30428600
H	-6.03707700	0.65821300	-1.55102900
C	-5.56258300	-0.09250200	0.96129700
C	-2.82393300	-0.37834100	1.59700700
H	-1.90232500	0.78505000	-2.68689400
C	-3.82881800	-0.62568000	2.52655500
C	-5.19370200	-0.48611500	2.22511600
H	-3.52372800	-0.93127800	3.52060500
H	-5.93946500	-0.68486200	2.98707000
C	-1.43972600	-0.55038100	2.13256800
O	-1.21108600	-1.08229200	3.20457900
O	-0.42120400	-0.03722700	1.42636700
N	-2.25855500	0.25664600	-0.73329500
H	0.54945900	-0.44413100	-2.85411600
Cl	6.21331700	-0.25211500	0.82466400

Ru^{V}(O)(TpyCl)(QC)

N	1.89298900	-0.05529200	-0.18362900
N	0.04929200	-1.90663300	-0.64810400
N	0.57221800	2.23299500	-0.16229000
C	2.35419300	-1.32770100	-0.18250300
C	3.70157800	-1.55382200	0.07788900
C	4.51768800	-0.44463700	0.33073300
C	4.00888500	0.85739900	0.34354100
C	2.65209100	1.03140800	0.08429700

C	1.32982800	-2.36104600	-0.45983800
C	1.61003600	-3.72050600	-0.53873800
C	0.57609400	-4.62009100	-0.81249700
C	-0.71489700	-4.14103200	-1.00730400
C	-0.94071100	-2.76837300	-0.91893800
C	1.91465500	2.31220000	0.09400000
C	2.50779200	3.54075600	0.36891400
C	1.71920800	4.69322700	0.38485600
C	0.35550700	4.59147700	0.13099800
C	-0.18579500	3.33459200	-0.13969800
H	4.11705500	-2.55242900	0.09262200
H	4.65783700	1.69575800	0.55792300
H	2.62046200	-4.08275500	-0.39625600
H	0.78783800	-5.68173900	-0.87508700
H	-1.54258200	-4.80452900	-1.22726900
H	-1.92481400	-2.34344000	-1.07167200
H	3.56920500	3.60539900	0.57326300
H	2.17265500	5.65496700	0.59781600
H	-0.29161100	5.46036600	0.13797500
H	-1.24142100	3.20245600	-0.34269400
Ru	-0.06809300	0.22437900	-0.54757700
O	0.01942000	0.48764700	-2.26902100
C	-4.07276500	1.13380300	-2.19057100
C	-5.00676500	0.75431200	-1.25761700
C	-4.58122300	0.22251500	-0.01835400
C	-3.17604000	0.10028800	0.25173100
C	-2.71708800	0.96066800	-1.88468600
H	-6.59020400	-0.08915700	0.70318400
H	-4.34978900	1.54384800	-3.15428400

H	-6.06978100	0.84960500	-1.45590400
C	-5.53707500	-0.19612400	0.94439700
C	-2.78881700	-0.42459800	1.52718600
H	-1.96485500	1.22386200	-2.61836500
C	-3.76566000	-0.83074600	2.42890800
C	-5.13843400	-0.72723300	2.14819800
H	-3.43456000	-1.22630300	3.38253100
H	-5.86761900	-1.05312500	2.88175800
C	-1.39170000	-0.53284100	2.02406300
O	-1.06574300	-1.18191600	3.00228100
O	-0.47615000	0.20283900	1.38376000
N	-2.26751800	0.46546900	-0.72688100
Cl	6.20092300	-0.69430400	0.64965900
