# Supplementary material for: Computational Analysis of Structure – Activity Relationships in Highly Active Homogeneous Ruthenium-based Water Oxidation Catalysts
Source: Catalysts. Author manuscript; Available in PMC 2023 Jun 12. (PMC10260203; doi:10.3390/catal12080863)
Supplement: Supporting Information [file NIHMS1854369-supplement-Supporting_Information.pdf]

SUPPLEMENTARY INFORMATION FOR

Computational Analysis of Structure – Activity Relationships in Highly Active Homogeneous Ruthenium-based Water Oxidation Catalysts

Gabriel Bury and Yulia Pushkar*[a]

[a]Department of Physics and Astronomy, Purdue University, West Lafayette, Indiana 47907, United States

*Corresponding author ypushkar@purdue.edu

Table S1. Comparison of O_2 evolution rates for structurally similar catalysts.

Table S2. Free energies of intermediates computed at the B3LYP level of theory.

Table S3. Redox Potentials of the catalysts, computed from free energies at B3LYP level of theory.

Table S4. Free energies of reaction of WNA processes.

Table S5. Computed E_{RRS} for different intermediates.

Table S6. Correlation (r) for each descriptor energy against the set of E_{RRS}.

Table S7. r^2 for each descriptor energy against the set of E_{RRS}.

Table S8. Pearson's coefficients for each of the descriptor variables correlating with oxygen evolution activity and theoretical overpotential η_{th}.

Table S9. Energetics relevant to Ru^{IV}, Ru^{V}, and peroxide formation correlated to O_2 evolution rate.

Table S10. Parameters relevant to the Ru^{IV}=O state.

Table S11. Parameters relevant to the Ru^{IV}-OH state.

Table S12. Parameters relevant to the Ru^{V}=O state.

Table S13. Parameters relevant to the Ru^{III}-OOH state.

Catalyst	Activity (uM O_2/sec)
Ru(tpy)(QC)	42.3
Ru(tpy-Cl)(QC)	35.0
Ru(EtOtpy)(QC)	24.2
Ru(EtOtpy)(4-pic)$_2$	20.0
Ru(tpy)(4-pic)$_2$	4.3
Ru(tpy-MeO)(bpy)	2.4
Ru(EtOtpy)(bpy)	1.8
Ru(tpy-Me)(bpy)	0.61
Ru(tpy-Cl)(bpy)	0.43
Ru(tpy)(bpy)	0.3

Table S1. Comparison of O_2 evolution rates for structurally similar catalysts. Measurements taken at pH=1, 0.1mM HNO3. 1mM catalyst and 20mM CAN (20 equivalents). Oxygen evolution rate is determined at the linear regime of maximal evolution activity. Oxygen evolution rates of the bda-type catalysts are omitted.

	II - H2O	III - H2O	III - OH	RuIV=O	RuIV-OH	RuV=O	RuIII-OOH
Ru(EtoTpy)(4pic)2	-5990.131396	-5989.920554	-5989.515267	-5988.889962	-5989.283267	-5988.644849	-6064.650068
Ru(Tpy)(4pic)2	-5836.341897	-5836.128191	-5835.725194	-5835.098652	-5835.485202	-5834.849987	-5910.860467
Ru(tpy)(bpy)	-5756.582106	-5756.37033	-5755.963752	-5755.334858	-5755.717629	-5755.084604	-5831.0963
Ru(EtoTpy)(bpy)	-5910.370219	-5910.162585	-5909.753626	-5909.126071	-5909.518848	-5908.882316	-5984.893067
Ru(TpyCl)(QC)	-6310.822704	-6310.634547	-6310.214321	-6309.58827	-6309.997833	-6309.359229	-6385.345772
Ru(EtoTpy)(QC)	-6005.03802	-6004.853036	-6004.430132	-6003.806572	-6004.220132	-6003.581803	-6079.564585
Ru(Tpy)(QC)	-5851.25026	-5851.064257	-5850.643782	-5850.018073	-5850.428357	-5849.79047	-5925.777594
Ru(Tpy-MeO)(Bpy)	-5871.079433	-5870.871024	-5870.451648	-5869.833365	-5870.225866	-5869.590004	-5945.597299
Ru(Tpy-Me)(Bpy)	-5795.877388	-5795.667808	-5795.257599	-5794.629796	-5795.021665	-5794.382743	-5870.393785
Ru(Tpy-Cl)(Bpy)	-6216.154939	-6215.940455	-6215.533731	-6214.933185	-6215.32026	-6214.68006	-6290.69808
Ru(bda)(isoq)2	-6194.147046	-6193.976887	-6193.522783	-6192.894406	-6193.309476	-6192.680631	-6268.666183
Ru(bda)(4pic)2	-5965.538374	-5965.371776	-5964.918894	-5964.286355	-5964.74472	-5964.076006	-6040.056939
H_2O				-76.410958			

Table S2. Free energies of intermediates computed at the B3LYP level of theory. See attached supporting document for DFT coordinates. Note implicit water molecule considerations for species related to WNA processes– see Methods. Final column is energy of optimized water molecule geometry.

	II H2O -- III OH	II H2O -- III H2O	III-H2O -- IV=O	III-H2O -- IV-OH	III OH -- IV O	III-OH -- IV-OH	IV O -- V O	IV O -- IV OH
**Ru(EtoTpy)(4pic)2**	0.686	1.297	0.324	1.261	0.935	1.873	2.230	0.938
**Ru(Tpy)(4pic)2**	0.701	1.375	0.295	1.417	0.969	2.091	2.327	1.121
**Ru(tpy)(bpy)**	0.746	1.323	0.457	1.681	1.033	2.257	2.370	1.224
**Ru(EtoTpy)(bpy)**	0.698	1.210	0.485	1.437	0.997	1.949	2.193	0.952
**Ru(TpyCl)(QC)**	0.475	0.680	0.751	1.246	0.956	1.451	1.793	0.495
**Ru(EtoTpy)(QC)**	0.461	0.594	0.756	1.142	0.888	1.274	1.676	0.386
**Ru(Tpy)(QC)**	0.423	0.621	0.748	1.224	0.946	1.422	1.753	0.476
**Ru(Tpy-MeO)(Bpy)**	1.003	1.231	0.516	1.476	0.744	1.704	2.182	0.959
**Ru(Tpy-Me)(Bpy)**	0.785	1.263	0.526	1.502	1.003	1.980	2.283	0.977
**Ru(Tpy-Cl)(Bpy)**	0.824	1.396	-0.311	0.796	0.262	1.369	2.448	1.107
**Ru(bda)(isoq)2**	0.907	0.190	1.736	2.081	1.019	1.364	1.377	0.345
**Ru(bda)(4pic)2**	0.777	0.093	1.816	0.983	1.132	0.300	1.284	-0.833

Table S3. Redox Potentials of the catalysts, computed from free energies at B3LYP level of theory. Units in V.

	IV O -- III OOH	V O --- III OOH
Ru(EtoTpy)(4pic)2	1.631	-0.599
Ru(Tpy)(4pic)2	1.584	-0.742
Ru(tpy)(bpy)	1.594	-0.776
Ru(EtoTpy)(bpy)	1.443	-0.750
Ru(TpyCl)(QC)	1.701	-0.091
Ru(EtoTpy)(QC)	1.688	0.011
Ru(Tpy)(QC)	1.647	-0.107
Ru(Tpy-MeO)(Bpy)	1.526	-0.656
Ru(Tpy-Me)(Bpy)	1.525	-0.758
Ru(Tpy-Cl)(Bpy)	1.500	-0.948
Ru(bda)(isoq)2	1.313	-0.064
Ru(bda)(4pic)2	1.345	0.062

Table S4. Free energies of reaction of possible WNA processes. Note implicit water molecule considerations for species related to WNA processes– see Methods.

Free Energy of Formation	Ru^{III}-H_2O	Ru^{III}-OH	Ru^{IV}-OH	Ru^{IV}-O	Ru^{V}-O	Ru^{III}-OOH
Ru(EtoTpy)(4pic)2	1.30	0.69	2.56	1.62	3.85	3.25
Ru(Tpy)(4pic)2	1.38	0.70	2.79	1.67	4.00	3.25
Ru(tpy)(bpy)	1.32	0.75	3.00	1.78	4.15	3.37
Ru(EtoTpy)(bpy)	1.21	0.70	2.65	1.70	3.89	3.14
Ru(TpyCl)(QC)	0.68	0.47	1.93	1.43	3.22	3.13
Ru(EtoTpy)(QC)	0.59	0.46	1.74	1.35	3.03	3.04
Ru(Tpy)(QC)	0.62	0.42	1.85	1.37	3.12	3.02
Ru(Tpy-MeO)(Bpy)	1.23	1.00	2.71	1.75	3.93	3.27
Ru(Tpy-Me)(Bpy)	1.26	0.79	2.77	1.79	4.07	3.31
Ru(Tpy-Cl)(Bpy)	1.40	0.82	2.19	1.09	3.53	3.35
Ru(bda)(isoq)2	0.19	0.91	2.27	1.93	3.30	3.24
Ru(bda)(4pic)2	0.09	0.78	1.08	1.91	3.19	3.25

Commented [PYN1]: per Lebedev paper - 3.6V

Commented [GSB2]: To SI?

Table S5: Computed E_{RRS} for different intermediates relative to the Ru^{II}-H_2O state. Units in V and eV.

Commented [GSB3]: See also Tables S7 and S8.

	**Ru^{III}-H_2O**	**Ru^{III}-OH**	**Ru^{IV}-OH**	**Ru^{IV}-O**	**Ru^{V}-O**	**Ru^{III}-OOH**
II H_2O -- III OH	0.21	1.00	0.44	0.88	0.56	0.77
II H_2O -- III H_2O	1.00	0.21	0.88	0.05	0.89	0.46
III-H_2O -- IV=O	-0.92	0.15	-0.71	0.35	-0.64	-0.11
III-H_2O -- IV-OH	0.15	0.57	0.61	0.53	0.39	0.46
III OH -- IV O	-0.30	-0.02	-0.22	0.45	-0.01	0.27
III-OH -- IV-OH	0.90	0.16	0.95	0.03	0.81	0.39
IV O -- III OOH	0.34	-0.70	0.08	-0.85	-0.08	-0.38
IV O -- V O	0.99	0.20	0.89	0.07	0.90	0.48
IV O -- IV OH	0.90	0.15	0.94	-0.05	0.77	0.33
IV OH --- V O	-0.43	-0.02	-0.67	0.20	-0.28	0.03
V O --- III OOH	-0.94	-0.45	-0.92	-0.35	-0.98	-0.64

Table S6. Correlations between changes in free energy of reactions / redox potentials as descriptors against each E_{RRS}. First column describes each descriptor, consistently along each row. First row identifies each E_{RRS}, consistent within each column.

	Ru^{III}-H_2O	Ru^{III}-OH	Ru^{IV}-OH	Ru^{IV}-O	Ru^{V}-O	Ru^{III}-OOH
II H_2O -- III OH	0.04	1.00	0.20	0.78	0.31	0.60
II H_2O -- III H_2O	1.00	0.04	0.77	0.00	0.79	0.21
III-H_2O -- IV=O	0.85	0.02	0.50	0.12	0.41	0.01
III-H_2O -- IV-OH	0.02	0.32	0.37	0.28	0.15	0.21
III OH -- IV O	0.09	0.00	0.05	0.20	0.00	0.07
III-OH -- IV-OH	0.81	0.02	0.91	0.00	0.65	0.15
IV O -- III OOH	0.12	0.49	0.01	0.72	0.01	0.15
IV O -- V O	0.99	0.04	0.79	0.00	0.81	0.23
IV O -- IV OH	0.81	0.02	0.89	0.00	0.59	0.11
IV OH --- V O	0.18	0.00	0.45	0.04	0.08	0.00
V O --- III OOH	0.88	0.20	0.85	0.13	0.96	0.41

Table S7. r^2 of each redox potential or change in free energy as descriptors against each E_{RRS}. First column describes each descriptor, consistently along each row. First row identifies each E_{RRS}, consistent within each column.

	r (overpotential)	r^2 (overpotential)		r (activity)	r^2 (activity)
RuV=O + H2O --> RuIII-OOH	1.000	1.000	RuIII_OOH O2 CD	-0.904	0.818
RuIV=O --> RuV=O	0.982	0.965	RuIV_O O SD	0.899	0.807
RuIII_OOH Ru CD	0.982	0.964	RuIV_OH Ru CD	-0.893	0.798
RuIV_OH Ru SD	0.954	0.910	RuIV_OH O SD	-0.892	0.795
RuIV_O O SD	-0.930	0.866	RuIV_O Ru CD	-0.874	0.764
RuIII_OOH O2 SD	0.906	0.822	RuIV-OH --> RuV=O	-0.873	0.762
RuIV_OH O CD	-0.905	0.819	RuIV=O --> RuV=O	-0.873	0.762
RuIV_OH Ru CD	0.892	0.796	RuIV_O Ru SD	-0.871	0.758
RuIV_O Ru CD	0.886	0.785	RuIII_OOH O2 SD	-0.869	0.754
RuIII_OOH Ru SD	0.866	0.750	RuIII_OOH Ru SD	-0.855	0.732
RuV_O O CD	-0.849	0.721	RuIV_OH O CD	0.853	0.727
RuIV_OH O SD	0.844	0.713	RuV_O Ru CD	-0.851	0.725
RuV_O Ru SD	0.841	0.707	RuIV_OH Ru SD	-0.842	0.709
RuIII_OOH O2 CD	0.777	0.604	RuIV=O --> RuIV-OH	0.829	0.687

RuIV_O O CD	0.769	0.591	RuV_O O CD	0.827	0.683
RuIV-OH --> RuV=O	0.730	0.533	RuIII_OOH Ru CD	-0.803	0.645
RuIV=O --> RuIV-OH	-0.726	0.527	RuV=O + H2O --> RuIII-OOH	-0.780	0.608
RuIV_O Ru SD	0.722	0.521	RuV_O Ru SD	-0.747	0.557
RuV_O Ru CD	0.639	0.408	RuIV_O O CD	-0.740	0.547
RuV_O O SD	-0.360	0.130	RuIII_OOH O1 CD	0.342	0.117
RuIII_OOH O1 CD	-0.190	0.036	RuV_O O SD	0.210	0.044
RuIII_OOH O1 SD	-0.042	0.002	RuIII_OOH O1 SD	-0.163	0.027

[a]CD=charge density. [b]SD=spin density [51].

Table S8. Pearson's coefficients for each of the descriptor variables correlating with oxygen evolution activity and theoretical overpotential η_{th}. Positive r indicates that an increase of the descriptor variable would suggest an increase in the oxygen evolution activity. For peroxide species, oxygen labelling scheme is Ru-O1-O2. Table lists in order of descending correlation. Note implicit water molecule considerations for species related to WNA processes– see Methods.

Commented [GSB4]: I can add some of the linear regression plots for the most promising relationships to SI if appropriate. This section might need some fleshing out.

	**RuIV=O --> RuIV-OH**	**RuIV-OH --> RuV=O**	**RuV=O + H2O --> RuIII-OOH**	**uM O2/s**
Ru(EtoTpy)(4pic)2	0.938	1.292	-0.599	20
Ru(Tpy)(4pic)2	1.121	1.205	-0.742	4.3
Ru(tpy)(bpy)	1.224	1.145	-0.776	0.34
Ru(EtoTpy)(bpy)	0.952	1.241	-0.750	1.8
Ru(EtoTpy)(QC)	0.386	1.290	0.011	24.2
Ru(Tpy)(QC)	0.476	1.278	-0.107	42.3
Ru(Tpy-Cl)(QC)	0.495	1.297	-0.091	35.0
Ru(Tpy-MeO)(Bpy)	0.959	1.223	-0.656	2.4
Ru(Tpy-Me)(Bpy)	0.977	1.306	-0.758	0.61
Ru(Tpy-Cl)(Bpy)	1.107	1.341	-0.948	0.43
r	-0.873	0.281	0.869	

Table S9. Energetics relevant to Ru^{IV}, Ru^{V}, and peroxide formation correlated to O_2 evolution rate. Units in eV.

	Ru^{IV}=O Ru Spin Density	Ru^{IV}=O Ru Charge Density	Ru^{IV}=O O Spin Density	Ru^{IV}=O O Charge Density	uM O2/s
Ru(EtoTpy)(4pic)2	0.988	1.056	0.984	-0.407	20
Ru(Tpy)(4pic)2	0.978	1.054	0.996	-0.040	4.3
Ru(tpy)(bpy)	0.977	1.054	1.002	-0.501	0.34
Ru(EtoTpy)(bpy)	0.989	1.049	0.988	-0.350	1.8
Ru(EtoTpy)(QC)	1.031	0.958	0.928	-0.401	24.2
Ru(Tpy)(QC)	1.020	0.963	0.939	-0.396	42.3
Ru(Tpy-Cl)(QC)	1.013	0.971	0.945	-0.393	35.0
Ru(Tpy-MeO)(Bpy)	0.981	1.054	0.998	-0.345	2.4
Ru(Tpy-Me)(Bpy)	0.981	1.051	0.997	-0.346	0.61
Ru(Tpy-Cl)(Bpy)	0.973	1.061	1.006	-0.341	0.43
r	0.835	-0.840	-0.871	-0.219	

Table S10. Parameters relevant to the Ru^{IV}=O state. Atomic units.

	Ru^{IV}-OH Ru Spin Density	Ru^{IV}-OH Ru Charge Density	Ru^{IV}-OH O Spin Density	Ru^{IV}-OH O Charge Density	uM O2/s
Ru(EtoTpy)(4pic)2	1.406	1.186	0.406	-0.610	20
Ru(Tpy)(4pic)2	1.459	1.198	0.424	-0.597	4.3
Ru(tpy)(bpy)	1.449	1.263	0.430	-0.578	0.34
Ru(EtoTpy)(bpy)	1.454	1.256	0.379	-0.594	1.8
Ru(EtoTpy)(QC)	1.405	1.143	0.304	-0.624	24.2
Ru(Tpy)(QC)	1.418	1.163	0.316	-0.619	42.3
Ru(Tpy-Cl)(QC)	1.408	1.167	0.319	-0.618	35.0
Ru(Tpy-MeO)(Bpy)	1.457	1.259	0.389	-0.593	2.4
Ru(Tpy-Me)(Bpy)	1.453	1.261	0.419	-0.583	0.61
Ru(Tpy-Cl)(Bpy)	1.448	1.270	0.434	-0.091	0.43
r	-0.817	-0.855	-0.801	-0.331	

Table S11. Parameters relevant to the Ru^{IV}-OH state. Atomic units.

	Ru^{V}=O Ru Spin Density	Ru^{V}=O Ru Charge Density	Ru^{V}=O O Spin Density	Ru^{V}=O O Charge Density	uM O2/s

Ru(EtoTpy)(4pic)2	0.462	1.288	0.561	-0.297	20
Ru(Tpy)(4pic)2	0.466	1.307	0.570	-0.288	4.3
Ru(tpy)(bpy)	0.441	1.281	0.570	-0.250	0.34
Ru(EtoTpy)(bpy)	0.458	1.284	0.588	-0.260	1.8
Ru(EtoTpy)(QC)	0.456	1.199	0.647	-0.300	24.2
Ru(Tpy)(QC)	0.453	1.201	0.706	-0.290	42.3
Ru(Tpy-Cl)(QC)	0.448	1.206	0.716	-0.288	35.0
Ru(Tpy-MeO)(Bpy)	0.458	1.285	0.585	-0.260	2.4
Ru(Tpy-Me)(Bpy)	0.450	1.278	0.552	-0.253	0.61
Ru(Tpy-Cl)(Bpy)	0.410	1.284	0.625	-0.249	0.43
r	0.263	-0.660	0.773	-0.760	

Table S12. Parameters relevant to the Ru^{V}=O state. Atomic units.

	**Ru^{III}-OOH Ru Spin Density**	**Ru^{III}-OOH Ru Charge Density**	**Ru^{III}-OOH O1 Spin Density**	**Ru^{III}-OOH O1 Charge Density**	**Ru^{III}-OOH O2 Spin Density**	**Ru^{III}-OOH Ru Spin Density**	**uM O2/s**
Ru(EtoTpy)(4pic)2	0.580	1.050	0.340	-0.337	0.076	0.580	20
Ru(Tpy)(4pic)2	0.567	1.052	0.350	-0.332	0.082	0.567	4.3
Ru(tpy)(bpy)	0.549	1.081	0.363	-0.302	0.090	0.549	0.34
Ru(EtoTpy)(bpy)	0.608	1.110	0.326	-0.302	0.056	0.608	1.8
Ru(EtoTpy)(QC)	0.640	0.992	0.272	-0.351	0.051	0.640	24.2
Ru(Tpy)(QC)	0.634	0.998	0.281	-0.349	0.055	0.634	42.3
Ru(Tpy-Cl)(QC)	0.628	1.005	0.286	-0.348	0.057	-0.398	35.0
Ru(Tpy-MeO)(Bpy)	0.562	1.088	0.346	-0.310	0.086	0.562	2.4
Ru(Tpy-Me)(Bpy)	0.559	1.089	0.349	-0.308	0.088	0.559	0.61
Ru(Tpy-Cl)(Bpy)	0.549	1.097	0.356	-0.304	0.093	0.549	0.43
r	0.790	-0.885	-0.835	-0.877	-0.696	0.790	

Table S13. Parameters relevant to the Ru^{III}-OOH state. Atomic units.
